# Supplementary material for: ‘You feel like you’ve been duped’: is the current system for health professionals declaring potential conflicts of interest in the UK fit for purpose? A mixed methods study
Source: BMJ Open. 2023 Jul 26;13(7):e072996. doi: 10.1136/bmjopen-2023-072996 (PMC10373698; doi:10.1136/bmjopen-2023-072996)
Supplement: Supplementary data [file bmjopen-2023-072996supp001.pdf]

Appendix Table 1

Description of the interviewees by sex, age and self defined role

	Males	Females
Age group		
30-39	1	0
40-49	0	1
50-59	2	4
60-69	2	8
70-79	1	0
80+	2	0
Total	8	13
Status		
Citizen	1	3
Patient	2	2
Long term condition	0	2
Patient activist	2	2
Carer	0	1
Not specified	3	3
Total	8	13

Appendix 1:

Method of searching for declarations/conflicts of interest

General Google search for job title, roles, affiliations - first 10 pages "Firstname Lastname" + "NHS" + "private" + "clinic" + "press release"

"Firstname lastname" + "Conflicts of interest" + "declared interests"

Disclosure UK (APBI)

NHS Trust site / GP surgery site / any private clinics - search for Register of Interests, and search within, if not publicly available need to write to NHS Trust using FOI if necessary

NICE/SIGN search for 1) Firstname Lastname 2) if present, search for DOI

PubMed Firstname Lastname - search for declaration of interest

Linkedin

Companies House - UK registration of all limited companies

Electoral Commission - personal or company donations

Patents (Google Patents - patents.google.com)

LexisNexis (any mentions of companies / sponsors)

TV / Radio (transcripts accessible via Box of Broadcasts)

PubMed: Journal articles in last 5 years, conflicts of interest declarations

ClinicalTrials

OpenPayments.gov

Youtube lectures for any declarations of interest made

Appendix 2

Methods of recruiting lay people, patients, citizens and professionals to participation

1) Citizens - invited via social media and citizen groups including Sense about Science network
2) Patient activists - recruited via National Voices, Sling the Mesh, Patients Association UK
3) Healthcare students - via Medical Schools Advisory Council, nursing, midwifery, and paramedical school associations via social media, and via ELAG (Educational Leads Advisory Group) to contact medical students, medical school newsletters.
4) Healthcare professionals working independently eg consultants, pharmacists, independent nurse consultants/prescribers — via social media, medical press, medical school newsletters
5) Early career researchers - training forums, Colleges via social media.

Appendix 3

Vignettes used in online questionnaire

A

You have developed a problem with your hip. You go to see a doctor, who you have known for several years and they have always given good care. They are qualified to do their job.

The doctor recommends an operation in the NHS to replace your hip.

This is a type of hip replacement called Mars. You are told that this is the standard type of hip replacement used for people like you and usually has very good results.

The doctor tells you that they do not have any shares or patents in Mars, nor do they get paid extra to fit this type of hip replacement.

B
You have developed a problem with your knee. You go to see a doctor, who you have known for several years and they have always given good care. They are qualified to do their job.

The doctor recommends an operation in the NHS to replace your knee.

This is a type of knee replacement called Jupiter. You are told that this is the standard type of knee replacement that is used for people like you and usually has very good results.

The doctor tells you that they do not have any shares or patents in Jupiter. However the doctor says that they have been to a conference about this type of knee replacement. The company paid for them to travel to a conference in Europe and for a course about it, which would normally have cost a few thousand pounds.

C
You have developed a different problem with your other hip. You go to see a doctor, who you have known or several years and they have always given good care. They are qualified to do their job.

The doctor recommends an operation in the NHS to replace your hip with a type of hip replacement called Venus.

You are told that this is the standard type of hip replacement for people like you and usually has very good results.

The doctor tells you that they are part of the team that developed Venus hip replacements. The doctor works as a consultant for the company that makes them and travels to conferences to explain the hip replacement to other doctors. The doctor is a part-owner of the patent for Venus hip replacement and has shares in the company making them.

D

You have developed a different problem with your other knee. You go to see a doctor, who you have known or several years and they have always given good care. They are qualified to do their job.

The doctor recommends an operation in the NHS to replace your knee.

You are recommended a type of knee replacement called Saturn. You are told that this is the standard type of knee replacement used for people like you and usually has very good results.

You do a search online. You find that this doctor owns a patent and shares in Saturn. Last year the doctor received £50,000 for working as a consultant for the company.

Appendix 4

Criteria included in 'gold standard'

As many participants listed potential conflicts as items which were out of scope of ICMJE criteria/ NHSE criteria some were dismissed; for example:

Director of company dissolved >5 years ago
Single talk given to public >3 years ago
Public service job > 5 years ago
Honorary Fellow/Member of medical organisation
Examiner for a Royal College exam (reasonably supposed to be part of a job already declared)

Appendix 5

Estimation of how many forms filled in per year to declare interests by healthcare professionals

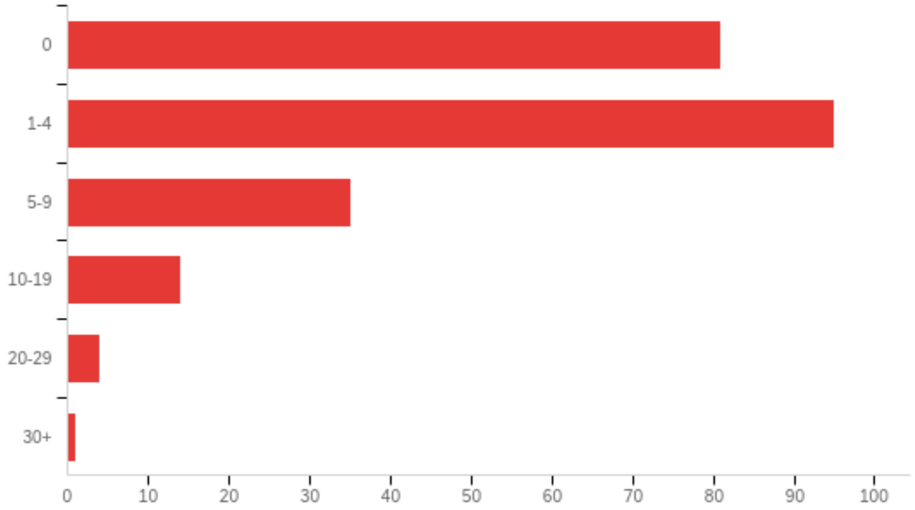

Appendix 6

Relationship of Trust to increasing conflicted scenarios

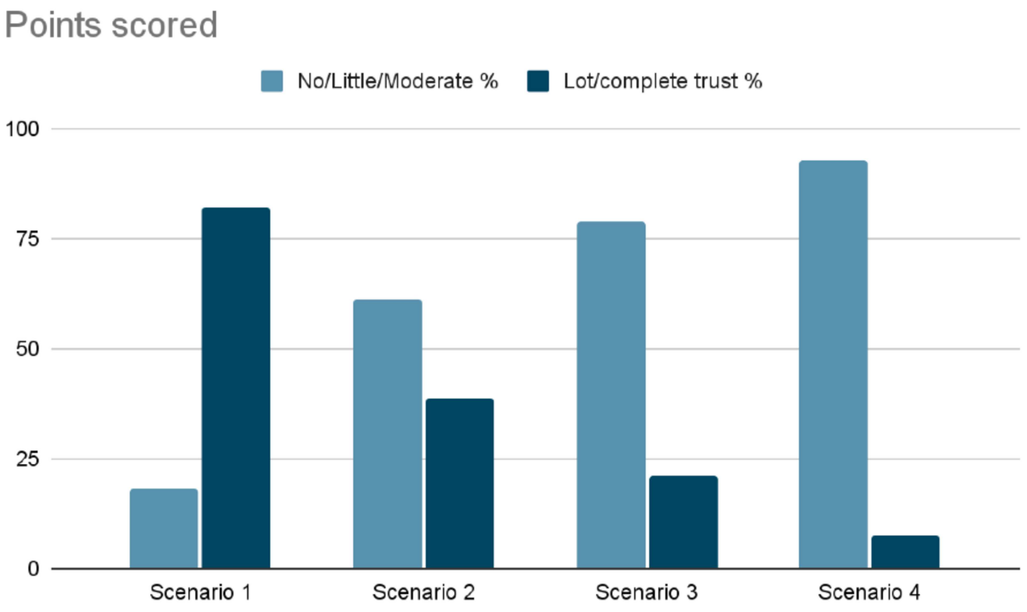

A	B	C	D	E
	No/Little/Mode	Lot/Complete trust		
Scenario 1	18.1%(64)	81.9% (289)		
Scenario 2	61.2% (211)	38.8%(134)		
Scenario 3	79% (270)	21.1% (72)		
Scenario 4	92.7% (315)	7.4% (25)		

Appendix 7

Relationship of belief doctor acting in patients best interest to increasing conflicted scenarios

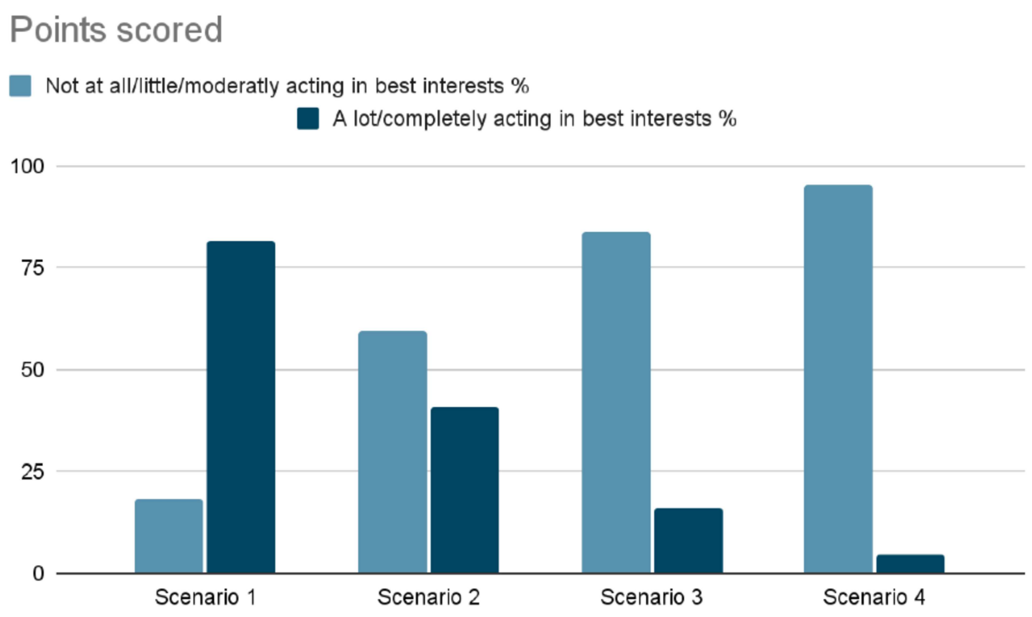

Scenario 1	18.5(65)	81.6 (287)
Scenario 2	59.3(204)	40.7 (140)
Scenario 3	83.7(288)	16.3(56)
Scenario 4	4.7 (16)	95.3 (324)

Appendix 8

Results - participants locating fit for purpose and gold standard declarations

Key:

'Links located" - total number of links reported by research participants

"Number fit for purpose" - weblinks containing a formal declaration of interests

"Participants reporting any results" - any information obtained was included

"Participants reporting a fit for purpose link" - number of participants finding a 'fit for purpose' link

"Significant entries%" - all significant entries including financial entries

Responses finding all categories - at least one entry from each category

Professiona	Links located	Number fit for purpose	Participants reporting any results	Participants finding a fit for purpose link	Average% significant interests found	Total responses containing each category of declaration
A	11	3/11	14/24	2	64%	8/14
B	14	3/14	12/24	6	57%	8/12
C	5	1/5	10/22	1	30%	3/10
D	6	5/6	10/21	5	90%	9/10
E	10	1/10	9/23	3	67%	6/9
F	9	6/9	14/24	8	79%	11/14
G	19	2/19	17/22	3	18%	3/17
H	17	3/17	15/22	2	20%	3/15
I	3	2/3	11/23	4	55%	6/11
J	8	2/8	16/23	10	75%	12/16
K	12	1/12	10/22	2	70%	7/10
L	12	4/12	16/25	11	100%	16/16
M	10	2/10	13/22	2	15%	2/13

Appendix 9

Additional qualitative data from interviews arranged by theme

Theme 1: COIs in healthcare can be difficult to define

"It means that either directly or indirectly, a health care professional stands to benefit from a decision that is made about the appropriate treatment for a patient". (P16_F60-69_LTC)
"It means to me, that a professional may have a financial interest in a firm which is manufacturin drugs or medical devices and may or may not declare that interest to the people that they are ca for or prescribing for". (P21_F60-69_Lay)
"It could be having a bit of a conference with a dinner or a few drinks or giving a few products fr They think it's their right" (P14_F50-19_Lay)
"you can have a conflict between the need for public health …. and the needs to provide service the person in front of you because it's an opportunity cost. … it's balancing out …. the exigent circumstances of the person in front of you or the group you're talking about it one time." (P16_F60-69_LTC)
**"*It could be not direct to them. It might be a contribution towards research to which they have ar interest. So, they think it's acceptable because it's going towards research, but it's clearly swayi the opinion and the reason behind things".* (*P14_F50-19_Lay*)**
"I think people struggle with understanding what a conflict of interest is because you could hav interest in something and not have any financial gain from it and not declare that interest …. I th on the whole, a lot of people don't declare interests because they don't really understand what th interests are and how they conflict". (P10_M60-69_Activist)
"Well, at the extreme end, it means doctors being involved with the direct purchase of equipme drugs that they have an interest in that company, so they therefore are likely to get greater divid back. Or if they're actually part owners of companies, … they're getting funds directly from company either through profits or when the company is sold. So that's a very strong confli interest". (P17_M60-69_Activist)
"one of the areas I thought of was where your beliefs, your beliefs will influence what you do. An think that that we don't look at that an awful lot. … So I'd be very, very careful that my unconscio bias doesn't conflict with professionalism, and I think that that's a really difficult area because y talking about thoughts, you know, rather than financial interests and sometimes you know that c be more insidious than, you know, money". (P10_M60-69_Activist)
"So, I actually think healthcare professionals don't understand what conflict of interest means. I think it's that fundamental. They don't understand biases in science. They don't understand thei need for not only reflection, but reflexivity. That they need to reflect on their own values and beli in what they're bringing to the evidence that they're presenting to patients". (P7_F50-59_Activist

“It's trust. This is what I said about bribery and all that type of thing. It's very sad because if you' the health profession, I always feel that these people should be within the heart, caring. It's all ab being a caring person.” (P14_F5-59_Lay)
“Personally, unfortunately, I've lost all trust of all items due to, I feel, a conflict of interest, happened to me. I always research everything. In fact, I refuse it now, anything. To a point you total trust with everything, and it can ruin people's trust in things, in medication, so it's (P14_F5-59_Lay)
“*I know very little about it…. It makes me think of when drug companies go round, and they do a lunc the doctors and they do a presentation about their product and the doctors prescribe their product. But it's much wider and there's lot of other circumstances…I don’t really know much about them”. (P1, lay)*
“*It's almost like a bribe, possibly, from the pharmaceutical… It could be a soft one, where maybe the me doesn’t recognise that it's happening, or it could be something that’s really quite obvious but they thin acceptable because there's no law there.” (P14, lay)*
“*...it’s to do with the power imbalance. You have gone to this professional for help because you don’t much about what is happening to you. So, it’s about trust… you trust them to do the right thing for because you don’t know how to do it yourself.” (P5, activist)*

Theme 2: COIs in healthcare can be hard to find

“...if a new product is brought to the market, then, understandably, if it’s going to be used it nee be promoted, so that’s understandable. But I don’t think it should be too difficult to have a syste transparency around that.” (P1_F40-49_Lay)
“I think that the openness and transparency should apply equally to both financial and non-finar and I don’t think… I suppose, financial, you assume is worse, if you like, or more likely to be n my best interest, but I think, actually, both equally need to be clear as to what’s happen (P5_F60-69_Activist)
“ … so in terms of conflict of interest, I don't think it's necessary for the patient to do a huge am more. I think the NHS is aware of the issue and they've produced reports and whatever on the subject in past years, I believe. For me, the thing that's needed, and you know, I'm not sure how much this is in place, is the transparency and whistleblowing and ability to contest after the eve …” (P13_M70-70_Lay)

Theme 3: COIs present both challenges and benefits for patients

" … they got their paid research grants, and they were thinking well we could do this, or we cou that, but now, I look back, I think that wasn't really in the patient's interests. Because the patier bamboozled with choices that she knew nothing about. … And you were still trusting implicitl they had your best interests at heart when I think they were looking at interesting tr opportunities for medical students. … I used to have a career and a nice home. I've lost everyth this undisclosed bias in favour of polypropylene implants." (P12_F60-69_Lay)
"I think when there are conflicts of interest, there's potential for corruption. Potential for corrupti always a problem... It depends on the rules that you have in place that stop corruption. … For me depends on what becomes from the norm and acceptable. … And in some of these conflicts of interest issues patients are moved into a commercial transaction where the distinction between diagnosis and treatment and the offer is being blurred. And you're then into being sold a product which you might not completely need." (P19_F60-69_Lay)
"i's that complexity because it's about, you know, not assuming that the conflict of interest is a b thing for everybody as well. Sometimes. Well, actually that might be good thing, cause I believe t and I want somebody who also believes that." (P15_F50-59_Carer)
"*I don't have any huge problem. And I think my general view of health is that the right provider is some more important than keeping it in house, and you know the NHS tends to keep things in house rathe use it outside, but that's nothing, that profit in itself is not a not a dirty word…" (P13, lay)*
"*Because it can affect outcomes for people's care. There's been incidences of, for example, poor d being employed, hip replacement joints, I think there was a bad set of them at one point. One assume they were being promoted by the company at the time. The vaginal mesh incidence, again, one as across the gynaecological board, they were being hyped as the best thing. And that has ruined people's in fact, it's killed some people." (P9, lay)*

Theme 4: COIs need to be carefully managed

"So if I was looking for a private treatment for something that I might well go and look, you know there wasn't an everyday, not like a dentist or something like that. But I might then go and look a person, that individual and sort of look at at what they do and why they do it. And you can often that, you know, things that come up on Facebook and places. There are clear links between." (P15_F50-59_Carer)
"I think it's much more complex issue than that as we're dealing with human beings and it's reall hard to unpick. And I think you have to go, I guess what you have to do is you have to go with th worst case scenario that somebody who's got… You know, some kind of major conflict of interes abuse that and therefore you have to put such systems and structures in place that avoid that w case scenario." (P15_F50-59_Carer)

“I don’t think I would have known that you would find that…and so I suspect that if I don’t know [illegible] have got a background in the pharmaceutical industry, and medical friends and so on, I [illegible] the layperson would have very little idea that that was there to be found. So, I’m not [illegible] expecting people to go and search online would be the best way to manage it. I think, probably, [illegible] have to be even more upfront and declare it at the consultation.” (P6_F60-69_Lay)
“Actually, I’m not even given the name of consultants a lot of the time if I go to hospital, but [illegible] seeing a consultant, all their interests should be there. I shouldn’t have to go looking for it. It [illegible] be very easy to find” (P21_F60-69_Lay)

“I think if somebody declared something, they can always declare part of the truth, because the [illegible] person who’s declaring can choose. They can choose how they phrase it. They can leave things [illegible] So, to me, it seems pointless.” (P2_F50-59_Lay)
“But my problem with the whole setting up of a registering of interest, is the fact that it’s [illegible] Most patients won’t ever question this. They will never question their doctors. So, however [illegible] information there is, however available and easy you make it to find, people aren’t going to [illegible] because I trust it. I trusted the medical establishment before I started working for [illegible] organisation), and now I don’t. I really don’t.” (P2_F50-59_Lay)
“that doesn't mean somebody's not a good person and they're not doing the right thing…you [illegible] really believes in all the stuff…But he must be biased by that. Is that good bias? Is that bad [illegible] don't know, you know? But actually, if you saw him as a clinician, you should probably [illegible] (P15_F50-59_Carer)
“I think that we probably need to ensure that, particularly people who are responsible for those decisions, need to be trained well in understanding what conflicts of interests are. They need [illegible] training, regular updates, and I think that they need to be challenged on a regular basis. … I [illegible] every year you probably need to go through a conflict of interest, you know, in your work. And I [illegible] say that it would be better to do it with someone, rather than by yourself” (P10_M60-69_activist)

“… most companies, you know, outside of the health care, they have to declare their interests [illegible] you can find out what their interests are by looking at them on the Internet. Health is actually [illegible] those areas, it's very, very grey and shady and not saying that it's bad, it's grey. We haven't [illegible] done an awful lot around health and care.” (P10_M60-69_activist)

“the mandatory things are problem in some ways, because people will find a way around it ar they'll find a way around it if it's not compulsory as well. Although the issue about it bein compulsory is if you can get the message out there and somebody hasn't.” (P15_F50-59_Carer)
“… public members can then raise concerns to the board of the RMC or their BMA, or the ICN and say you know this, this drug which we're using is seen in like a 70 to 80% uptake or an increase in prescri Is this something normal?” (P11, lay)
I’m aware that, for example, if I were to read an article in, say, the British Medical Journal, which I do very often, but that professionals are required, or writers are required, to declare any interests that they So that the readers can make a judgement about whether or not that interest has influenced their resea writing. (P21,lay)
