# Supplementary material for: ‘You feel like you’ve been duped’: is the current system for health professionals declaring potential conflicts of interest in the UK fit for purpose? A mixed methods study
Source: BMJ Open. 2023 Jul 26;13(7):e072996. doi: 10.1136/bmjopen-2023-072996 (PMC10373698; doi:10.1136/bmjopen-2023-072996)
Supplement: Author's manuscript [file bmjopen-2023-072996.draft_revisions.pdf]

BMJ Open

BMJ Open is committed to open peer review. As part of this commitment we make the peer review history of every article we publish publicly available.

When an article is published we post the peer reviewers' comments and the authors' responses online. We also post the versions of the paper that were used during peer review. These are the versions that the peer review comments apply to.

The versions of the paper that follow are the versions that were submitted during the peer review process. They are not the versions of record or the final published versions. They should not be cited or distributed as the published version of this manuscript.

BMJ Open is an open access journal and the full, final, typeset and author-corrected version of record of the manuscript is available on our site with no access controls, subscription charges or pay-per-view fees (http://bmjopen.bmj.com).

If you have any questions on BMJ Open's open peer review process please email info.bmjopen@bmj.com

BMJ Open

You feel like you've been duped". Is the current system for health professionals declaring potential conflicts of interest fit for purpose? A mixed methods study.

Journal:	*BMJ Open*
Manuscript ID	bmjopen-2023-072996
Article Type:	Original research
Date Submitted by the Author:	23-Feb-2023

Complete List of Authors:	McCartney, Margaret; University of St Andrews,<br>Metsis, Katrin; University of St Andrews, School of Medicine<br>MacDonald, Ronald; University of St Andrews School of Medicine, ; University of Dundee School of Medicine,<br>Sullivan, Frank; University of St. Andrews, ; North York General hospital,<br><br>Ozakinci, Gozde; University of St Andrews<br>Boylan, Anne-Marie; Oxford University, Nuffield Department of Primary Care Health Sciences

Keywords:	MEDICAL ETHICS, Information management < BIOTECHNOLOGY & BIOINFORMATICS, HEALTH SERVICES ADMINISTRATION & MANAGEMENT, Health policy < HEALTH SERVICES ADMINISTRATION & MANAGEMENT, Protocols & guidelines < HEALTH SERVICES ADMINISTRATION & MANAGEMENT, Patient-Centered Care

SCHOLARONE™
Manuscripts

I, the Submitting Author has the right to grant and does grant on behalf of all authors of the Work (as defined in the below author licence), an exclusive licence and/or a non-exclusive licence for contributions from authors who are: i) UK Crown employees; ii) where BMJ has agreed a CC-BY licence shall apply, and/or iii) in accordance with the terms applicable for US Federal Government officers or employees acting as part of their official duties; on a worldwide, perpetual, irrevocable, royalty-free basis to BMJ Publishing Group Ltd ("BMJ") its licensees and where the relevant Journal is co-owned by BMJ to the co-owners of the Journal, to publish the Work in this journal and any other BMJ products and to exploit all rights, as set out in our licence.

The Submitting Author accepts and understands that any supply made under these terms is made by BMJ to the Submitting Author unless you are acting as an employee on behalf of your employer or a postgraduate student of an affiliated institution which is paying any applicable article publishing charge ("APC") for Open Access articles. Where the Submitting Author wishes to make the Work available on an Open Access basis (and intends to pay the relevant APC), the terms of reuse of such Open Access shall be governed by a Creative Commons licence – details of these licences and which Creative Commons licence will apply to this Work are set out in our licence referred to above.

Other than as permitted in any relevant BMJ Author's Self Archiving Policies, I confirm this Work has not been accepted for publication elsewhere, is not being considered for publication elsewhere and does not duplicate material already published. I confirm all authors consent to publication of this Work and authorise the granting of this licence.

For peer review only

You feel like you've been duped". Is the current system for health professionals declaring potential conflicts of interest fit for purpose? A mixed methods study.

Margaret McCartney, Honorary Senior Lecturer* (1), Katrin Metsis, Research Fellow (1),
Ronald MacDonald, medical student (1), Frank Sullivan, Director of Research (1),
Gozde Ozakinci, Professor of Health Psychology (2), Anne-Marie Boylan, Senior Research Fellow (3)

1) School of Medicine, University of St Andrews, North Haugh, St Andrews, Fife, UK KY16 9TF 2) University of Stirling, Stirling, UK FK9 4LA 3) Nuffield Department of Primary Care Health Sciences, Oxford University, OX2 6GG.

*Corresponding author
mm494@st-andrews.ac.uk

For peer review only

Abstract:

Objective: To understand: if professionals, citizens and patients can locate UK healthcare professionals' statements of declarations of interests, and what citizens understand by these.

Design: The study sample included two groups of participants in three phases. First, healthcare professionals working in the public domain (health professional participants, HPP) were invited to participate. Their conflicts and declarations of interest were searched for in publicly available data, which the HPP checked and confirmed as the 'gold standard'. In the second phase, laypeople, other healthcare professionals and healthcare students were invited to complete three online tasks. The first task was a questionnaire about their own demographics. The second task was questions about doctors conflicts of interest in clinical vignette scenarios. The third task was a request for each participant to locate and describe the declarations of interest of one of the named healthcare professionals identified in the first phase, randomly assigned. At the end of this task, all lay participants were asked to indicate willingness to be interviewed at a later date. In the third phase, each lay respondent who was willing to be contacted was invited to a qualitative interview to obtain their views on the conflicts and declaration of interest they found and their meaning.

Setting: Online, based in the UK.

Participants: 13 public-facing health professionals, 379 participants (healthcare professionals, students and laypeople), 21 lay interviewees.

Outcome measures: 1) Participants' level of trust in professionals with variable conflicts of interest, as expressed in vignettes, 2) participants' ability to locate the declarations of interest of a given well-known healthcare professional, 3) laypeople's understanding of healthcare professionals declarations and conflicts of interest.

Results: In the first phase, 13 health professionals (HPP) participated and agreed a 'gold standard' of their declarations. In the second phase, 379 citizens, patients, other healthcare professionals and students participated. Not all completed all aspects of the research. 85% of participants thought that knowing about professional declarations was definitely or probably important, but 76.8% were not confident they had found all relevant information after searching. As conflicts of interest increased in the vignettes, participants trusted doctors less. Least trust was associated with doctors who had not disclosed their conflicts of interest. 297 participants agreed to search for HPP 'gold standard' declaration of interest, and 169 reported some data. Of

those reporting any findings, 61 (36%) located a relevant link to some information deemed fit for purpose, and 5 (3%) of participants found all the information contained in the ‘gold standard’. In the third phase, qualitative interviews with 21 participants highlighted the importance of transparency but raised serious concerns about how useful declarations were in their current format, and whether they could improve patient care. Unintended consequences, such as the burden for patients and professionals to use declarations were identified, with participants additionally expressing concerns about professional bias and a lack of insight over conflicts. Suggestions for improvements included better regulation and organisation, but also second opinions and independent advice where conflicts of interest were suspected.

Conclusion: Declarations of interest are important and conflicts of interest concern patients and professionals, particularly in regard to trust in decision making. If declarations, as currently made, are intended to improve transparency, they do not achieve this, due to difficulties in locating and interpreting them. Unintended consequences may arise if transparency alone is assumed to provide management of conflicts. Increased trust resulting from transparency may be misplaced, given the evidence on the hazards associated with conflicts of interest. Clarity about the purposes of transparency is required. Future policies may be more successful if focussed on reducing the potential for negative impacts of conflicts of interest, rather than relying on individuals to locate declarations and interpret them.

Registration: The protocol was pre-registered at https://osf.io/e7gtq.

Patient involvement: We thank the lay group for their advice before, during and after the study. They helped to design the methods, the questionnaires, the choice of professional participants, and assisted in recruiting lay participants.

Ethics: This project was approved by the University of St Andrews School of Medicine Ethics Committee, MD16045.

Funding: Supported by a studentship grant for RM and a research grant supporting KM qualitative interviewing from the University of St Andrews Research Fund.

Declaration of interest: MM has been paid for writing/broadcasting in relation to issues connected with campaigning around conflicts of interest. All authors have completed the Unified Competing Interest form (available on request from the corresponding author) and have no other declarations to make regarding support from any organisation for the submitted work; financial relationships with any organisations that might have an interest in the submitted work in the previous three years.

What is already known on this topic	Conflicts of interest are associated with more expensive, poorer quality healthcare<br><br>Guidelines have attempted to standardise declarations of interest from healthcare professionals<br><br>The impact of declarations is uncertain
What this study adds	Current efforts to produce transparency on conflicts of interest are failing<br><br>Patients want to know about healthcare professionals conflicts of interest; trust more when there are fewer conflicts, and least when they are undisclosed<br><br>Better transparency may result in unintended consequences, including misplaced trust and work for patients

For peer review only

Introduction:

Contention has surrounded the definition, declaration, and management of interests in medical practice for decades. When the US Congress asked 'Is science for sale?' in a series of hearings regarding research fraud in the late 1990s, declarations of interest were generally haphazard and voluntary. In 2009, The International Committee of Medical Journal Editors produced a standardised template for recording and publishing declarations, which has been serially upgraded since (1). However, declaring interests has subsequently become an expectation in routine clinical practice, not just academic publishing, and is now law via Sunshine Acts for healthcare professionals in several countries. In NHS England, guidance published in 2017 stipulates how and which declarations should be publicly made (2).

There is evidence of widespread harm to patients and healthcare systems because of financial conflicts of interest. For example, guidelines dealing with opioid prescribing for non-cancer pain (recognised as driving 'the opioid crisis') have had a "pervasive presence" of influence of the pharmaceutical industry amongst guideline authors or organisations receiving funding from them (3). Doctors with financial conflicts of interest are more likely to have favourable views on side effects from medications (4). Meanwhile, doctors receiving gifts and education from pharmaceutical sales representatives have poorer quality prescribing, and believe their peers, but not themselves, are adversely affected by conflicts (5).

Healthcare professionals are often asked to make declarations, or "declarations of interest" (DOI) in different venues, for example, workplaces, academic journals, and conference presentations. Such declarations relate to the act of recording any interests which may be, or could potentially become a conflict, depending on context or circumstances. A conflict of interest, however, is "a set of circumstances that create a risk that an individual's ability to apply judgement or act in one role is, or could be, impaired or influenced by a secondary interest." (6). The UK government commissioned the Independent Medicines and Medical Devices Safety Review (IMMDS) in 2020, to investigate how the health service responded to concerns about medical products, and one conclusion was that serious problems are caused by a lack of transparency of conflicts of interest (COI) (7). However, there may be uncertainty over when a declaration represents a conflict, particularly when a declaration is prepared in advance and used for multiple purposes. The review recommended that doctors should make statutory disclosures of interests on a central register, including, for example, consultancy payments from pharmaceutical companies, patents for technological devices, or shareholdings in device companies. The General Medical Council already recommends that doctors are open and transparent about their interests. Previous inquiries, for example, the Health Select Committee in the 2005 report 'The Influence of the Pharmaceutical Industry', recommended that all healthcare professionals make statutory declarations of interest to their regulator (8), but this did not occur.

There are multiple types of potential conflicts of interest. NHS England classes these as financial or non-financial. The first is where an individual receives or may receive a direct financial benefit from the consequences of the awarding of funding (2). This can be direct (for example, a shareholder receiving more funds for increased sales in a product) or indirect (where a person closely associated, such as a spouse or business partner will benefit similarly) Non-financial interests can be professional (e.g. a decision likely to enhance a career or status, or an intellectual bias) or non-professional (where other interests, such being a member of a lobby group, may compete).

Many countries mandate disclosures of interest by health professionals (9), The US Sunshine Act, which mandates transparency of payments from industry, was implemented in 2013. The same year, a Disclosure Code

For peer review only

by the European Federation of Pharmaceutical Industries and Associations (EFPIA) was implemented to improve transparency, but is variable by country where it conflicts with other laws (10). In the US, the Act does not seem to have resulted in improved patient knowledge of physician payments, with less than 5% of people knowing whether their own doctor has received industry payments (11). Declarations of interest may be intended to direct their management, for example, in guideline committees (12); regulators may also stipulate specific conflicts which must be declared to patients (13). This has also been emphasised in the IMMDS review, which has called for patients to be able to access declarations in order to “reach informed decisions about who is best to treat us” (7). There are therefore multiple purposes to which declarations can be put.

Disclosures of interest have been historically acknowledged as necessary but also with multiple inadequacies in practice, including poor quality recording and subsequent management (14). There are additional concerns that statutory declarations, rather than reducing the negative impact of conflicts of interest, may transfer the burden of searching for, interpreting, and acting on them to patients (15). Further, there are concerns that disclosing interests may increase bias through ‘moral licence’, when doctors believe that disclosure equals management of conflicts, and where patients and citizens believe that transparency negates bias (16). In the US, patients who are explicitly told about a doctor’s financial conflicts with industry do not appear to change their attendance with the doctor, with no loss of trust (17); however other research suggests that disclosure may result in an increase in trust if it is regarded as a sign of honesty (18). Patients at US cancer centres have low levels of concern about doctors' financial conflicts from pharmaceutical companies (19); however, this study did not ask patients what they thought the negative impacts might be. The UK has a voluntary system, Disclosure UK, where payments to professionals from pharmaceutical companies are published annually, but most money is undeclared (20). NHS Trusts in England mandate disclosures for staff but these are incomplete and of poor quality (21). Therefore, while voluntary and mandated disclosures from healthcare staff are available to patients in the UK, these are problematic due to their quality, and it is unknown whether these incur unintended hazards from disclosures, and whether they enable evidence-informed decision making.

As the NHS responds to the Cumberlege report (22), potential actions in the recording and managing of declarations of interest require consideration. Little is known about how much knowledge patients, citizens, professionals and policymakers have regarding conflicts of interests among healthcare professionals, or locating and interpreting this information. A 2016 systematic review on knowledge, beliefs and attitudes of patients and the public towards interactions between professionals and the pharmaceutical and device industry found low levels of concern about financial conflicts but did not include any studies from the UK or Europe, with the exception of Turkey (23).

There is a large gap in our understanding of the most effective way to declare and interpret interests, and what patients, citizens and professionals think about the impact of them. This is required in order to ensure that any change in policies are beneficial and meet their intended purpose. The aim of the study was to understand: if professionals, citizens and patients can locate UK healthcare professionals’ statements of declarations of interests, and what citizens understand by these.

Methods:

This research takes a pragmatist perspective (24, 25). While the different epistemological standpoints of qualitative and quantitative methodologies are acknowledged, a mixed methods approach was chosen to gain a comprehensive understanding of the citizen and patient participants’ perspectives of COIs in health professionals. The online survey, completed in the second phase, provides a quantified understanding of the respondents’ perceptions of health professionals’ variable interests and the process of locating declarations. Qualitative interviews in the third phase investigates lay perspectives of declarations and conflicts of interest in healthcare, and current declaration strategies. Thus, quantitative and qualitative data are used to gain an understanding of the different aspects of the phenomenon, which are integrated but keeping their epistemological differences (26, 27, 28).

Phase 1: Methods development

For peer review only - http://bmjopen.bmj.com/site/about/guidelines.xhtml

The research team established a PPI group. This consisted of 4 lay people who are involved with patient representation at the UK Royal College of General Practitioners or who have been involved in lay activism regarding surgical mesh. The PPI group helped design the methods, the questionnaires, the choice of professional participants, and assisted in recruiting lay participants.

Professionals (HPP, Health Professional Participants) were identified and invited on the basis of their recent history of giving medical advice or information to the public, where a citizen might reasonably wish to know their declarations of interest. A mixture of healthcare officials based in the UK (for example, doctors employed by government), heads of royal colleges/professional societies, pressure groups, NICE and SIGN guideline authors, and doctor journalists were invited. This was done in batches of 10-15 aiming to recruit 12-15 in total. A standard process was used to search for the professionals' conflicts of interest (Appendix 1). Each professional was asked to check the findings and approve or disagree with them. This formed the 'gold standard'. Statements within each 'gold standard' declaration were divided into 'financial professional', 'non-financial professional' 'personal' and 'indirect' as per the NHS England disclosure framework (2). This recruitment of healthcare professional participants (HPP) completed the first phase of the study.

Phase 2: Online survey

An online survey (using Qualtrics) was developed and tested with the lay group (supplement). This contained 4 scenarios explaining and asking opinions on potential financial conflicts of interest. We described these to participants as 'interests' and not 'conflicts'. A wide range of participants from citizen, patient, activist, professional and student groups were formally invited (Appendix 2) to participate online, with patient and professional networks asked to publicise the research via social media. The invitation contained a web link to the survey which prospective participants could click on. Demographic and occupational information was requested. Healthcare professionals were additionally asked how many declaration of interest forms they were asked to complete a year. Participants were then asked to express their trust in a doctor, and whether they felt they were acting in their best interests, within a vignette about a surgical procedure, where doctors making a recommendation had variable but increasing financial interests. These were either not present, present and declared, or not declared but found by the participant. This was designed to give information about what a declaration of interest is, to gauge views on their importance in terms of impact on personal decision making, and to explain and prime participants as to what a declaration of interest was before being asked to locate them (Appendix 3). These used 5 point Likert scales.

The participants were then randomly assigned to one HPP from the group of 13, with a brief introduction about the person, and asked to spend around 10 minutes (as suggested by the PPI group) to search for and report their declarations of interest. However, participants could spend as little or as much time as wished. We explained that we were not seeking any 'personal' information such as age or marital status or address but either a statement of declarations of interests, or the interests they found. Information on how long participants felt reasonable to search for a conflict was also sought.

They were asked to report findings in a web form. Excel was used to tabulate each piece of data reported by participants. These were compared to the professional's 'gold standard', which were divided into financial and non-financial declarations. Each was deemed 'significant' or 'non significant' (Appendix 4) based on NHS England criteria (2).

Participants' responses to the HPP 'gold standard' were analysed by hand. Participants were asked to record all information found, supplying relevant web links. The responses were recorded and assessed for

For peer review only

accuracy and completeness via comparison with the 'gold standard' (MM, RM). Given the difficulty the task was expected to pose, marking was generous. We did not ask either the professionals or the participants to categorise interests within each NHS England category of declarations (e.g. financial, non-financial professional, non-financial personal interests, and indirect interests) but the research team categorised and marked them on this basis. If a 'gold standard' declaration containing full details was not available online for participants to locate, locating a single declaration from each category of declaration was marked as successful, even if incomplete. 10% of the results were checked by the second researcher for accuracy and no disagreements were found. Null declarations were excluded in the tally (ie the denominator was according to the declarations present). This strategy would have overestimated the tally of declarations found, by design. At the end of the survey, all lay people were asked if they would like to take part in a qualitative interview at a later stage.

Third phase: interviews

In the third phase, all lay participants who stated they were willing to take part in an interview were contacted. This was a semi-structured, qualitative interview where their views about both declarations of interest and where these represented conflicts could be explored. 50 agreed to be contacted and of these, 21 consented to be interviewed (KM, MM) (Appendix Table 1). A semi-structured interview format was chosen as this method is well suited for the exploration of opinions and perceptions, enabling the further exploration of the topics identified by respondents (29). A topic guide was developed for use in the interviews with the understanding that it would be iteratively refined and new questions added as data collection progressed. Questions included exploring what declarations of interest were, and their purpose, what a 'conflict of interest' in healthcare professionals means, what was known about them, if they were perceived as important, and how they should be managed. The initial topics were developed after discussion with the lay group and related to real-life practice in the UK, where the medical regulator advises that professionals should use self-judgement to decide when a conflict occurs and when it should be declared (13). All interviews were transcribed verbatim and transcripts were transferred to NVivo for analysis.

Interview data was analysed using thematic analysis (30). KM completed 20 out of 21 interviews (MM did one). KM created the initial set of codes based on six transcripts. MM read and discussed all transcripts, interview notes, and initial codes. This was then discussed with AMB alongside, with conceptualisation of potential themes. Next, all transcripts were coded in NVivo and initial themes were developed by KM. Initial themes were further developed and refined with input from MM and AMB. KM, MM and AMB developed the final three themes and these were agreed by the whole team.

Results:

Phase 1:

A total of 65 professionals were invited; 3 sent a formal declination and 13 consented to take part. Two did not respond after further information was requested and sent; the remainder did not reply. The research team completed the 'gold standard' and presented it to the HPP. All but one statement was agreed for one HPP. After discussion it was deemed inaccurate by both the professional involved and the research team. No information contained within the 'gold standards' created was not available online.

The professional participants' declarations varied markedly, with some having minimal or only professional declarations to make (minimum 6, including job roles) and others having multiple financial declarations (maximum 20, e.g., sponsorship, consultancy, shares, private practice, patents, multiple professional roles). Within NHS England categories of declarations (financial, non-financial professional, non-financial personal, and indirect) some participants had multiple in one category and others were blank.

Phase 2:

For peer review only

Survey findings

In the second phase, 378 individuals participated and answered at least some of the questions. 266 (70%) were female and the vast majority lived in the UK (354, 93%). 141 (37%) described themselves as lay (citizens/patients), and the remainder as either training, trained or working in healthcare. The median age was 50-59 (range 18-80+). The majority of healthcare professionals participating were GPs (59, 25% of healthcare participants) followed by physicians and nurses. Of 230 healthcare professionals, 41% (95) filled in between 1-4 declaration of interest forms per year, with 35% (81) filling in none, and 23% (94) completing more than 5 (Appendix 5). A few participants did not complete all parts of the survey, meaning that some totals vary.

In the vignettes, when no interests were actively declared, 95% (335) of participants trusted the doctor either moderately, a lot, or completely and the same number felt they were acting in their best interests. If the doctor declared that they had been sponsored to travel to a conference by the company making a recommended joint replacement, the trust scored at moderately, a lot or completely fell to 79% (184) with 83% (286) judging as acting in their best interests. If the doctor declared heavier financial interests, including a patent and shares in the company, 54% (187) trusted the doctor moderately, a lot or completely, with a similar number (53%, 184) believing the doctor was acting in their best interests. When the doctor did not declare any interests, but was subsequently found by the participant to be acting as a consultant to the company, 22% (74) trusted the doctor moderately, a lot or completely, and 24% (80) felt they were acting in their best interests. There was a clear progression of decreased trust and decreased belief that the doctor was acting in their best interests with increasing financial interests. The least trust-generating scenario was where the conflict was not directly disclosed (Appendix 6, 7).

Survey respondents were then asked if they wished to continue to the task of searching for an individual's declarations of interest. 297 participants responded positively. 169 reported some data. 128 reported no findings. Each participants' findings were individually assessed to determine the proportion of declarations located within each type of category of interest, as described in the HPPs' 'gold standard' (Appendix 8). 61 found a source designed as a formal declaration of interest, for example, on guideline committee websites, which should have made it 'fit for purpose', containing at least some of the information from each of the four categories of NHS England guidance on declarations (financial professional and non profession, non-financial professional and non professional).

However, these formal, online declarations, despite being designed to be 'gold standard', were not all complete when compared to the standard we had generated with the HPP. 5 participants were able to replicate the 'gold standard' in their search. The top 5 highest cited links for the amount of accurate declarations were held in an organised register, such as NICE, parliamentary register, government website, or whopaysthisdoctor.org. When asked how long it should take to find a healthcare professional's declaration of interests, participants gave a range of 0-120 mins, mean 8.63 min.

Participants were asked how easy or difficult they found the search. Of 212 answering, 67.5% (143) said it was extremely or very difficult, and 36 (16.9%) somewhat or extremely easy. 76.8% (163) were not confident they had found all necessary information. 86% (184/213) said that finding declarations of interest were definitely or probably important.

Qualitative interview findings

For peer review only

At the end of the survey, all 50 lay people who consented were invited for an interview. All were individually contacted. 21 patients/citizens agreed to take part in this third phase and are referred to as they self-described. Thirteen were females and eight male; four described themselves as activists, and six as patients, with a variety of backgrounds including working in the pharmaceutical industry, research, or the public sector. The age range was 18-80, mean 62. Interviews lasted between 19-51 minutes and were transcribed verbatim for analysis. Participants could review their transcript. Nine requested them and three returned them with further comments; one corrected minor inaccuracies and the others added further comments.

Four themes were developed: *COIs in healthcare are difficult to define; Declarations of interest can be hard to find; COIs may present both challenges and benefits for patients; and COIs need to be carefully managed*.

Additional quotations are included in Appendix 9. Demographic information on participants is contained in Appendix Table 1.

COIs in healthcare can be difficult to define

Consistently, COIs were described as situations where care and treatment decisions benefitted the professional before the patient. However, although participants described a wide range and descriptions of COIs, they agreed that the concept and definition could be difficult to define. Some participants had acquired an understanding of COIs and declarations from different roles, including patient representative roles and experience of working in research or industry.

I know very little about it…. It makes me think of when drug companies go round, and they do a lunch for the doctors and they do a presentation about their product and the doctors prescribe their product. But I feel it's much wider and there's lot of other circumstances. I probably don't know much more other than a vague awareness that there are these other circumstances where there are potentially conflicts of interest. I don't really know much about them". (P1, patient)

Perceptions of a COI included a variety of financial COIs, particularly from industry. Examples of non-financial COIs given included gifts from pharmaceutical companies, HCPs holding powerful positions in decision-making bodies, involvement in research, considerations of reputation and career, or sponsorship from companies.

"… they might be offered honorarium or stipends, or a range of services, such as ghostwriters for medical journals, which will indirectly enhance or directly enhance their professional standing and from that flows their ability to garner research funding and have high profile research teams, so one can very directly benefit…even if the money isn't coming to you if it's coming to your research centre, it gives some people a lot of influence..." (P16, patient with long term condition)

Conflicts could also be caused by the way healthcare systems were organised and funded. Levers within the NHS could include GPs prescribing generically to save money, incentives to promote a product, or 'up-selling' on top of routine care. These could be regarded as a conflict, particularly when there was an uncertain justification.

"*do I need anti-glare on my glasses? I don't really know. But the lovely young person tells me that it's the best thing ever. So...the transaction becomes more complicated. Because there's a commercial element to the transaction that's being played out." (P19, lay, routine appointment for eyesight check at optician)*

For peer review only - http://bmjopen.bmj.com/site/about/guidelines.xhtml

Personal financial COIs were usually seen as more important, but participants emphasised how a broader, more pervasive impact could result from industry support.

"… they might be offered ..ghostwriters for medical journals, which will indirectly enhance or directly enhance their professional standing and from that flows their ability to garner research funding and have high profile research teams.. even if the money isn't coming to you if it's coming to your research centre, it gives some people a lot of influence over shaping the future direction of research or researchers and everybody else." (P16, *patient with long term condition*)

These conflicts could be unforeseen, and related to influence and power. For instance, professionals' involvement in research was a potential conflict, as interest in certain outcomes could lead to biased treatment recommendations.

"*Well, it means that the prescriber or the provider of the service is making decisions that aren't just in the interest of the patient or the user. That they may give too much priority to their own interests. Those might not be financial. They might be their research project, or something that they're supporting. But it's not putting the patient first, second, third and fourth, as it were." (P4, patient)*

Participants expressed concerns that professionals may lack insight into their conflicts or their potential impact, due to unclear definitions, the lack of oversight of COIs or professionals' unconscious bias. Participants thought that peer review, and training on how to recognise COIs could potentially help. Respondents also argued that personal beliefs may result in unconscious bias; intellectual bias was also viewed as damaging. One participant felt that HCPs do not understand the bias in sciences generally and therefore do not recognise the role of reflexivity when evidence is scrutinised to inform treatment decisions.

"*So, I actually think healthcare professionals don't understand what conflict of interest means…They don't understand biases in science. They don't understand their need for not only reflection, but reflexivity. That they need to reflect on their own values and beliefs in what they're bringing to the evidence that they're presenting to patients." (P7, patient activist)*

Declarations of interest in healthcare can be hard to find

While participants were not asked directly about trust and transparency, patient activists and representatives repeatedly expressed concern about how the difficulty finding declarations could lead to a loss of trust in the medical profession.

"*I think that in that last scenario, the reason why my trust diminishes is because of lack of transparency. I think that is why would you not tell someone?...Why would you not tell someone that you have a financial interest in this? So I suppose it's the discovery - you feel like you've been duped." (P10, activist)*

For some, transparency was a tool to navigate the vague nature of COIs, enabling patients to make decisions and reinforce the trust in HCPs. Equally, participants highlighted their desire for and expectation of trust in the medical profession. Undisclosed COIs could lead to potential or actual loss of trust, particularly when conflicts were undisclosed.

"I'd like it to be easier…. and I may not have found all of them." (P13, lay, patient representative roles)

COIs present both challenges and benefits for patients

None of the participants questioned the value of research. However contradictory aspects of COIs were raised by participants in relation to the interplay between industry and the profession. Some participants thought that industry funding for education might be worth accepting for their potential benefits. A small number (who also represented participants who had worked in the pharmaceutical industry) highlighted the positive role of industry/the private sector in providing education and training to trainees and HCPs. One participant described this as justified:

"yes, we need to have the declaration of a conflict of interest, but we actually have to allow a little bit of a conflict of interest for them to get to congresses and get educated…because if they don't, as I say, it's to our detriment ultimately, I believe…" (P17, activist, experience working in industry)

Others recognised the potential for industry sponsored education to be a potential conflict (professionals obtaining free education that would otherwise have to be paid for) and biased in nature, leading to poorer quality healthcare.

"...One assumes that they were being promoted by the company at the time. The vaginal mesh incidence, again, one assumes across the gynaecological board, they were being hyped as the best thing. And that has ruined people's lives, in fact, it's killed some people." (P9, lay)

Complexity was described. COIs (such as sponsored education) could lead to improved care, but the presence of a conflict could lead to an assumption that decisions were not in the best interest of the patient - whether or not they were. Two participants described suffering harm because of COIs around the treatment they received. Others had heard about such experiences or felt that their health needs were not always the primary consideration of the HCP because of conflicts. They discussed how COIs can lead to corruption and poor patient care by referring to the difficulties in defining and acknowledging COIs and the role of the trust in patient-HCP relationship.

"I like to start from the point that people are trustworthy and doing things from a good motive… There's a scenario where that doctor is somebody who genuinely wants to help patients, genuinely believes in the product, genuinely thinks that it's the right product for me, and happens to have received some payment for his role in developing it because his expertise has a value." (P1, patient)

COIs need to be carefully managed

Participants overall described COIs as challenging to manage, citing a combination of difficult definitions, variable significance, and the different amounts of information patients were felt likely to want. The potential for information overload for patients, complicating decision-making, was discussed. Some patients described the practical difficulties of HCP making disclosures in a time-limited consultation, and the burden then put on patients to effectively consider and/or manage them.

"*I can also see that people, perhaps, in a consultation, are overwhelmed with information about their health… And it's so very difficult to know how relevant it is and whether it's really something that is swaying their judgement or not." (P6, citizen)*

Concerns were expressed about workload for HCPs if more regulations were introduced, with extra time needed for disclosure and explanation rather than for direct patient care.

"*And also, these professionals are very precious to you, they don't have much time, and you don't want to be talking about conflicts of interest when you actually want them to help you to do what it is that they're going to do." (P5, activist)*

Participants suggested ways of managing COIs, including better transparency. Several participants suggested that a mandatory register or regulation, like a Sunshine Act (6) might be a useful way of managing COIs. Respondents also wanted to see the same rules applied across the whole NHS as variation across the health boards was seen to lead to fragmentation and variation in care.

"*I think there should be… This Sunshine Act in America they have to declare… And I thought well that's open to interpretation but at least it would be something, so it would make it more ethical." (P12, lay)*

However, systems were needed to deal with COIs in ways that accounted for medical power. Complexity was again reflected. Participants wondered if simple disclosure would make a difference to patients. For example:

"*Most patients won't ever question this. They will never question their doctors." (P2, lay)*

A view held by several participants was that managing COIs should not rest with patients, but be a professional duty. The multiplicity of interactions meant that patients should be protected because a simple statement of interests could not suffice.

"*But does that capture the reality of the communications between doctor and patient, where so much is non-verbal, or implicit? …Relationships with employer, relationship with fellow professionals, relationship with marketing reps and others. The more you look at it, the harder it gets, I found." (P3, lay)*

Participants felt that independent advocacy could aid vulnerable people in consenting to treatment, if there were concerns around COIs. Second opinions and the presence of independent advocates were also seen as ways to mitigate bias. Independent parties would be able to raise issues of concern in relation to COIs:

" I think I would need an advocate with me to discuss my options. So, that's somebody who understands the medical side, but also somebody who is trained in advocacy and is a patient advocate…" (P21, lay)

However, again, complexity was acknowledged as independence was not always guaranteed.

"*I work in the area of health communication….the challenge I'm finding is that most of the patient advocates are sponsored by drug companies. And they've no regulation around that. There's no guidelines…it just seems a bit murky to me and not transparent." (P7, activist)*

Some participants highlighted where a declaration of a conflict may not necessarily reveal a negative bias. This could lead to patient concern about a conflict that was not in fact significant.

"*that doesn't mean somebody's not a good person and they're not doing the right thing…you know, he really believes in all the stuff…But he must be biased by that. Is that good bias? Is that bad bias? I don't know, you know?…" (P15_Carer)*

Participants suggested 'spot checks' (*P17*, *activist*) or oversight by an independent body to ensure that HCPs do not submit unsubstantiated information. This contrasted with discussions around trying to limit additional bureaucracy. Checking, peer review, and training were suggested on how to recognise COIs and potentially reduce unconscious bias. Other professions, particularly the public sector, law, Parliament and academia were compared, reflecting a shared view that professionals had duties to organise effective management.

"*I think the way it could be solved is that doctors with direct financial interests don't take on cases in that particular area. Obviously, that's quite serious, but I can't think of any other way that it could be totally solved. You know, essentially like the legal profession, people recuse themselves from cases. So that's just essentially the same principle". (P18, citizen)*

Discussion:

This is the first study we are aware of to test whether declarations of interest, as currently made by UK based, publicly facing doctors, could be located by lay people and professionals. In the second phase of the study, in vignettes describing an increase in financial declarations, participants' confidence in the doctor acting in their best interests, and their trust, fell. While 85% of participants thought that knowing about professional declarations was 'definitely' or 'probably important', despite generous marking, 27% (58) participants found a relevant link to 'fit for purpose' information and only 2% (5) found all components of the 'gold standard'.

All the professionals taking part in the study were part of an organisation with an official need to publicly declare interests. This study has demonstrated that, despite the efforts of the NHS to improve practice (e.g. NHS Trusts holding public registers of interest), transparency is not being effectively achieved because participants were unable to locate the registers with reasonable ease.

The third phase of this study invited patients and citizens, who had participated in the second phase, to interview. Declarations and conflicts presented complex challenges. Participants described ways to improve the system, but were also concerned for practicality, opportunity cost and bureaucracy. This was particularly in terms of where declarations should be made, when they were relevant, and how patients could feasibly use these. Multiple trade offs were described. For example, free but sponsored education and training potentially resulted in professionals having more up-to-date knowledge but bias and/or conflicts of interest could be produced by these funders. Medical power had to be mitigated. Professionals may lack insight into their own bias, and independent oversight would be necessary to mitigate and check compliance. Some expressed concern of the additional burden that could be placed on doctors, and also patients to locate and interpret declarations. Free text responses in the searches confirmed this, for example "Despite searching I found no good way to find any" "I could not easily find anything apart from some stuff on Wikipedia" "Sorry, I struggle to find any. Giving up." "I am finding this task really difficult. There is too much information to work through. Patients should not have to do this research."

Strengths of this study include the collaboration of a patient panel to develop the survey, suggest professional participants, and disseminate invitations to participate. Additionally, it was highly pragmatic, mimicking the steps that a citizen would take to investigate a conflict. This is the first study we are aware of which tested current UK declaration processes. It is also the first we are aware of in the UK which interviewed laypeople to elucidate understanding and concerns about conflicts of interest in healthcare professionals, and what improvements could consist of. There were several limitations. The HPP were by definition in the public eye, and willing to take part, and may have been more likely to use a high profile register eg on government websites. However this would have resulted in an over estimation of being able to locate conflicts, meaning that the results would be artificially high. Only one conflict in each category (e.g. professional financial, indirect) had to be found to be scored correct, leading to an overestimation of the effectiveness of current practice. Declarations were scored equally, however, the relative importance of each declaration is not, in reality, equal: some may have been unimportant and very unlikely to cause important conflict; others, the opposite. Further, the participants and professionals who took part in our study are likely atypical, with engagement with these issues prior to the request for participation. It is not expected that many citizens would normally spontaneously search for healthcare professionals' declarations or conflicts. Our participants are likely to have engaged with some or many of the issues related to conflicts of interest, given that our patient group assisted in sending the questionnaire widely to engaged patient groups. Multiple entries to the questionnaire by one person under different email addresses would have been possible but we consider this overall unlikely to have had a large impact on results. Nevertheless, even in a group of activated professionals and citizens, finding a complete declaration of interest was extremely difficult, and a partial finding of declarations was possible only a minority of the time. Participants agreed, reporting a low level of confidence that their results were complete.

While there are no directly comparable studies, other US work (31) supports the finding that patients, including potential research participants, wish transparency and to know the researchers' conflicts of interest. The impact of disclosure of a doctor's conflicts of interest to patients via a mailed letter has been investigated in the US (32). Patients subsequently described an increased level of confidence in their ability to judge potential impacts of conflicts on their healthcare. Overall there was no change in described levels of trust in doctors. However, in patients who three months later recalled receiving the disclosure notice, around a fifth described increased trust in the physician. Given what is known about the potential for harm from financial conflicts of interest, it is questionable whether this is a good outcome, as people may trust advice even if it is at risk of bias. Sah et al. (33) investigated trust in scenarios, mainly concerning financial advice. People were asked about their levels of trust in financial advisors where the advisor had concealed or unconcealed conflicts of interest, and where the interests of client and advisor were aligned or not. Trust was found to be reduced when a conflict of interest was known, even when the interest of client and advisor was congruent and the advice was high quality. The final scenario concerned a medical vignette. This found that a disclosure of a conflict resulted in increased trust in participants. However the vignette featured a doctor's recommendation not to do a test, which would otherwise have attracted a fee. This so-called 'altruistic signal' is theorised to offset the 'disclosure penalty' which can otherwise reduce trust. Finally, a field experiment in the US (34) randomised patients to receive a hospital appointment letter containing, or not, the doctor's conflict of interest statement. Patients receiving the disclosure reported more knowledge of these conflicts, with no change in trust or appointment attendances. These studies were set in the US, where there are major cultural differences concerning health service delivery, and are unlikely to be directly applicable to dissimilar countries such as the UK.

This research has found that lay people hold mixed and often nuanced views over conflicts of interest. Further, the practical aspects of declarations, including organisation, workload for both patients and doctors, and interpretation, was realised to present difficulties. Given the strong decline in trust in the

For peer review only - http://bmjopen.bmj.com/site/about/guidelines.xhtml

vignettes with increasing conflicts of interest, it is uncertain whether an 'altruistic signal' would compensate for a 'disclosure penalty'. Further, the 'medical power' which laypeople alluded to must still be negotiated where conflicts are found, an aspect not investigated in these other studies, although Pearson et al. (35) found that half the patients who remembered seeing a disclosure did not feel they knew enough to judge the potential impact of it.

This leads to basic questions about the use and purpose of declarations of interest in the UK. Should they be for transparency alone? Should declarations be intended as more than an 'information dump' but made in ways which enable judgements - and effective management?

Unintended consequences of transparency are possible, for example, 'moral licence', where disclosure is assumed to negate potential bias. There is evidence that doctors believe that other doctors become biased when exposed to small gifts, while they themselves do not (36). Further, the survey and interviews confirmed that a lack of transparency in professionals leads to less trust for patients. If more transparency was created, and trust in conflicted doctors increased, it is uncertain whether this would be justified, given the evidence that financial conflicts of interest are associated with bias, and more expensive poorer quality healthcare (37, 38). Indeed, previous research has found that declarations of interest, for example, those recommended by the International Committee of Medical Journal Editors, are of poor quality and make it difficult for the reader to assess bias (39).

Transparency may therefore not be a benign act. Given that most UK Hospital Trusts do not state the action planned to mitigate a publicly declared conflict, it may not be clear where the work of finding, interpreting and managing conflicts of interests rests (21). Many respondents were concerned as to what to do with the conflicts located. Some felt they could trust the doctor regardless of a conflict, as disclosure mitigated bias; others felt it difficult to know whether they could trust the doctor's judgement despite a declared conflict, and would require advocacy to assist. While patient organisations were suggested as potential advocates, there is also evidence that some are themselves conflicted (40).

The General Medical Council (UK) recommends doctors "avoid conflicts of interest wherever possible" and "declare any conflict to anyone affected, formally and as early as possible, in line with the policies of your employer or the organisation contracting your services". They also recognise "Conflicts of interest are not always avoidable… follow(ing) established procedures for declaring and managing a conflict" (13). The risk is that disclosure is used to include rather than exclude individuals from relevant decision making, in the belief that a disclosure constitutes management. While disclosure is necessary for management, it does not substitute for it. This is particularly important when considering the evidence on unconscious bias from professionals, and the risk of trusting conflicted, but declaring, professionals where patients have limited power to know about or mitigate the potential impact of a conflict.

Further research should elucidate what the purpose(s) of declarations of interest should be for different groups of people and find ways to meet these needs. For example, if it is for simple transparency, declarations need to be easy to find and understand. If they are to manage conflicts, a clear decision on boundaries may be helpful. However, changes should be tested not just with patients, but professionals, as issues of workload and opportunity cost were reflected in interviews. Further, research should help to understand the best ways of making declarations which allows the reader to make an evidence-based interpretation of their potential impact. Our study relied on interested parties to participate, and research on panels recruited from the wider population would be helpful. Professional views should also be sought to understand what the facilitators and barriers are to making declarations in order to organise the best way to declare and manage them. Finally, the value of making declarations has yet to be established, beyond transparency. A more reliable way to manage them may be via better processes of disallowing certain

For peer review only

conflicts from defined roles, rather than trying to manage them using haphazard declarations of interest. Further qualitative work may help to understand how conflicts are managed in practice.

Conclusion

Declarations of interest by professionals are agreed to be important, but are unfit for purpose in their current form. The survey found that patients describe trusting professionals with no conflicts the most, and professionals with undisclosed conflicts the least. The practical task of finding declarations of interest for well-known doctors in the public domain was difficult. Even when disclosure statements were found, most were incomplete when compared to the 'gold standard', which were rarely located. Interviews with lay people found nuanced views about disclosure and management of conflicts. They were described as important, but difficult to find and use, and although some potential conflicts could be justified, they needed to be managed. Currently practitioners making declarations cannot be assured that this information can be readily found, and cannot assume that this information can be used in decision making by laypeople. Other research finds that transparency may result in unintended consequences, including placing trust in professionals who may take 'moral licence' from an open declaration, while increasing workload for patients and professionals. Together, these may cause unintended harms. Patients may not feel able to use information about conflicts of interest to their advantage. This means that declaring potential conflicts should be refined and greater professional emphasis of avoiding, identifying and managing serious conflicts of interest with clear, public definitions on who requires exclusion from what types of decision making, be considered. It is questioned whether the purpose of declarations should rest on providing mere transparency, but be used to exclude, rather than include, conflicted professionals in relevant decision making.

Acknowledgements

We thank the health professionals who took part in this study for their time and valuable assistance and support; our patient panel, Lynne Craven, Brian Finney, Fiona Raje, and Kath Samson for their help with study design, invitations and recruitment, and Professor Carl Heneghan who discussed and advised on the study at conception. We also thank the peer reviewers for their constructive and helpful comments.

Data sharing

No further data is available. This is because our HPP gave consent on the basis of confidentiality of their identity, which would not be possible if further data was made public.

Contributorship

Study conceived by MM with design by MM and FS. Questionnaire design by MM, RM, GO and AMB with assistance and direction from the patient panel. Analysis of questionnaire and survey data by RM, MM and FS. Qualitative interviews by KM and MM. Analysis of interviews by KM, AMB, GO, MM. Draft of paper by MM with editing and contributions from all.

Copyright

The Corresponding Author has the right to grant on behalf of all authors and does grant on behalf of all authors, an exclusive licence (or non exclusive for government employees) on a worldwide basis to the BMJ Publishing Group Ltd to permit this article (if accepted) to be published in BMJ editions and any other BMJPGL products and sublicences such use and exploit all subsidiary rights, as set out in our licence.

Transparency declaration

For peer review only

MM affirms that the manuscript is an honest, accurate, and transparent account of the study being reported; that no important aspects of the study have been omitted. We had originally planned to run the online questionnaire for 4 weeks but extended to 8 weeks as we were still receiving responses.

Protocol

This is enclosed as per the registration document. It is marked Appendix 10.

Checklist

We enclose CROSS checklist (for survey designs) and SRQR (for qualitative research) as recommended by EQUATOR.

References

1. Taichman DB, Backus J, Baethge C, et al. A Disclosure Form for Work Submitted to Medical Journals: A Proposal From the International Committee of Medical Journal Editors. JAMA. 2020;323(11):1050–1051. doi:10.1001/jama.2019.22274

2. NHS England publish new guidelines on tackling conflicts of interest. 9/2/17 https://www.england.nhs.uk/2017/02/coi-guidelines/

3. Spithoff S, Leece P, Sullivan F, Persaud N, Belesiotis P, Steiner L. Drivers of the opioid crisis: An appraisal of financial conflicts of interest in clinical practice guideline panels at the peak of opioid prescribing. PLoS One. 2020;15(1):e0227045. Published 2020 Jan 24. doi:10.1371/journal.pone.0227045

4. Wang AT, McCoy CP, Murad MH, Montori VM. Association between industry affiliation and position on cardiovascular risk with rosiglitazone: cross sectional systematic review. BMJ. 2010;340: c1344–c1344. 10.1136/bmj.c1344

5. Fickweiler F, Fickweiler W, Urbach E. Interactions between physicians and the pharmaceutical industry generally and sales representatives specifically and their association with physicians' attitudes and prescribing habits: a systematic review. BMJ Open. 2017;7(9):e016408. Published 2017 Sep 27. doi:10.1136/bmjopen-2017-016408

6. UK Research and Innovation. How we're governed: Conflicts of interests. 12/5/22 https://www.ukri.org/about-us/how-we-are-governed/conflicts-of-interests/

7. First do no harm: The Report of the Independent Medicines and Medical Devices Review. 8/7/20 https://www.immdsreview.org.uk/Report.html

8. The influence of the Pharmaceutical Industry. 2014/5 session Health Select Committee UK Parliament https://publications.parliament.uk/pa/cm200405/cmselect/cmhealth/42/42.pdf

9. The sun shines on Europe. Transparency of financial relationships in the healthcare sector. 2017 Health Action International https://haiweb.org/wp-content/uploads/2017/03/Sunshine-Act.pdf

10. Fabbri, A., Santos, A., Mezinska, S., Mulinari, S., Mintzes, B. (2018). 'Sunshine Policies and Murky Shadows in Europe: Disclosure of Pharmaceutical Industry Payments to Health Professionals in Nine European Countries', International Journal of Health Policy and Management, 7(6), pp. 504-509. doi: 10.15171/ijhpm.2018.20

11. Kanter GP, Carpenter D, Lehmann L, et al Effect of the public disclosure of industry payments information on patients: results from a population-based natural experiment BMJ Open 2019;9:e024020. doi: 10.1136/bmjopen-2018-024020

For peer review only

12. Policy on declaring and managing interests for NICE advisory committees. National Institute for Health and Care Excellence. September 2022 https://www.nice.org.uk/Media/Default/About/Who-we-are/Policies-and-procedures/declaration-of-interests-policy.pdf

13. Financial and commercial arrangements and conflicts of interest. General Medical Council. Accessed 10/2/23 https://www.gmc-uk.org/ethical-guidance/ethical-guidance-for-doctors/financial-and-commercial-arrangements-and-conflicts-of-interest/financial-and-commercial-arrangements-and-conflicts-of-interest

14. Lo B, Field MJ. Conflict of interest in medical research, education and practice. Institute of Medicine of the National Academies, Committee on Conflict of Interest in Medical Research, Education and Practice. IOM (Institute of Medicine) 2009. https://nap.nationalacademies.org/read/12598/chapter/1#ii

15. Wilson M. (2014). The Sunshine Act: commercial conflicts of interest and the limits of transparency. Open medicine : a peer-reviewed, independent, open-access journal, 8(1), e10–e13.

16. The PLoS Medicine Editors (2012) Does Conflict of Interest Disclosure Worsen Bias? PLoS Med 9(4): e1001210. https://doi.org/10.1371/journal.pmed.1001210

17. Susannah L. Rose, Sunita Sah, Raed Dweik, Cory Schmidt, MaryBeth Mercer, Ariane Mitchum, Michael Kattan, Matthew Karafa, Christopher Robertson, Patient responses to physician disclosures of industry con-flicts of interest: A randomized field experiment, Organizational Behavior and Human Decision Processes, 2019, ISSN 0749-5978 https://doi.org/10.1016/j.obhdp.2019.03.005.

18. Pearson SD, Kleinman K, Rusinak D, Levinson W. A trial of disclosing physicians' financial incentives to patients. Arch Intern Med. 2006 Mar 27;166(6):623-8. doi: 10.1001/archinte.166.6.623. PMID: 16567600

19. Hampson et al. Patients' views on financial conflicts of interest. N Engl J Med 2006; 355:2330-2337 DOI: 10.1056/NEJMsa064160

20. Kmietowicz Zosia. Disclosure UK website gives "illusion of transparency," says Goldacre BMJ 2016; 354 :i3760

21. McCartney M, Bergeron Hartman R, Feldman H, et alHow are declarations of interest working? A cross-sectional study in declarations of interest in healthcare practice in Scotland and England in 2020/2021BMJ Open 2022;12:e065365. doi: 10.1136/bmjopen-2022-065365

22. Macdonald Helen, McCartney Margaret, Heneghan Carl, Godlee Fiona. Doctors' conflicts of interest BMJ 2020; 370 :m3247

23. Fadlallah R et al. Knowledge, Beliefs and Attitudes of Patients and the General Public towards the inter-actions of Physicians and the Pharmaceutical and Device Industry: a systematic review. PLOS 24/8/1

24. Glogowska, M. (2011), Paradigms, pragmatism and possibilities: mixed-methods research in speech and language therapy. International Journal of Language & Communication Disorders, 46: 251-260. https://doi.org/10.3109/13682822.2010.507614

25. Bishop FL. Using mixed methods research designs in health psychology: an illustrated discussion from a pragmatist perspective. Br J Health Psychol. 2015 Feb;20(1):5-20. doi: 10.1111/bjhp.12122. Epub 2014 Nov 18. PMID: 25405549.

26. Barriball, LK. and While, A. (1994), Collecting data using a semi-structured interview: a discussion paper. Journal of Advanced Nursing, 19: 328-335. https://doi.org/10.1111/j.1365-2648.1994.tb01088.x

27. Glogowska, M. (2011), Paradigms, pragmatism and possibilities: mixed-methods research in speech and language therapy. International Journal of Language & Communication Disorders, 46: 251-260. https://doi.org/10.3109/13682822.2010.507614

For peer review only

28. Bishop FL. Using mixed methods research designs in health psychology: an illustrated discussion from a pragmatist perspective. Br J Health Psychol. 2015 Feb;20(1):5-20. doi: 10.1111/bjhp.12122. Epub 2014 Nov 18. PMID: 25405549

29. Barriball, LK. and While, A. (1994), Collecting data using a semi-structured interview: a discussion paper. Journal of Advanced Nursing, 19: 328-335. https://doi.org/10.1111/j.1365-2648.1994.tb01088.x

30. Braun V, Victoria Clarke V. Using thematic analysis in psychology, Qualitative Research in Psychology, 3:2, 77-101, (2006) DOI: 10.1191/1478088706qp063oa

31. Kim SY, Millard RW, Nisbet P, Cox C, Caine ED. Potential research participants' views regarding researcher and institutional financial conflicts of interest. J Med Ethics. 2004 Feb;30(1):73-9. doi: 10.1136/jme.2002.001461. PMID: 14872080; PMCID: PMC1757115.

32. Pearson SD, Kleinman K, Rusinak D, Levinson W. A trial of disclosing physicians' financial incentives to patients. Arch Intern Med. 2006 Mar 27;166(6):623-8. doi: 10.1001/archinte.166.6.623. PMID: 16567600

33. Sah S, Loewenstein G, Cain D. Insinuation Anxiety: Concern That Advice Rejection Will Signal Distrust After Conflict of Interest Disclosures. Pers Soc Psychol Bull. 2019 Jul;45(7):1099-1112. doi: 10.1177/0146167218805991. Epub 2018 Nov 17. PMID: 30451077

34. Rose SL, Sah S, Dweik R, Schmidt C, Mercer M, Mitchum A, Kattan M, Karafa M, Robertson C. Patient responses to physician disclosures of industry conflicts of interest: A randomized field experiment. Organizational Behavior and Human Decision Processes,
Volume 166, 2021, Pages 27-38, ISSN 0749-5978, https://doi.org/10.1016/j.obhdp.2019.03.005.

35. Pearson SD, Kleinman K, Rusinak D, Levinson W. A trial of disclosing physicians' financial incentives to patients. Arch Intern Med. 2006 Mar 27;166(6):623-8. doi: 10.1001/archinte.166.6.623. PMID: 16567600

36. Steinman MA, Shlipak MG, McPhee SJ. Of principles and pens: attitudes and practices of medicine housestaff toward pharmaceutical industry promotions. Am J Med. 2001 May;110(7):551-7. doi: 10.1016/s0002-9343(01)00660-x. PMID: 11347622.

37. Nejstgaard CH, Bero L, Hróbjartsson A, Jørgensen AW, Jørgensen KJ, Le M, Lundh A. Association between conflicts of interest and favourable recommendations in clinical guidelines, advisory committee reports, opinion pieces, and narrative reviews: systematic review. BMJ. 2020 Dec 9;371:m4234. doi: 10.1136/bmj.m4234. PMID: 33298430; PMCID: PMC8030127.

38. Spurling GK, Mansfield PR, Montgomery BD, Lexchin J, Doust J, Othman N, et al. Information from pharmaceutical companies and the quality, quantity, and cost of physicians' prescribing: a systematic review. PLoS medicine. 2010;7(10):e1000352. pmid:20976098; PubMed Central PMCID: PMC2957394.

39. Grundy Q, Dunn A G, Bero L. Improving researchers’ conflict of interest declarations BMJ 2020; 368 :m422 doi:10.1136/bmj.m422

40. Mandeville K L, Barker R, Packham A, Sowerby C, Yarrow K, Patrick H et al. Financial interests of patient organisations contributing to technology assessment at England’s National Institute for Health and Care Excellence: policy review BMJ 2019; 364 :k5300 doi:10.1136/bmj.k5300

For peer review only

Appendix Table 1

Description of the interviewees by sex, age and self defined role

	Males	Females
Age group		
30-39	1	0
40-49	0	1
50-59	2	4
60-69	2	8
70-79	1	0
80+	2	0
Total	8	13
Status		
Citizen	1	3
Patient	2	2
Long term condition	0	2
Patient activist	2	2
Carer	0	1
Not specified	3	3
Total	8	13

For peer review only

Appendix 1:

Method of searching for declarations/conflicts of interest

General Google search for job title, roles, affiliations - first 10 pages “Firstname Lastname” + “NHS” + “private” + “clinic” + “press release”

“Firstname lastname” + “Conflicts of interest” + “declared interests”

Disclosure UK (APBI)

NHS Trust site / GP surgery site / any private clinics - search for Register of Interests, and search within, if not publicly available need to write to NHS Trust using FOI if necessary

NICE/SIGN search for 1) Firstname Lastname 2) if present, search for DOI

PubMed Firstname Lastname - search for declaration of interest

Linkedin

Companies House - UK registration of all limited companies

Electoral Commission - personal or company donations

Patents (Google Patents - patents.google.com)

LexisNexis (any mentions of companies / sponsors)

TV / Radio (transcripts accessible via Box of Broadcasts)

PubMed: Journal articles in last 5 years, conflicts of interest declarations

ClinicalTrials

OpenPayments.gov

Youtube lectures for any declarations of interest made

For peer review only

Appendix 2

Methods of recruiting lay people, patients, citizens and professionals to participation

1) Citizens - invited via social media and citizen groups including Sense about Science network
2) Patient activists - recruited via National Voices, Sling the Mesh, Patients Association UK
3) Healthcare students - via Medical Schools Advisory Council, nursing, midwifery, and paramedical school associations via social media, and via ELAG (Educational Leads Advisory Group) to contact medical students, medical school newsletters.
4) Healthcare professionals working independently eg consultants, pharmacists, independent nurse consultants/prescribers — via social media, medical press, medical school newsletters
5) Early career researchers - training forums, Colleges via social media.

For peer review only

Appendix 3

Vignettes used in online questionnaire

A

You have developed a problem with your hip. You go to see a doctor, who you have known for several years and they have always given good care. They are qualified to do their job.

The doctor recommends an operation in the NHS to replace your hip.

This is a type of hip replacement called Mars. You are told that this is the standard type of hip replacement used for people like you and usually has very good results.

The doctor tells you that they do not have any shares or patents in Mars, nor do they get paid extra to fit this type of hip replacement.

B

You have developed a problem with your knee. You go to see a doctor, who you have known for several years and they have always given good care. They are qualified to do their job.

The doctor recommends an operation in the NHS to replace your knee.

This is a type of knee replacement called Jupiter. You are told that this is the standard type of knee replacement that is used for people like you and usually has very good results.

The doctor tells you that they do not have any shares or patents in Jupiter. However the doctor says that they have been to a conference about this type of knee replacement. The company paid for them to travel to a conference in Europe and for a course about it, which would normally have cost a few thousand pounds.

C

You have developed a different problem with your other hip. You go to see a doctor, who you have known or several years and they have always given good care. They are qualified to do their job.

The doctor recommends an operation in the NHS to replace your hip with a type of hip replacement called Venus.

You are told that this is the standard type of hip replacement for people like you and usually has very good results.

The doctor tells you that they are part of the team that developed Venus hip replacements. The doctor works as a consultant for the company that makes them and travels to

For peer review only

conferences to explain the hip replacement to other doctors. The doctor is a part-owner of the patent for Venus hip replacement and has shares in the company making them.

D

You have developed a different problem with your other knee. You go to see a doctor, who you have known or several years and they have always given good care. They are qualified to do their job.

The doctor recommends an operation in the NHS to replace your knee.

You are recommended a type of knee replacement called Saturn. You are told that this is the standard type of knee replacement used for people like you and usually has very good results.

You do a search online. You find that this doctor owns a patent and shares in Saturn. Last year the doctor received £50,000 for working as a consultant for the company.

For peer review only

Appendix 4

Criteria included in 'gold standard'

As many participants listed potential conflicts as items which were out of scope of ICMJE criteria/ NHSE criteria some were dismissed; for example:

Director of company dissolved >5 years ago
Single talk given to public >3 years ago
Public service job > 5 years ago
Honorary Fellow/Member of medical organisation
Examiner for a Royal College exam (reasonably supposed to be part of a job already declared)

For peer review only

Appendix 5

Estimation of how many forms filled in per year to declare interests by healthcare professionals

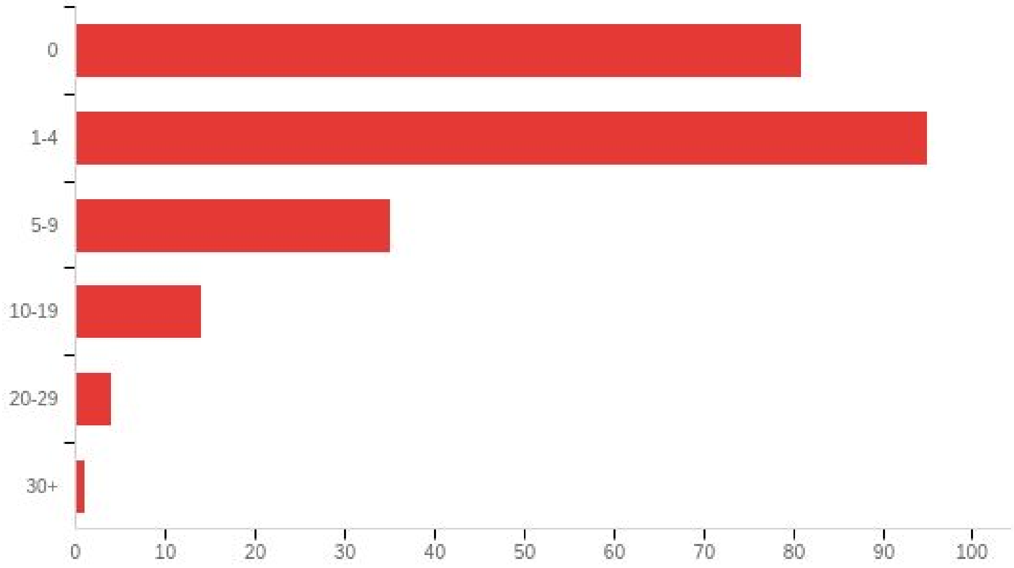

review only

Appendix 6

Relationship of Trust to increasing conflicted scenarios

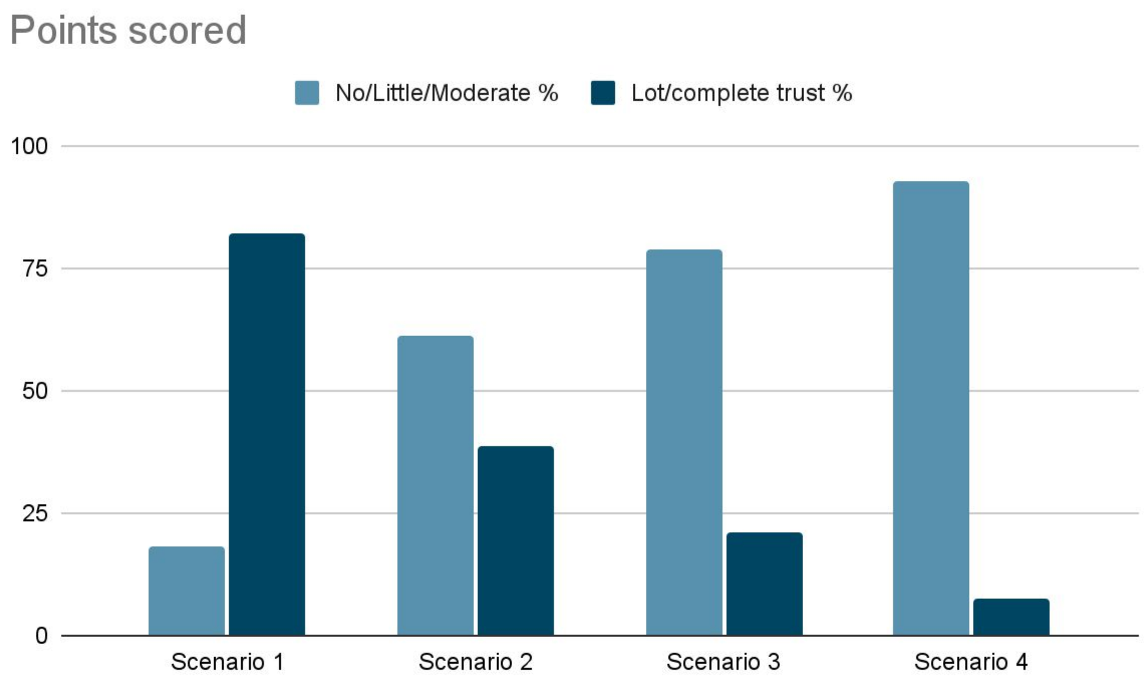

A	B	C	D	E
	No/Little/Mode	Lot/Complete trust		
Scenario 1	18.1%(64)	81.9% (289)		
Scenario 2	61.2% (211)	38.8%(134)		
Scenario 3	79% (270)	21.1% (72)		
Scenario 4	92.7% (315)	7.4% (25)		

Appendix 7

Relationship of belief doctor acting in patients best interest to increasing conflicted scenarios

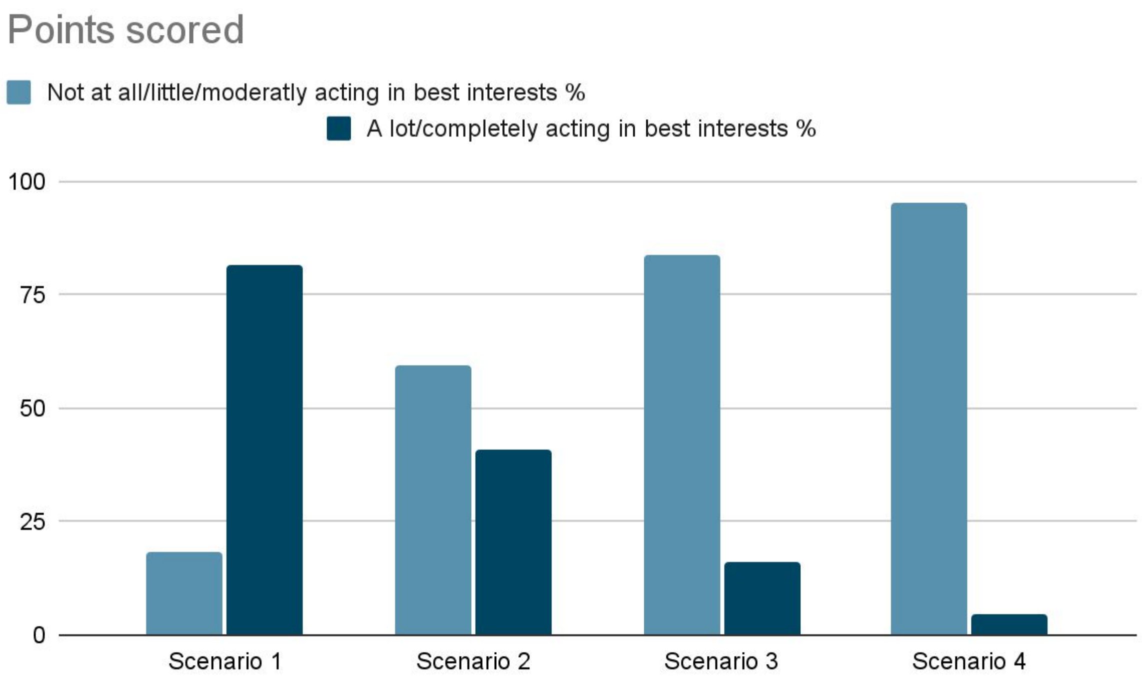

Scenario 1	18.5(65)	81.6 (287)
Scenario 2	59.3(204)	40.7 (140)
Scenario 3	83.7(288)	16.3(56)
Scenario 4	4.7 (16)	95.3 (324)

Appendix 8

Results - participants locating fit for purpose and gold standard declarations

Key:

‘Links located” - total number of links reported by research participants

“Number fit for purpose” - weblinks containing a formal declaration of interests

“Participants reporting any results” - any information obtained was included

“Participants reporting a fit for purpose link” - number of participants finding a ‘fit for purpose’ link

“Significant entries%” - all significant entries including financial entries

Responses finding all categories - at least one entry from each category

Professional	Links located	Number fit for purpose	Participants reporting any results	Participants finding a fit for purpose link	Average% significant interests found	Total responses containing each category of declaration
A	11	3/11	14/24	2	64%	8/14
B	14	3/14	12/24	6	57%	8/12
C	5	1/5	10/22	1	30%	3/10
D	6	5/6	10/21	5	90%	9/10
E	10	1/10	9/23	3	67%	6/9
F	9	6/9	14/24	8	79%	11/14
G	19	2/19	17/22	3	18%	3/17
H	17	3/17	15/22	2	20%	3/15
I	3	2/3	11/23	4	55%	6/11
J	8	2/8	16/23	10	75%	12/16
K	12	1/12	10/22	2	70%	7/10
L	12	4/12	16/25	11	100%	16/16
M	10	2/10	13/22	2	15%	2/13

For peer review only

Appendix 9

Additional qualitative data from interviews arranged by theme

Theme 1: COIs in healthcare can be difficult to define

"It means that either directly or indirectly, a health care professional stands to benefit from a decision that is made about the appropriate treatment for a patient". (P16_F60-69_LTC)
"It means to me, that a professional may have a financial interest in a firm which is manufacturing drugs or medical devices and may or may not declare that interest to the people that they are caring for or prescribing for". (P21_F60-69_Lay)
"It could be having a bit of a conference with a dinner or a few drinks or giving a few products free. They think it's their right" (P14_F50-19_Lay)
"you can have a conflict between the need for public health …. and the needs to provide services for the person in front of you because it's an opportunity cost. … it's balancing out …. the exigent circumstances of the person in front of you or the group you're talking about it one time." (P16_F60-69_LTC)
"It could be not direct to them. It might be a contribution towards research to which they have an interest. So, they think it's acceptable because it's going towards research, but it's clearly swaying the opinion and the reason behind things". (P14_F50-19_Lay)
"I think people struggle with understanding what a conflict of interest is because you could have an interest in something and not have any financial gain from it and not declare that interest …. I think on the whole, a lot of people don't declare interests because they don't really understand what their interests are and how they conflict". (P10_M60-69_Activist)
"Well, at the extreme end, it means doctors being involved with the direct purchase of equipment or drugs that they have an interest in that company, so they therefore are likely to get greater dividends back. Or if they're actually part owners of companies, … they're getting funds directly from that company either through profits or when the company is sold. So that's a very strong conflict of interest". (P17_M60-69_Activist)
"one of the areas I thought of was where your beliefs, your beliefs will influence what you do. And I think that that we don't look at that an awful lot. … So I'd be very, very careful that my unconscious bias doesn't conflict with professionalism, and I think that that's a really difficult area because you're talking about thoughts, you know, rather than financial interests and sometimes you know that can be more insidious than, you know, money". (P10_M60-69_Activist)
"So, I actually think healthcare professionals don't understand what conflict of interest means. I think it's that fundamental. They don't understand biases in science. They don't understand their need for not only reflection, but reflexivity. That they need to reflect on their own values and beliefs in what they're bringing to the evidence that they're presenting to patients". (P7_F50-59_Activist)

For peer review only

"It's trust. This is what I said about bribery and all that type of thing. It's very sad because if you're in the health profession, I always feel that these people should be within the heart, caring. It's all about being a caring person." (P14_F5-59_Lay)
"Personally, unfortunately, I've lost all trust of all items due to, I feel, a conflict of interest, what happened to me. I always research everything. In fact, I refuse it now, anything. To a point you lose total trust with everything, and it can ruin people's trust in things, in medication, so it's key." (P14_F5-59_Lay)
"*I know very little about it…. It makes me think of when drug companies go round, and they do a lunch for the doctors and they do a presentation about their product and the doctors prescribe their product. But I feel it's much wider and there's lot of other circumstances…I don't really know much about them". (P1, lay)*
"*It's almost like a bribe, possibly, from the pharmaceutical… It could be a soft one, where maybe the medical doesn't recognise that it's happening, or it could be something that's really quite obvious but they think it's acceptable because there's no law there." (P14, lay)*
"*...it's to do with the power imbalance. You have gone to this professional for help because you don't know much about what is happening to you. So, it's about trust… you trust them to do the right thing for you, because you don't know how to do it yourself." (P5, activist)*

Theme 2: COIs in healthcare can be hard to find

"...if a new product is brought to the market, then, understandably, if it's going to be used it needs to be promoted, so that's understandable. But I don't think it should be too difficult to have a system of transparency around that." (P1_F40-49_Lay)
"I think that the openness and transparency should apply equally to both financial and non-financial, and I don't think… I suppose, financial, you assume is worse, if you like, or more likely to be not in my best interest, but I think, actually, both equally need to be clear as to what's happening." (P5_F60-69_Activist)
" … so in terms of conflict of interest, I don't think it's necessary for the patient to do a huge amount more. I think the NHS is aware of the issue and they've produced reports and whatever on the subject in past years, I believe. For me, the thing that's needed, and you know, I'm not sure how much this is in place, is the transparency and whistleblowing and ability to contest after the event …" (P13_M70-70_Lay)

Theme 3: COIs present both challenges and benefits for patients

For peer review only

“ … they got their paid research grants, and they were thinking well we could do this, or we could do that, but now, I look back, I think that wasn’t really in the patient's interests. Because the patient was bamboozled with choices that she knew nothing about. … And you were still trusting implicitly that they had your best interests at heart when I think they were looking at interesting training opportunities for medical students. … I used to have a career and a nice home. I've lost everything to this undisclosed bias in favour of polypropylene implants.” (P12_F60-69_Lay)

“I think when there are conflicts of interest, there's potential for corruption. Potential for corruption is always a problem... It depends on the rules that you have in place that stop corruption. … For me it depends on what becomes from the norm and acceptable. … And in some of these conflicts of interest issues patients are moved into a commercial transaction where the distinction between the diagnosis and treatment and the offer is being blurred. And you're then into being sold a product which you might not completely need.” (P19_F60-69_Lay)

“i's that complexity because it's about, you know, not assuming that the conflict of interest is a bad thing for everybody as well. Sometimes. Well, actually that might be good thing, cause I believe that and I want somebody who also believes that.” (P15_F50-59_Carer)

“I don't have any huge problem. And I think my general view of health is that the right provider is sometimes more important than keeping it in house, and you know the NHS tends to keep things in house rather than use it outside, but that’s nothing, that profit in itself is not a not a dirty word…” (P13, lay)

“Because it can affect outcomes for people’s care. There's been incidences of, for example, poor devices being employed, hip replacement joints, I think there was a bad set of them at one point. One assumes that they were being promoted by the company at the time. The vaginal mesh incidence, again, one assumes across the gynaecological board, they were being hyped as the best thing. And that has ruined people’s lives, in fact, it’s killed some people.” (P9, lay)

Theme 4: COIs need to be carefully managed

“So if I was looking for a private treatment for something that I might well go and look, you know, there wasn't an everyday, not like a dentist or something like that. But I might then go and look at that person, that individual and sort of look at at what they do and why they do it. And you can often see that, you know, things that come up on Facebook and places. There are clear links between.” (P15_F50-59_Carer)

“I think it's much more complex issue than that as we're dealing with human beings and it's really hard to unpick. And I think you have to go, I guess what you have to do is you have to go with the worst case scenario that somebody who's got… You know, some kind of major conflict of interest will abuse that and therefore you have to put such systems and structures in place that avoid that worst case scenario.” (P15_F50-59_Carer)

For peer review only - http://bmjopen.bmj.com/site/about/guidelines.xhtml

"I don't think I would have known that you would find that…and so I suspect that if I don't know, and I have got a background in the pharmaceutical industry, and medical friends and so on, I suspect that the layperson would have very little idea that that was there to be found. So, I'm not sure that expecting people to go and search online would be the best way to manage it. I think, probably, you'd have to be even more upfront and declare it at the consultation." (P6_F60-69_Lay)
"Actually, I'm not even given the name of consultants a lot of the time if I go to hospital, but if I'm seeing a consultant, all their interests should be there. I shouldn't have to go looking for it. It should be very easy to find" (P21_F60-69_Lay)

"I think if somebody declared something, they can always declare part of the truth, because the person who's declaring can choose. They can choose how they phrase it. They can leave things out. So, to me, it seems pointless." (P2_F50-59_Lay)
"But my problem with the whole setting up of a registering of interest, is the fact that it's voluntary. Most patients won't ever question this. They will never question their doctors. So, however much information there is, however available and easy you make it to find, people aren't going to look for it, because I trust it. I trusted the medical establishment before I started working for (name of organisation), and now I don't. I really don't." (P2_F50-59_Lay)
"that doesn't mean somebody's not a good person and they're not doing the right thing…you know, he really believes in all the stuff…But he must be biased by that. Is that good bias? Is that bad bias? I don't know, you know? But actually, if you saw him as a clinician, you should probably know…" (P15_F50-59_Carer)
"I think that we probably need to ensure that, particularly people who are responsible for those decisions, need to be trained well in understanding what conflicts of interests are. They need regular training, regular updates, and I think that they need to be challenged on a regular basis. … I think every year you probably need to go through a conflict of interest, you know, in your work. And I would say that it would be better to do it with someone, rather than by yourself" (P10_M60-69_activist)

For peer review only

"… most companies, you know, outside of the health care, they have to declare their interests and you can find out what their interests are by looking at them on the Internet. Health is actually one of those areas, it's very, very grey and shady and not saying that it's bad, it's grey. We haven't really done an awful lot around health and care." (P10_M60-69_activist)
"the mandatory things are problem in some ways, because people will find a way around it and but they'll find a way around it if it's not compulsory as well. Although the issue about it being not compulsory is if you can get the message out there and somebody hasn't." (P15_F50-59_Carer)
"… public members can then raise concerns to the board of the RMC or their BMA, or the ICN and say, look, you know this, this drug which we're using is seen in like a 70 to 80% uptake or an increase in prescriptions. Is this something normal?" (P11, lay)
I'm aware that, for example, if I were to read an article in, say, the British Medical Journal, which I don't do very often, but that professionals are required, or writers are required, to declare any interests that they have. So that the readers can make a judgement about whether or not that interest has influenced their research or writing. (P21,lay)

For peer review only

Study Information

Hypotheses

1) It is currently unreasonably difficult for individuals to search for information about health professionals declaration/conflicts of interest given the current UK system;

2) Understanding of the potential impacts of conflicts of interest is poorly understood by professionals, students and patients/citizens.

Design Plan

Study type

Experiment - A researcher randomly assigns treatments to study subjects, this includes field or lab experiments. This is also known as an intervention experiment and includes randomized controlled trials.

Blinding

- No blinding is involved in this study.

Is there any additional blinding in this study?

n/a

Study design

We will create a list of approximately 12-15 health professionals who have given recent advice or information to citizens in the public domain and who therefore may have come to the attention of the public. These will be a mixture of healthcare officials (for example, CMOs or deputy CMOs) heads of royal colleges, professional societies, pressure groups, NICE and SIGN guideline authors, and doctor journalists/columnists. We will ask for permission from each to take part. If agreed, we will do in depth searches for declarations and conflicts of interest for each professional. We will also include professionals who are not doctors, such as prominent senior NHS managers or Chief Nurses. We will ask each professional to check their declaration against ours and from this we will create a 'gold standard' against which we will compare to the results of participants searches. This will include:

General Google search for job title, roles, affiliations - first 10 pages
"Firstname Lastname" + "NHS" + "private" + "clinic" + "press release"

"Firstname lastname" + "Conflicts of interest" + "declared interests"

Disclosure UK (APBI)

NHS Trust site / GP surgery site / any private clinics - search for Register of Interests, and search within, if not publicly available need to write to NHS Trust using FOI if necessary

NICE/SIGN search for 1) Firstname Lastname 2) if present, search for DOI

PubMed Firstname Lastname - search for declaration of interest

Linkedin

Companies House

Electoral Commission - personal or company donations (here)

Patents (Google Patents - patents.google.com)

LexisNexis (any mentions of companies / sponsors)

TV / Radio (via Box of Broadcasts)

Pubmed: Journal articles in last 5 years, conflicts of interest declarations

Foreign databases

French (search by beneficiary here)

ClinicalTrials

OpenPayments

We will record the time taken to complete this search and 10% will be double checked by different researchers to ensure consistency. As part of this search, we will create a rolling spreadsheet of generic locations where conflicts and declarations were found. This will help to demonstrate the workload required in a search strategy. The purpose of this search is to be as complete as possible and may require repeated iterations. This is regarded as part of the research project to investigate the hypothesis that searching for declarations is unreasonably burdensome.

We will ask the healthcare professionals who have taken part in this project to check the declaration we have created for them. We will alter it as necessary to ensure accuracy. We will document the difference between each.

We will create a website. This will

- explain the problem of finding and locating declarations/conflicts of interest of healthcare professionals.

- seek participation and permission to obtain data about their age, sex, and professional/ patient / citizen background, and how they heard about this project.

- randomly allocate each participant to one professional on the list. We will give them a small amount of information about the person in question eg 'Firstname Lastname, a doctor from the UK who is head of guidelines at NICE'.

To do this we will ask participants their

- age group

- sex

- whether they are responding as a citizen/healthcare student/patient/ healthcare professional prescriber / healthcare professional / higher specialist trainee

- we will ask healthcare professionals : speciality, research/educational/NHS/ private/academic roles, estimate how many COI forms they estimate they fill out per year

- location (UK or not)

When we ask for age bands, under 18s will not be included.

-we will ask participants about what they understand by declared interests/ conflicts and how they interpret them. We will use vignettes. For ease of analysis, these will be composed of questions answered by tick box design.

- we will ask how long they think it should take to find out a doctors declaration of interest

- to search for declarations in 10 minutes, using a timer on the website

- at the end of the time we will ask them to report what they find

- we will then compare the declarations 'gold standard" which we have previously listed to participants findings. Disagreement will be resolved

through discussion. The researchers will be blinded to the age, sex, professional background and email address of the participant (if applicable).

- we will then analyse the information. We will assess a) the frequency of matching of gold standard declarations/conflicts against 1) our findings 2) the findings of the participants. Because these will vary in amount, we will express the overlap in percentage terms.

- we will ask permission to contact via email for participation in interview based qualitative research at a later date. Email addresses which are not associated with a wish to be notified of the results/opportunities will be destroyed at the end of the research period.

-We will run this methodology as a pilot with small amounts of diverse participants, to refine questions, website layout, ease of access, and with feedback. We will then roll it out as planned to a wider audience.

We will contact a representative sample of the participants who have indicated that they would be willing to be interviewed. We will aim for diversity in age, sex, and professional role, and in people with divergent answers in the questionnaires.

We will proceed to interview these participants remotely with experienced qualitative researchers. We will aim to understand what people view a conflict as, how these should be managed, and therefore what factors should be accounted for when policies are made on this. We will use semi-structured interviews, with the 'think aloud' method in order to draw themes which will then be analysed.
No files selected
Randomization
No response

Sampling Plan
Existing Data
Registration prior to creation of data
Explanation of existing data
n/a
Data collection procedures
There are three groups of participants.
Conflicts of interest subjects: 12-15 health professionals who have given recent advice or information to citizens in the public domain and who therefore may have come to the attention of the public. These will be a mixture of healthcare officials (for example, CMOs or deputy CMOs) heads of

royal colleges, professional societies, pressure groups, NICE and SIGN guideline authors, and doc-tor journalists/columnists.
Citizens (COI participants) - via invitations through social media, and citizens organisations; patients - via campaigning organisations such as Sling the Mesh, with invitations to organisations including The Patient Association, National Voices, social media. Healthcare students - via requests to university and college mailing lists for dissemination, social media. Higher specialist trainees - via social media, and requests to disseminate via Royal Colleges and NHS Trusts. The Medical Schools Council have been asked for permission to contact medical students subject to ethics approval and contact will not otherwise proceed without it.
Healthcare professionals - via requests to GP practices via Scottish Primary Care Research Network, social media and professional forums. The healthcare professionals we ask to take part as subjects will be based pragmatically on well known doctors and approached individually.
Qualitative research group: a representative sample of the citizen group, likely to be around 30 participants.

No files selected

Sample size

For the citizen group (COI participants) o have a confidence interval of 95% with a 5% margin, with a proportion of 0.5, we need a sample size of 385 citizens (out of a UK population of 66 million). By asking for the professional / patient / citizen identity of participants, we will draw conclusions about groups if the sample size allows.

Sample size rationale

The number of COI subjects is relatively small as each requires the creation of a 'gold standard' COI statement for comparison to be made. Larger samples would require excessive resources for little benefit.

Stopping rule

We will be time limited and aim to have enough participants to achieve the sample size within 4 weeks.

Variables

Manipulated variables

n/a

No files selected

Measured variables

1) Outcomes of vignettes (percieved trust with declared interests)
2) Comparison of participant searches with our 'gold standard' searches
3) Agreement of research searches with COI-subject declarations

Narrative analysis of qualitative interviews

No files selected

Indices

n/a

No files selected

Analysis Plan

Statistical models

Simple tabulation for analysis of responses to vignettes.
Tabulation with percentage agreement for COI searches.

We will perform a thematic analysis of the qualitative interviews.

No files selected

Transformations

n/a

Inference criteria

n/a

Data exclusion

n/a

Missing data

We will include partial data with an explanatory note.

Exploratory analysis

n/a

Other

Other

The Cumberlege review (2020) investigated the harms caused by surgical mesh to patients. It concluded that serious problems are caused by a lack of transparency of conflicts of interest (1). They found that surgical mesh was used and promoted by clinicians with financial interests in the product, even though it was inadequately tested. The review recommended that doctors should make statutory disclosures of interests. Such interests would include, for example, consultancy payments from pharmaceutical companies, patents for technological devices, or shareholding in device companies. The General Medical Council already recommends that doctors are open and transparent about their interests. Previous enquiries, for example, the Health Select Committee in the 2005 report 'The Influence of the Pharmaceutical Industry', recommended that all healthcare professionals make statutory declarations of interest to their regulator (2). This has not occurred.

This is a relatively small, emerging field of research. Key references were sought via Cochrane, PubMed, and 'snowball' searching of reference lists. There is evidence of widespread harm to patients and healthcare systems because of conflicts of interest. For example, guidelines dealing with opioid

prescribing for non-cancer pain (recognised as driving ‘the opioid crisis’) have had a “pervasive presence” of influence of the pharmaceutical industry amongst authors or organisations recieving funding from them (3). Doctors with financial conflicts of interest are more likely to have favourable views on the side effects from medications (4). Doctors receiving gifts and education from pharmaceutical sales representatives have poorer quality prescribing, and believe their peers, but not themselves, are adversely affected by conflicts(5).

Many countries mandate disclosures of interest by health professionals (6). The impact of this is unclear. There are concerns that statutory declarations, rather than reducing the negative impact of conflicts of interest, may transfer the burden of searching for, interpreting, and acting on them to patients (7). The US Sunshine Act does not appear to have improved patient knowledge of physician payments, with less than 5% of people knowing whether their own doctor has received industry payments (8). Further, there are concerns that disclosing interests may increase bias through ‘moral license’, when doctors believe that disclosure equals management of conflicts, and where patients and citizens believe that transparency negates bias (9). In the US, patients who are explicitly told about a doctor’s financial conflicts with industry do not appear to change their attendance with the doctor, with no loss of trust (10). US patients at cancer centres have low levels of concern about doctors financial conflicts from pharmaceutical companies (11). The UK has a voluntary system, Disclosure UK, where payments to professionals from pharmaceutical companies are published annually, but most money is undeclared (12). NHS Trusts in England mandate disclosures for staff but these are incomplete and of poor quality (13). An up to date evaluation of current practice is currently being made by MM and others, early findings indicate widespread poor quality declarations.

As the NHS considers its’ formal response to the Cumberlege report (14), potential actions in the recording and managing of declarations of interest require consideration. Little is known about how much knowledge patients, citizens, professionals and policymakers have regarding conflicts of interests in healthcare professionals, or locating and interpreting this information. A 2016 systematic review on knowledge, beliefs and attitudes of patients and the public towards interactions between professionals and the pharmaceutical and device industry found low levels of concern about financial conflicts but did not include any studies from the UK or Europe, with the exception of Turkey (15).

There is a large gap in our understanding of the most effective way to declare and interpret conflicts of interest. This is required in order to increase

public and professional understanding of their impacts and ultimately reduce the impact of bias in healthcare and improve quality.

1. Independent Medicines and Medical Devices Review. First do no harm. 2020. https://www.immdsre-

2. The influence of the Pharmaceutical Industry. 2014/5 session Health Select Committee UK Parliament

3. Spithoff S, Leece P, Sullivan F, Persaud N, Belesiotis P, Steiner L. Drivers of the opioid crisis: An appraisal of financial conflicts of interest in clinical practice guideline panels at the peak of opioid prescribing. PLoS One. 2020;15(1):e0227045. Published 2020 Jan 24. doi:10.1371/journal.pone.0227045

4. Wang AT, McCoy CP, Murad MH, Montori VM. Association between industry affiliation and position on cardio-vascular risk with rosiglitazone: cross sectional systematic review. BMJ. 2010;340: c1344–c1344. 10.1136/bmj.c1344

5. Fickweiler F, Fickweiler W, Urbach E. Interactions between physicians and the pharmaceutical industry gen-erally and sales representatives specifically and their association with physicians' attitudes and prescribing habits: a systematic review. BMJ Open. 2017;7(9):e016408. Published 2017 Sep 27. doi:10.1136/bmjopen-2017-016408

6. The sun shines on Europe. Transparency of financial relationships in the healthcare sector. 2017 Health Ac-tion International https://haiweb.org/wp-content/uploads/2017/03/Sunshine-Act.pdf

7. Wilson M. (2014). The Sunshine Act: commercial conflicts of interest and the limits of transparency. Open medicine : a peer-reviewed, independent, open-access journal, 8(1), e10–e13.

8. Kanter GP, Carpenter D, Lehmann L, et al Effect of the public disclosure of industry payments information on patients: results from a population-based natural experiment BMJ Open 2019;9:e024020. doi: 10.1136/bmjopen-2018-024020

9. Does Conflict of Interest Disclosure Worsen Bias? PLOS Medicine 24/4/12 https://doi.org/10.1371/jour-

10. Susannah L. Rose, Sunita Sah, Raed Dweik, Cory Schmidt, MaryBeth Mercer, Ariane Mitchum, Michael Kattan, Matthew Karafa, Christopher Robertson, Patient responses to physician disclosures of industry con-flicts

of interest: A randomized field experiment, Organizational Behavior and Human Decision Processes, 2019, ISSN 0749-5978 https://doi.org/10.1016/j.obhdp.2019.03.005.

11. Hampson et al. Patients' views on financial conflicts of interest. N Engl J Med 2006; 355:2330-2337 DOI: 10.1056/NEJMsa064160

12. Kmietowicz Zosia. Disclosure UK website gives "illusion of transparency," says Goldacre BMJ 2016; 354 :i3760

13. Feldman HR, DeVito NJ, Mendel J, et alA cross-sectional study of all clinicians' conflict of interest dis-closures to NHS hospital employers in England 2015-2016BMJ Open 2018;8:e019952. doi: 10.1136/bmjopen-2017-019952

14. Macdonald Helen, McCartney Margaret, Heneghan Carl, Godlee Fiona. Doctors' conflicts of interest BMJ 2020; 370 :m3247

15. Fadlallah R et al. Knowledge, Beliefs and Attitudes of Patients and the General Public towards the inter-actions of Physicians and the Pharmaceutical and Device Industry: a systematic review. PLOS 24/8/1

Standards for Reporting Qualitative Research (SRQR)*

http://www.equator-network.org/reporting-guidelines/srqr/

Page/line no(s).

Title and abstract

**Title** - Concise description of the nature and topic of the study Identifying the study as qualitative or indicating the approach (e.g., ethnography, grounded theory) or data collection methods (e.g., interview, focus group) is recommended	page 2
**Abstract** - Summary of key elements of the study using the abstract format of the intended publication; typically includes background, purpose, methods, results, and conclusions	page 1

Introduction

**Problem formulation** - Description and significance of the problem/phenomenon studied; review of relevant theory and empirical work; problem statement	Page 1
**Purpose or research questio**n - Purpose of the study and specific objectives or questions	Page 1

Methods

**Qualitative approach and research paradigm** - Qualitative approach (e.g., ethnography, grounded theory, case study, phenomenology, narrative research) and guiding theory if appropriate; identifying the research paradigm (e.g., postpositivist, constructivist/ interpretivist) is also recommended; rationale**	page 3
**Researcher characteristics and reflexivity** - Researchers' characteristics that may influence the research, including personal attributes, qualifications/experience, relationship with participants, assumptions, and/or presuppositions; potential or actual interaction between researchers' characteristics and the research questions, approach, methods, results, and/or transferability	page 1 (under COIs)
**Context** - Setting/site and salient contextual factors; rationale**	page 1
**Sampling strategy** - How and why research participants, documents, or events were selected; criteria for deciding when no further sampling was necessary (e.g., sampling saturation); rationale**	page 4
**Ethical issues pertaining to human subjects** - Documentation of approval by an appropriate ethics review board and participant consent, or explanation for lack thereof; other confidentiality and data security issues	page 2

For peer review only - http://bmjopen.bmj.com/site/about/guidelines.xhtml

For peer review only

**Data collection methods** - Types of data collected; details of data collection procedures including (as appropriate) start and stop dates of data collection and analysis, iterative process, triangulation of sources/methods, and modification of procedures in response to evolving study findings; rationale**	page 5
**Data collection instruments and technologies** - Description of instruments (e.g., interview guides, questionnaires) and devices (e.g., audio recorders) used for data collection; if/how the instrument(s) changed over the course of the study	page 5
**Units of study** - Number and relevant characteristics of participants, documents, or events included in the study; level of participation (could be reported in results)	page 6, Table 1
**Data processing** - Methods for processing data prior to and during analysis, including transcription, data entry, data management and security, verification of data integrity, data coding, and anonymization/de-identification of excerpts	page 6
**Data analysis** - Process by which inferences, themes, etc., were identified and developed, including the researchers involved in data analysis; usually references a specific paradigm or approach; rationale**	page 8
**Techniques to enhance trustworthiness** - Techniques to enhance trustworthiness and credibility of data analysis (e.g., member checking, audit trail, triangulation); rationale**	page 8

Results/findings

**Synthesis and interpretation** - Main findings (e.g., interpretations, inferences, and themes); might include development of a theory or model, or integration with prior research or theory	page 8/9
**Links to empirical data** - Evidence (e.g., quotes, field notes, text excerpts, photographs) to substantiate analytic findings	page 8/9, appendix 9

Discussion

**Integration with prior work, implications, transferability, and contribution(s) to the field -** Short summary of main findings; explanation of how findings and conclusions connect to, support, elaborate on, or challenge conclusions of earlier scholarship; discussion of scope of application/generalizability; identification of unique contribution(s) to scholarship in a discipline or field	page 10,11,12
**Limitations** - Trustworthiness and limitations of findings	page 13

Other

**Conflicts of interest** - Potential sources of influence or perceived influence on study conduct and conclusions; how these were managed	page 2

For peer review only - http://bmjopen.bmj.com/site/about/guidelines.xhtml

**Funding** - Sources of funding and other support; role of funders in data collection, interpretation, and reporting	page 2

*The authors created the SRQR by searching the literature to identify guidelines, reporting standards, and critical appraisal criteria for qualitative research; reviewing the reference lists of retrieved sources; and contacting experts to gain feedback. The SRQR aims to improve the transparency of all aspects of qualitative research by providing clear standards for reporting qualitative research.

**The rationale should briefly discuss the justification for choosing that theory, approach, method, or technique rather than other options available, the assumptions and limitations implicit in those choices, and how those choices influence study conclusions and transferability. As appropriate, the rationale for several items might be discussed together.

Reference:

O'Brien BC, Harris IB, Beckman TJ, Reed DA, Cook DA. **Standards for reporting qualitative research: a synthesis of recommendations.** *Academic Medicine*, Vol. 89, No. 9 / Sept 2014 DOI: 10.1097/ACM.0000000000000388

For peer review only

Checklist for Reporting Of Survey Studies (CROSS)

Section/topic	Item	Item description	Reported on page #
**Title and abstract**			
Title and abstract	1a	State the word "survey" along with a commonly used term in title or abstract to introduce the study's design.	included in 'mixed method'
	1b	Provide an informative summary in the abstract, covering background, objectives, methods, findings/results, interpretation/discussion, and conclusions.	page 1
**Introduction**			
Background	2	Provide a background about the rationale of study, what has been previously done, and why this survey is needed.	page 3
Purpose/aim	3	Identify specific purposes, aims, goals, or objectives of the study.	
**Methods**			
Study design	4	Specify the study design in the methods section with a commonly used term (e.g., cross-sectional or longitudinal).	page 1
Data collection methods	5a	Describe the questionnaire (e.g., number of sections, number of questions, number and names of instruments used).	page 4
	5b	Describe all questionnaire instruments that were used in the survey to measure particular concepts. Report target population, reported validity and reliability information, scoring/classification procedure, and reference links (if any).	page 4/appendix 2,3,4,5,6
	5c	Provide information on pretesting of the questionnaire, if performed (in the article or in an online supplement). Report the method of pretesting, number of times questionnaire was pre-tested, number and demographics of participants used for pretesting, and the level of similarity of demographics between pre-testing	page 5

For peer review only

		participants and sample population.	
	5d	Questionnaire if possible, should be fully provided (in the article, or as appendices or as an online supplement).	appendix 3-6
Sample characteristics	6a	Describe the study population (i.e., background, locations, eligibility criteria for participant inclusion in survey, exclusion criteria).	page 6
	6b	Describe the sampling techniques used (e.g., single stage or multistage sampling, simple random sampling, stratified sampling, cluster sampling, convenience sampling). Specify the locations of sample participants whenever clustered sampling was applied.	page 6
	6c	Provide information on sample size, along with details of sample size calculation.	page 6
	6d	Describe how representative the sample is of the study population (or target population if possible), particularly for population-based surveys.	page 6
Survey administration	7a	Provide information on modes of questionnaire administration, including the type and number of contacts, the location where the survey was conducted (e.g., outpatient room or by use of online tools, such as SurveyMonkey).	page 4
	7b	Provide information of survey's time frame, such as periods of recruitment, exposure, and follow-up days.	protocol, appendix 11, and protocol deviation page 16.
	7c	Provide information on the entry process:<br>–>For non-web-based surveys, provide approaches to minimize human error in data entry.<br>–>For web-based surveys, provide approaches to prevent "multiple participation" of participants.	discussed on page 14
Study preparation	8	Describe any preparation process before conducting the survey (e.g., interviewers' training process, advertising the survey).	n/a
Ethical considerations	9a	Provide information on ethical approval for the survey if obtained, including informed consent, institutional review board [IRB] approval, Helsinki declaration, and good clinical practice [GCP] declaration (as appropriate).	page 2

For peer review only

	9b	Provide information about survey anonymity and confidentiality and describe what mechanisms were used to protect unauthorized access.	page 2 (inherent in ethical approvals)
Statistical analysis	10 a	Describe statistical methods and analytical approach. Report the statistical software that was used for data analysis.	n/a simple analysis only
	10 b	Report any modification of variables used in the analysis, along with reference (if available).	n/a
	10 c	Report details about how missing data was handled. Include rate of missing items, missing data mechanism (i.e., missing completely at random [MCAR], missing at random [MAR] or missing not at random [MNAR]) and methods used to deal with missing data (e.g., multiple imputation).	Survey breakdown of responses in results, pages 6/7
	10 d	State how non-response error was addressed.	
	10 e	For longitudinal surveys, state how loss to follow-up was addressed.	
	10f	Indicate whether any methods such as weighting of items or propensity scores have been used to adjust for non-representativeness of the sample.	
	10 g	Describe any sensitivity analysis conducted.	
**Results**			
Respondent characteristics	11 a	Report numbers of individuals at each stage of the study. Consider using a flow diagram, if possible.	page 6/7
	11 b	Provide reasons for non-participation at each stage, if possible.	page 7, 13
	11	Report response rate, present the definition of response rate or the formula used to	n/a

For peer review only

	c	calculate response rate.	
	11 d	Provide information to define how unique visitors are determined. Report number of unique visitors along with relevant proportions (e.g., view proportion, participation proportion, completion proportion).	n/a (see page 6/7)
Descriptive results	12	Provide characteristics of study participants, as well as information on potential confounders and assessed outcomes.	page 6/7
Main findings	13 a	Give unadjusted estimates and, if applicable, confounder-adjusted estimates along with 95% confidence intervals and p-values.	n./a
	13 b	For multivariable analysis, provide information on the model building process, model fit statistics, and model assumptions (as appropriate).	n/a
	13 c	Provide details about any sensitivity analysis performed. If there are considerable amount of missing data, report sensitivity analyses comparing the results of complete cases with that of the imputed dataset (if possible).	n/a
**Discussion**			
Limitations	14	Discuss the limitations of the study, considering sources of potential biases and imprecisions, such as non-representativeness of sample, study design, important uncontrolled confounders.	page 16
Interpretations	15	Give a cautious overall interpretation of results, based on potential biases and imprecisions and suggest areas for future research.	page 16/17
Generalizability	16	Discuss the external validity of the results.	
**Other sections**			
Role of funding source	17	State whether any funding organization has had any roles in the survey's design, implementation, and analysis.	n/a
Conflict of interest	18	Declare any potential conflict of interest.	page 2
Acknowledgements	19	Provide names of organizations/persons that are acknowledged along with their contribution to the research.	page 2, 16

For peer review only

For peer review only

BMJ Open

You feel like you've been duped". Is the current system for health professionals declaring potential conflicts of interest in the UK fit for purpose? A mixed methods study.

Journal:	*BMJ Open*
Manuscript ID	bmjopen-2023-072996.R1
Article Type:	Original research
Date Submitted by the Author:	09-May-2023
Complete List of Authors:	McCartney, Margaret; University of St Andrews,<br>Metsis, Katrin; University of St Andrews, School of Medicine<br>MacDonald, Ronald; University of St Andrews School of Medicine, ; University of Dundee School of Medicine,<br>Sullivan, Frank; University of St. Andrews, ; North York General hospital,<br><br>Ozakinci, Gozde; University of St Andrews<br>Boylan, Anne-Marie; Oxford University, Nuffield Department of Primary Care Health Sciences
**Primary Subject Heading**:	Health services research
Secondary Subject Heading:	Evidence based practice
Keywords:	MEDICAL ETHICS, Information management < BIOTECHNOLOGY & BIOINFORMATICS, HEALTH SERVICES ADMINISTRATION & MANAGEMENT, Health policy < HEALTH SERVICES ADMINISTRATION & MANAGEMENT, Protocols & guidelines < HEALTH SERVICES ADMINISTRATION & MANAGEMENT, Patient-Centered Care

SCHOLARONE™
Manuscripts

I, the Submitting Author has the right to grant and does grant on behalf of all authors of the Work (as defined in the below author licence), an exclusive licence and/or a non-exclusive licence for contributions from authors who are: i) UK Crown employees; ii) where BMJ has agreed a CC-BY licence shall apply, and/or iii) in accordance with the terms applicable for US Federal Government officers or employees acting as part of their official duties; on a worldwide, perpetual, irrevocable, royalty-free basis to BMJ Publishing Group Ltd ("BMJ") its licensees and where the relevant Journal is co-owned by BMJ to the co-owners of the Journal, to publish the Work in this journal and any other BMJ products and to exploit all rights, as set out in our licence.

The Submitting Author accepts and understands that any supply made under these terms is made by BMJ to the Submitting Author unless you are acting as an employee on behalf of your employer or a postgraduate student of an affiliated institution which is paying any applicable article publishing charge ("APC") for Open Access articles. Where the Submitting Author wishes to make the Work available on an Open Access basis (and intends to pay the relevant APC), the terms of reuse of such Open Access shall be governed by a Creative Commons licence – details of these licences and which Creative Commons licence will apply to this Work are set out in our licence referred to above.

Other than as permitted in any relevant BMJ Author's Self Archiving Policies, I confirm this Work has not been accepted for publication elsewhere, is not being considered for publication elsewhere and does not duplicate material already published. I confirm all authors consent to publication of this Work and authorise the granting of this licence.

For peer review only

You feel like you've been duped". Is the current system for health professionals declaring potential conflicts of interest in the UK fit for purpose? A mixed methods study.

Margaret McCartney, Honorary Senior Lecturer* (1), Katrin Metsis, Research Fellow (1), Ronald MacDonald, medical student (1), Frank Sullivan, Director of Research (1), Gozde Ozakinci, Professor of Health Psychology (2), Anne-Marie Boylan, Senior Research Fellow (3)

1) School of Medicine, University of St Andrews, North Haugh, St Andrews, Fife, UK KY16 9TF 2) University of Stirling, Stirling, UK FK9 4LA 3) Nuffield Department of Primary Care Health Sciences, Oxford University, OX2 6GG.

*Corresponding author
mm494@st-andrews.ac.uk

For peer review only

Abstract:

Objective: To understand: if professionals, citizens and patients can locate UK healthcare professionals' statements of declarations of interests, and what citizens understand by these.

Design: The study sample included two groups of participants in three phases. First, healthcare professionals working in the public domain (health professional participants, HPP) were invited to participate. Their conflicts and declarations of interest were searched for in publicly available data, which the HPP checked and confirmed as the 'gold standard'. In the second phase, laypeople, other healthcare professionals and healthcare students were invited to complete three online tasks. The first task was a questionnaire about their own demographics. The second task was questions about doctors conflicts of interest in clinical vignette scenarios. The third task was a request for each participant to locate and describe the declarations of interest of one of the named healthcare professionals identified in the first phase, randomly assigned. At the end of this task, all lay participants were asked to indicate willingness to be interviewed at a later date. In the third phase, each lay respondent who was willing to be contacted was invited to a qualitative interview to obtain their views on the conflicts and declaration of interest they found and their meaning.

Setting: Online, based in the UK.

Participants: 13 public-facing health professionals, 379 participants (healthcare professionals, students and laypeople), 21 lay interviewees.

Outcome measures: 1) Participants' level of trust in professionals with variable conflicts of interest, as expressed in vignettes, 2) participants' ability to locate the declarations of interest of a given well-known healthcare professional, 3) laypeople's understanding of healthcare professionals declarations and conflicts of interest.

Results: In the first phase, 13 health professionals (HPP) participated and agreed a 'gold standard' of their declarations. In the second phase, 379 citizens, patients, other healthcare professionals and students participated. Not all completed all aspects of the research. 85% of participants thought that knowing about professional declarations was definitely or probably important, but 76.8% were not confident they had found all relevant information after searching. As conflicts of interest increased in the vignettes, participants trusted doctors less. Least trust was associated with doctors who had not disclosed their conflicts of interest. 297 participants agreed to search for HPP 'gold standard' declaration of interest, and 169 reported some data. Of

those reporting any findings, 61 (36%) located a relevant link to some information deemed fit for purpose, and 5 (3%) of participants found all the information contained in the 'gold standard'. In the third phase, qualitative interviews with 21 participants highlighted the importance of transparency but raised serious concerns about how useful declarations were in their current format, and whether they could improve patient care. Unintended consequences, such as the burden for patients and professionals to use declarations were identified, with participants additionally expressing concerns about professional bias and a lack of insight over conflicts. Suggestions for improvements included better regulation and organisation, but also second opinions and independent advice where conflicts of interest were suspected.

Conclusion: Declarations of interest are important and conflicts of interest concern patients and professionals, particularly in regard to trust in decision making. If declarations, as currently made, are intended to improve transparency, they do not achieve this, due to difficulties in locating and interpreting them. Unintended consequences may arise if transparency alone is assumed to provide management of conflicts. Increased trust resulting from transparency may be misplaced, given the evidence on the hazards associated with conflicts of interest. Clarity about the purposes of transparency is required. Future policies may be more successful if focussed on reducing the potential for negative impacts of conflicts of interest, rather than relying on individuals to locate declarations and interpret them.

Registration: The protocol was pre-registered at https://osf.io/e7gtq.

Ethics: This project was approved by the University of St Andrews School of Medicine Ethics Committee, MD16045.

Strengths and Limitations of the study:

- This is the first study we are aware of to test whether professionals and laypeople can find and interpret declarations of interest made by professionals in the UK
- Co-designed with a patient panel
- Pragmatic, real-world study design

- Participants were likely to be atypical and not representative of most professionals or patients
- Success in finding declarations was judged if some, not all declarations were found, overestimating the availability of declarations
- Declarations are not of equal importance but were graded equally for the purposes of analysis

Introduction:

Contention has surrounded the definition, declaration, and management of interests in medical practice for decades. When the US Congress asked 'Is science for sale?' in a series of hearings regarding research fraud in the late 1990s, declarations of interest were generally haphazard and voluntary. In 2009, The International Committee of Medical Journal Editors produced a standardised template for recording and publishing declarations, which has been serially upgraded since (1). However, declaring interests has subsequently become an expectation in routine clinical practice, not just academic publishing, and is now law via Sunshine Acts for healthcare professionals in several countries. In NHS England, guidance published in 2017 stipulates how and which declarations should be publicly made (2).

There is evidence of widespread harm to patients and healthcare systems because of financial conflicts of interest. For example, guidelines dealing with opioid prescribing for non-cancer pain (recognised as driving 'the opioid crisis') have had a "pervasive presence" of influence of the pharmaceutical industry amongst guideline authors or organisations receiving funding from them (3). Doctors with financial conflicts of interest are more likely to have favourable views on side effects from medications (4). Meanwhile, doctors receiving gifts and education from pharmaceutical sales representatives have poorer quality prescribing, and believe their peers, but not themselves, are adversely affected by conflicts (5).

For peer review only - http://bmjopen.bmj.com/site/about/guidelines.xhtml

Healthcare professionals are often asked to make declarations, or "declarations of interest" (DOI) in different venues, for example, workplaces, academic journals, and conference presentations. Such declarations relate to the act of recording any interests which may be, or could potentially become a conflict, depending on context or circumstances. A conflict of interest, however, is "a set of circumstances that create a risk that an individual's ability to apply judgement or act in one role is, or could be, impaired or influenced by a secondary interest." (6). The UK government commissioned the Independent Medicines and Medical Devices Safety Review (IMMDS) in 2020, to investigate how the health service responded to concerns about medical products, and one conclusion was that serious problems are caused by a lack of transparency of conflicts of interest (COI) (7). However, there may be uncertainty over when a declaration represents a conflict, particularly when a declaration is prepared in advance and used for multiple purposes. The review recommended that doctors should make statutory disclosures of interests on a central register, including, for example, consultancy payments from pharmaceutical companies, patents for technological devices, or shareholdings in device companies. The General Medical Council already recommends that doctors are open and transparent about their interests. Previous inquiries, for example, the Health Select Committee in the 2005 report 'The Influence of the Pharmaceutical Industry', recommended that all healthcare professionals make statutory declarations of interest to their regulator (8), but this did not occur.

There are multiple types of potential conflicts of interest. NHS England classes these as financial or non-financial. The first is where an individual receives or may receive a direct financial benefit from the consequences of the awarding of funding (2). This can be direct (for example, a shareholder receiving more funds for increased sales in a product) or indirect (where a person closely associated, such as a spouse or business partner will benefit similarly). This may also include industry funding to attend conferences, to advise or provide consultancy, fees for speaking, or research funding. Non-financial interests can be professional (e.g. a decision likely to enhance a career or status, or an intellectual bias) or non-professional (where other interests, such being a member of a lobby group, may compete).

Many countries mandate disclosures of interest by health professionals (9), The US Sunshine Act, which mandates transparency of payments from industry, was implemented in 2013. The same year, a Disclosure Code by the European Federation of Pharmaceutical Industries and Associations (EFPIA) was implemented to improve transparency, but is variable by country where it conflicts with other laws (10). In the US, the Act does not seem to have resulted in improved patient knowledge of physician payments, with less than 5% of people knowing whether their own doctor has received industry payments (11). Declarations of interest may be put to multiple purposes. For example, in guideline committees (12), where individuals may only be allowed limited roles or excluded entirely on the basis of conflicts. Regulators may also stipulate specific conflicts which must be declared to patients (13). Healthcare professionals may also use conflicts of interest statements to make judgements about the validity of opinions or published research findings. Managers may have to use declarations to ensure that commissioning or procurement decisions are made by non-conflicted individuals. The use of declarations by patients has been emphasised in the IMMDS review, which has called for patients to be able to access declarations in order to "reach informed decisions about who is best to treat us" (7). There are therefore multiple purposes to which declarations can be put.

Disclosures of interest have been historically acknowledged as necessary but also with multiple inadequacies in practice, including poor quality recording and subsequent management (14). There are additional concerns that statutory declarations, rather than reducing the negative impact of conflicts of interest, may transfer the burden of searching for, interpreting, and acting on them to patients (15). Further, there are concerns that disclosing interests may increase bias through 'moral licence', when doctors believe that disclosure equals management of conflicts, and where patients and citizens believe that transparency negates bias (16). In the US, patients who are explicitly told about a doctor's financial conflicts with industry do not appear to change their attendance with

For peer review only

the doctor, with no loss of trust (17); however other research suggests that disclosure may result in an increase in trust if it is regarded as a sign of honesty (18). Patients at US cancer centres have low levels of concern about doctors' financial conflicts from pharmaceutical companies (19); however, this study did not ask patients what they thought the negative impacts might be. The UK has a voluntary system, Disclosure UK, where payments to professionals from pharmaceutical companies are published annually, but most money is undeclared (20). NHS Trusts in England mandate disclosures for staff but these are incomplete and of poor quality (21). Therefore, while voluntary and mandated disclosures from healthcare staff are available to patients in the UK, these are problematic due to their quality, and it is unknown whether these incur unintended hazards from disclosures, and whether they enable evidence-informed decision making.

As the NHS responds to the Cumberlege report (22), potential actions in the recording and managing of declarations of interest require consideration. Little is known about how much knowledge patients, citizens, professionals and policymakers have regarding conflicts of interests among healthcare professionals, or locating and interpreting this information. A 2016 systematic review on knowledge, beliefs and attitudes of patients and the public towards interactions between professionals and the pharmaceutical and device industry found low levels of concern about financial conflicts but did not include any studies from the UK or Europe, with the exception of Turkey (23).

There is a large gap in our understanding of the most effective way to declare and interpret interests, and what patients, citizens and professionals think about the impact of them. This is required in order to ensure that any change in policies are beneficial and meet their intended purpose. The aim of the study was to understand: if professionals, citizens and patients can locate UK healthcare professionals' statements of declarations of interests, and what citizens understand by these.

Methods:

Patient involvement: We thank the lay group for their advice before, during and after the study. They helped to design the methods, the questionnaires, the choice of professional participants, and assisted in recruiting lay participants. The results will be shared with them.

This research takes a pragmatist perspective (24, 25). While the different epistemological standpoints of qualitative and quantitative methodologies are acknowledged, a mixed methods approach was chosen to gain a comprehensive understanding of the citizen and patient participants' perspectives of COIs in health professionals. The online survey, completed in the second phase, provides a quantified understanding of the respondents' perceptions of health professionals' variable interests and the process of locating declarations. Qualitative interviews in the third phase investigates lay perspectives of declarations and conflicts of interest in healthcare, and current declaration strategies. Thus, quantitative and qualitative data are used to gain an understanding of the different aspects of the phenomenon, which are integrated but keeping their epistemological differences (26, 27, 28).

Phase 1: Methods development

The research team established a PPI group. This consisted of 4 lay people who are involved with patient representation at the UK Royal College of General Practitioners or who have been involved in lay activism regarding surgical mesh. The PPI group helped design the methods, the questionnaires, the choice of professional participants, and assisted in recruiting lay participants.

Professionals (HPP, Health Professional Participants) were identified and invited on the basis of their recent history of giving medical advice or information to the public, where a citizen might reasonably wish to know their declarations of interest. A mixture of healthcare officials based in the UK (for example, doctors employed by government), heads of royal colleges/professional societies, pressure groups, NICE and SIGN guideline authors, and doctor journalists were invited. This was done in batches of 10-15 aiming to recruit 12-15 in total. A standard process was used to search for the professionals' conflicts of interest

For peer review only

(Appendix 1). Each professional was asked to check the findings and approve or disagree with them. This formed the ‘gold standard’. Statements within each ‘gold standard’ declaration were divided into ‘financial professional’, ‘non-financial professional’ ‘personal’ and ‘indirect’ as per the NHS England disclosure framework (2). This recruitment of healthcare professional participants (HPP) completed the first phase of the study.

Phase 2: Online survey

An online survey (using Qualtrics) was developed and tested with the lay group (supplement). This contained 4 scenarios explaining and asking opinions on potential financial conflicts of interest. We described these to participants as ‘interests’ and not ‘conflicts’. A wide range of participants from citizen, patient, activist, professional and student groups were formally invited (Appendix 2) to participate online, with patient and professional networks asked to publicise the research via social media. The invitation contained a web link to the survey which prospective participants could click on. Demographic and occupational information was requested. Healthcare professionals were additionally asked how many declaration of interest forms they were asked to complete a year. Participants were then asked to express their trust in a doctor, and whether they felt they were acting in their best interests, within a vignette about a surgical procedure, where doctors making a recommendation had variable but increasing financial interests. These were either not present, present and declared, or not declared but found by the participant. This was designed to give information about what a declaration of interest is, to gauge views on their importance in terms of impact on personal decision making, and to explain and prime participants as to what a declaration of interest was before being asked to locate them (Appendix 3). These used 5 point Likert scales.

The participants were then randomly assigned to one HPP from the group of 13, with a brief introduction about the person, and asked to spend around 10 minutes (as suggested by the PPI group) to search for and report their declarations of interest. However, participants could spend as little or as much time as wished. We explained that we were not seeking any ‘personal’ information such as age or marital status or address but either a statement of declarations of interests, or the interests they found. Information on how long participants felt reasonable to search for a conflict was also sought.

They were asked to report findings in a web form. Excel was used to tabulate each piece of data reported by participants. These were compared to the professional’s ‘gold standard’, which were divided into financial and non-financial declarations. Each was deemed ‘significant’ or ‘non significant’ (Appendix 4) based on NHS England criteria (2).

Participants’ responses to the HPP ‘gold standard’ were analysed by hand. Participants were asked to record all information found, supplying relevant web links. The responses were recorded and assessed for accuracy and completeness via comparison with the ‘gold standard’ (MM, RM). Given the difficulty the task was expected to pose, marking was generous. We did not ask either the professionals or the participants to categorise interests within each NHS England category of declarations (e.g. financial, non-financial professional, non-financial personal interests, and indirect interests) but the research team categorised and marked them on this basis. If a ‘gold standard’ declaration containing full details was not available online for participants to locate, locating a single declaration from each category of declaration was marked as successful, even if incomplete. 10% of the results were checked by the second researcher for accuracy and no disagreements were found. Null declarations were excluded in the tally (ie the denominator was according to the declarations present). This strategy would have overestimated the tally of declarations found, by design. At the end of the survey, all lay people were asked if they would like to take part in a qualitative interview at a later stage.

For peer review only

Third phase: interviews

In the third phase, all lay participants who stated they were willing to take part in an interview were contacted. This was a semi-structured, qualitative interview where their views about both declarations of interest and where these represented conflicts could be explored. 50 agreed to be contacted and of these, 21 consented to be interviewed (KM, MM) (Appendix Table 1). A semi-structured interview format was chosen as this method is well suited for the exploration of opinions and perceptions, enabling the further exploration of the topics identified by respondents (29). A topic guide was developed for use in the interviews with the understanding that it would be iteratively refined and new questions added as data collection progressed. Questions included exploring what declarations of interest were, and their purpose, what a 'conflict of interest' in healthcare professionals means, what was known about them, if they were perceived as important, and how they should be managed. The initial topics were developed after discussion with the lay group and related to real-life practice in the UK, where the medical regulator advises that professionals should use self-judgement to decide when a conflict occurs and when it should be declared (13). All interviews were transcribed verbatim and transcripts were transferred to NVivo for analysis.

Interview data was analysed using thematic analysis (30). KM completed 20 out of 21 interviews (MM did one). KM created the initial set of codes based on six transcripts. MM read and discussed all transcripts, interview notes, and initial codes. This was then discussed with AMB alongside, with conceptualisation of potential themes. Next, all transcripts were coded in NVivo and initial themes were developed by KM. Initial themes were further developed and refined with input from MM and AMB. KM, MM and AMB developed the final three themes and these were agreed by the whole team.

Results:

Phase 1:

A total of 65 professionals were invited; 3 sent a formal declination and 13 consented to take part. Two did not respond after further information was requested and sent; the remainder did not reply. The research team completed the 'gold standard' and presented it to the HPP. All but one statement was agreed for one HPP. After discussion it was deemed inaccurate by both the professional involved and the research team. No information contained within the 'gold standards' created was not available online.

The professional participants' declarations varied markedly, with some having minimal or only professional declarations to make (minimum 6, including job roles) and others having multiple financial declarations (maximum 20, e.g., sponsorship, consultancy, shares, private practice, patents, multiple professional roles). Within NHS England categories of declarations (financial, non-financial professional, non-financial personal, and indirect) some participants had multiple in one category and others were blank.

Phase 2:

Survey findings

In the second phase, 378 individuals participated and answered at least some of the questions. 266 (70%) were female and the vast majority lived in the UK (354, 93%). 141 (37%) described themselves as lay (citizens/patients), and the remainder as either training, trained or working in healthcare. The median age was 50-59 (range 18-80+). The majority of healthcare professionals participating were GPs (59, 25% of healthcare participants) followed by physicians and nurses. Of 230 healthcare professionals, 41% (95) filled in between 1-4 declaration of interest forms per year, with 35% (81) filling in none, and 23% (94) completing more than 5 (Appendix 5). A few participants did not complete all parts of the survey, meaning that some totals vary.

For peer review only

In the vignettes, when no interests were actively declared, 95% (335) of participants trusted the doctor either moderately, a lot, or completely and the same number felt they were acting in their best interests. If the doctor declared that they had been sponsored to travel to a conference by the company making a recommended joint replacement, the trust scored at moderately, a lot or completely fell to 79% (184) with 83% (286) judging as acting in their best interests. If the doctor declared heavier financial interests, including a patent and shares in the company, 54% (187) trusted the doctor moderately, a lot or completely, with a similar number (53%, 184) believing the doctor was acting in their best interests. When the doctor did not declare any interests, but was subsequently found by the participant to be acting as a consultant to the company, 22% (74) trusted the doctor moderately, a lot or completely, and 24% (80) felt they were acting in their best interests. There was a clear progression of decreased trust and decreased belief that the doctor was acting in their best interests with increasing financial interests. The least trust-generating scenario was where the conflict was not directly disclosed (Appendix 6, 7).

Survey respondents were then asked if they wished to continue to the task of searching for an individual's declarations of interest. 297 participants responded positively. 169 reported some data. 128 reported no findings. Each participants' findings were individually assessed to determine the proportion of declarations located within each type of category of interest, as described in the HPPs' 'gold standard' (Appendix 8). 61 found a source designed as a formal declaration of interest, for example, on guideline committee websites, which should have made it 'fit for purpose', containing at least some of the information from each of the four categories of NHS England guidance on declarations (financial professional and non profession, non-financial professional and non professional).

However, these formal, online declarations, despite being designed to be 'gold standard', were not all complete when compared to the standard we had generated with the HPP. 5 participants were able to replicate the 'gold standard' in their search. The top 5 highest cited links for the amount of accurate declarations were held in an organised register, such as NICE, parliamentary register, government website, or whopaysthisdoctor.org. When asked how long it should take to find a healthcare professional's declaration of interests, participants gave a range of 0-120 mins, mean 8.63 min.

Participants were asked how easy or difficult they found the search. Of 212 answering, 67.5% (143) said it was extremely or very difficult, and 36 (16.9%) somewhat or extremely easy. 76.8% (163) were not confident they had found all necessary information. 86% (184/213) said that finding declarations of interest were definitely or probably important.

Qualitative interview findings

At the end of the survey, all 50 lay people who consented were invited for an interview. All were individually contacted. 21 patients/citizens agreed to take part in this third phase and are referred to as they self-described. Thirteen were females and eight male; four described themselves as activists, and six as patients, with a variety of backgrounds including working in the pharmaceutical industry, research, or the public sector. The age range was 18-80, mean 62. Interviews lasted between 19-51 minutes and were transcribed verbatim for analysis. Participants could review their transcript. Nine requested them and three returned them with further comments; one corrected minor inaccuracies and the others added further comments.

Four themes were developed: *COIs in healthcare are difficult to define; Declarations of interest can be hard to find; COIs may present both challenges and benefits for patients; and COIs need to be carefully managed*.

For peer review only

Additional quotations are included in Appendix 9. Demographic information on participants is contained in Appendix Table 1.

COIs in healthcare can be difficult to define

Consistently, COIs were described as situations where care and treatment decisions benefitted the professional before the patient. However, although participants described a wide range and descriptions of COIs, they agreed that the concept and definition could be difficult to define. Some participants had acquired an understanding of COIs and declarations from different roles, including patient representative roles and experience of working in research or industry.

I know very little about it…. It makes me think of when drug companies go round, and they do a lunch for the doctors and they do a presentation about their product and the doctors prescribe their product. But I feel it's much wider and there's lot of other circumstances. I probably don't know much more other than a vague awareness that there are these other circumstances where there are potentially conflicts of interest. I don't really know much about them". (P1, patient)

Perceptions of a COI included a variety of financial COIs, particularly from industry. Examples of non-financial COIs given included gifts from pharmaceutical companies, HCPs holding powerful positions in decision-making bodies, involvement in research, considerations of reputation and career, or sponsorship from companies.

"… they might be offered honorarium or stipends, or a range of services, such as ghostwriters for medical journals, which will indirectly enhance or directly enhance their professional standing and from that flows their ability to garner research funding and have high profile research teams, so one can very directly benefit…even if the money isn't coming to you if it's coming to your research centre, it gives some people a lot of influence..." (P16, patient with long term condition)

Conflicts could also be caused by the way healthcare systems were organised and funded. Levers within the NHS could include GPs prescribing generically to save money, incentives to promote a product, or 'up-selling' on top of routine care. These could be regarded as a conflict, particularly when there was an uncertain justification.

"*do I need anti-glare on my glasses? I don't really know. But the lovely young person tells me that it's the best thing ever. So...the transaction becomes more complicated. Because there's a commercial element to the transaction that's being played out." (P19, lay, routine appointment for eyesight check at optician)*

These conflicts could be unforeseen, and related to influence and power. For instance, professionals' involvement in research was a potential conflict, as interest in certain outcomes could lead to biased treatment recommendations.

"*Well, it means that the prescriber or the provider of the service is making decisions that aren't just in the interest of the patient or the user. That they may give too much priority to their own interests. Those might not be financial. They might be their research project, or something that they're supporting. But it's not putting the patient first, second, third and fourth, as it were." (P4, patient)*

Participants expressed concerns that professionals may lack insight into their conflicts or their potential impact, due to unclear definitions, the lack of oversight of COIs or professionals' unconscious bias. Participants thought that peer review, and training on how to recognise COIs could potentially help. Respondents also argued that personal beliefs may result in unconscious bias; intellectual bias was also viewed as damaging. One participant felt that HCPs do not understand the bias in sciences generally and therefore do not recognise the role of reflexivity when evidence is scrutinised to inform treatment decisions.

"*So, I actually think healthcare professionals don't understand what conflict of interest means…They don't understand biases in science. They don't understand their need for not only reflection, but reflexivity. That they need to reflect on their own values and beliefs in what they're bringing to the evidence that they're presenting to patients." (P7, patient activist)*

Declarations of interest in healthcare can be hard to find

While participants were not asked directly about trust and transparency, patient activists and representatives repeatedly expressed concern about how the difficulty finding declarations could lead to a loss of trust in the medical profession.

"*I think that in that last scenario, the reason why my trust diminishes is because of lack of transparency. I think that is why would you not tell someone?...Why would you not tell someone that you have a financial interest in this? So I suppose it's the discovery - you feel like you've been duped." (P10, activist)*

For some, transparency was a tool to navigate the vague nature of COIs, enabling patients to make decisions and reinforce the trust in HCPs. Equally, participants highlighted their desire for and expectation of trust in the medical profession. Undisclosed COIs could lead to potential or actual loss of trust, particularly when conflicts were undisclosed.

"*I'd like it to be easier…. and I may not have found all of them." (P13, lay, patient representative roles)*

COIs present both challenges and benefits for patients

None of the participants questioned the value of research. However contradictory aspects of COIs were raised by participants in relation to the interplay between industry and the profession. Some participants thought that industry funding for education might be worth accepting for their potential benefits. A small number (who also represented participants who had worked in the pharmaceutical industry) highlighted the positive role of industry/the private sector in providing education and training to trainees and HCPs. One participant described this as justified:

"*yes, we need to have the declaration of a conflict of interest, but we actually have to allow a little bit of a conflict of interest for them to get to congresses and get educated…because if they don't, as I say, it's to our detriment ultimately, I believe…" (P17, activist, experience working in industry)*

Others recognised the potential for industry sponsored education to be a potential conflict (professionals obtaining free education that would otherwise have to be paid for) and biased in nature, leading to poorer quality healthcare.

"...One assumes that they were being promoted by the company at the time. The vaginal mesh incidence, again, one assumes across the gynaecological board, they were being hyped as the best thing. And that has ruined people's lives, in fact, it's killed some people." (P9, lay)

Complexity was described. COIs (such as sponsored education) could lead to improved care, but the presence of a conflict could lead to an assumption that decisions were not in the best interest of the patient - whether or not they were. Two participants described suffering harm because of COIs around the treatment they received. Others had heard about such experiences or felt that their health needs were not always the primary consideration of the HCP because of conflicts. They discussed how COIs can lead to corruption and poor patient care by referring to the difficulties in defining and acknowledging COIs and the role of the trust in patient-HCP relationship.

"*I like to start from the point that people are trustworthy and doing things from a good motive… There's a scenario where that doctor is somebody who genuinely wants to help patients, genuinely believes in the product, genuinely thinks that it's the right product for me, and happens to have received some payment for his role in developing it because his expertise has a value." (P1, patient)*

COIs need to be carefully managed

Participants overall described COIs as challenging to manage, citing a combination of difficult definitions, variable significance, and the different amounts of information patients were felt likely to want. The potential for information overload for patients, complicating decision-making, was discussed. Some patients described the practical difficulties of HCP making disclosures in a time-limited consultation, and the burden then put on patients to effectively consider and/or manage them.

"*I can also see that people, perhaps, in a consultation, are overwhelmed with information about their health… And it's so very difficult to know how relevant it is and whether it's really something that is swaying their judgement or not." (P6, citizen)*

Concerns were expressed about workload for HCPs if more regulations were introduced, with extra time needed for disclosure and explanation rather than for direct patient care.

"*And also, these professionals are very precious to you, they don't have much time, and you don't want to be talking about conflicts of interest when you actually want them to help you to do what it is that they're going to do." (P5, activist)*

Participants suggested ways of managing COIs, including better transparency. Several participants suggested that a mandatory register or regulation, like a Sunshine Act (6) might be a useful way of managing COIs. Respondents also wanted to see the same rules applied across the whole NHS as variation across the health boards was seen to lead to fragmentation and variation in care.

"*I think there should be… This Sunshine Act in America they have to declare… And I thought well that's open to interpretation but at least it would be something, so it would make it more ethical." (P12, lay)*

However, systems were needed to deal with COIs in ways that accounted for medical power. Complexity was again reflected. Participants wondered if simple disclosure would make a difference to patients. For example:

"*Most patients won't ever question this. They will never question their doctors." (P2, lay)*

A view held by several participants was that managing COIs should not rest with patients, but be a professional duty. The multiplicity of interactions meant that patients should be protected because a simple statement of interests could not suffice.

"*But does that capture the reality of the communications between doctor and patient, where so much is non-verbal, or implicit? …Relationships with employer, relationship with fellow professionals, relationship with marketing reps and others. The more you look at it, the harder it gets, I found." (P3, lay)*

Participants felt that independent advocacy could aid vulnerable people in consenting to treatment, if there were concerns around COIs. Second opinions and the presence of independent advocates were also seen as ways to mitigate bias. Independent parties would be able to raise issues of concern in relation to COIs:

" I think I would need an advocate with me to discuss my options. So, that's somebody who understands the medical side, but also somebody who is trained in advocacy and is a patient advocate…" (P21, lay)

However, again, complexity was acknowledged as independence was not always guaranteed.

"*I work in the area of health communication….the challenge I'm finding is that most of the patient advocates are sponsored by drug companies. And they've no regulation around that. There's no guidelines…it just seems a bit murky to me and not transparent." (P7, activist)*

Some participants highlighted where a declaration of a conflict may not necessarily reveal a negative bias. This could lead to patient concern about a conflict that was not in fact significant.

"*that doesn't mean somebody's not a good person and they're not doing the right thing…you know, he really believes in all the stuff…But he must be biased by that. Is that good bias? Is that bad bias? I don't know, you know?…" (P15_Carer)*

Participants suggested 'spot checks' (*P17*, *activist*) or oversight by an independent body to ensure that HCPs do not submit unsubstantiated information. This contrasted with discussions around trying to limit additional bureaucracy. Checking, peer review, and training were suggested on how to recognise COIs and potentially reduce unconscious bias. Other professions, particularly the public sector, law, Parliament and academia were compared, reflecting a shared view that professionals had duties to organise effective management.

"*I think the way it could be solved is that doctors with direct financial interests don't take on cases in that particular area. Obviously, that's quite serious, but I can't think of any other way that it could be totally solved. You know, essentially like the legal profession, people recuse themselves from cases. So that's just essentially the same principle". (P18, citizen)*

Discussion:

This is the first study we are aware of to test whether declarations of interest, as currently made by UK based, publicly facing doctors, could be located by lay people and professionals. In the second phase of the study, in vignettes describing an increase in financial declarations, participants' confidence in the doctor acting in their best interests, and their trust, fell. While 85% of participants thought that knowing about professional declarations was 'definitely' or 'probably important', despite generous marking, 27% (58) participants found a relevant link to 'fit for purpose' information and only 2% (5) found all components of the 'gold standard'.

All the professionals taking part in the study were part of an organisation with an official need to publicly declare interests. This study has demonstrated that, despite the efforts of the NHS to improve practice (e.g. NHS Trusts holding public registers of interest), transparency is not being effectively achieved because participants were unable to locate the registers with reasonable ease.

The third phase of this study invited patients and citizens, who had participated in the second phase, to interview. Declarations and conflicts presented complex challenges. Participants described ways to improve the system, but were also concerned for practicality, opportunity cost and bureaucracy. This was particularly in terms of where declarations should be made, when they were relevant, and how patients could feasibly use these. Multiple trade offs were described. For example, free but sponsored education and training potentially resulted in professionals having more up-to-date knowledge but conflicts of interest could be produced by these funders. Medical power had to be mitigated. Professionals may lack insight into their own bias, and independent oversight would be necessary to mitigate and check compliance. Some expressed concern of the additional burden that could be placed on doctors, and also patients to locate and interpret declarations. Free text responses in the searches confirmed this, for example "Despite searching I found no good way to find any" "I could not easily find anything apart from some stuff on Wikipedia" "Sorry, I struggle to find any. Giving up." "I am finding this task really difficult. There is too much information to work through. Patients should not have to do this research."

Strengths of this study include the collaboration of a patient panel to develop the survey, suggest professional participants, and disseminate invitations to participate. Additionally, it was highly pragmatic, mimicking the steps that a citizen would take to investigate a conflict. This is the first study we are aware of which tested current UK declaration processes. It is also the first we are aware of in the UK which interviewed laypeople to elucidate understanding and concerns about conflicts of interest in healthcare professionals, and what improvements could consist of. There were several limitations. The HPP were by definition in the public eye, and willing to take part, and may have been more likely to use a high profile register eg on government websites. However this would have resulted in an over estimation of being able to locate conflicts, meaning that the results would be artificially high. Only one conflict in each category (e.g. professional financial, indirect) had to be found to be scored correct, leading to an overestimation of the effectiveness of current practice. Declarations were scored equally, however, the relative importance of each declaration is not, in reality, equal: some may have been unimportant and very unlikely to cause important conflict; others, the opposite. Further, the participants and professionals who took part in our study are likely atypical, with engagement with these issues prior to the request for participation. It is not expected that many citizens would normally spontaneously search for healthcare professionals' declarations or conflicts. Our participants are likely to have engaged with some or many of the issues related to conflicts of interest, given that our patient group assisted in sending the questionnaire widely to engaged patient groups. Multiple entries to the questionnaire by one person under different email addresses would have been possible but we consider this overall unlikely to have had a large impact on results. Nevertheless, even in a group of activated professionals and citizens, finding a complete declaration of interest was extremely difficult, and a partial finding of declarations was possible only a

For peer review only

minority of the time. Participants agreed, reporting a low level of confidence that their results were complete.

While there are no directly comparable studies, other US work (31) supports the finding that patients, including potential research participants, wish transparency and to know the researchers' conflicts of interest. The impact of disclosure of a doctor's conflicts of interest to patients via a mailed letter has been investigated in the US (32). Patients subsequently described an increased level of confidence in their ability to judge potential impacts of conflicts on their healthcare. Overall there was no change in described levels of trust in doctors. However, in patients who three months later recalled receiving the disclosure notice, around a fifth described increased trust in the physician. Given what is known about the potential for harm from financial conflicts of interest, it is questionable whether this is a good outcome, as people may trust advice even if it is at risk of bias. Sah et al. (33) investigated trust in scenarios, mainly concerning financial advice. People were asked about their levels of trust in financial advisors where the advisor had concealed or unconcealed conflicts of interest, and where the interests of client and advisor were aligned or not. Trust was found to be reduced when a conflict of interest was known, even when the interest of client and advisor was congruent and the advice was high quality. The final scenario concerned a medical vignette. This found that a disclosure of a conflict resulted in increased trust in participants. However the vignette featured a doctor's recommendation not to do a test, which would otherwise have attracted a fee. This so-called 'altruistic signal' is theorised to offset the 'disclosure penalty' which can otherwise reduce trust. Finally, a field experiment in the US (34) randomised patients to receive a hospital appointment letter containing, or not, the doctor's conflict of interest statement. Patients receiving the disclosure reported more knowledge of these conflicts, with no change in trust or appointment attendances. These studies were set in the US, where there are major cultural differences concerning health service delivery, and are unlikely to be directly applicable to dissimilar countries such as the UK.

This research has found that lay people hold mixed and often nuanced views over conflicts of interest. Further, the practical aspects of declarations, including organisation, workload for both patients and doctors, and interpretation, was realised to present difficulties. Given the strong decline in trust in the vignettes with increasing conflicts of interest, it is uncertain whether an 'altruistic signal' would compensate for a 'disclosure penalty'. Further, the 'medical power' which laypeople alluded to must still be negotiated where conflicts are found, an aspect not investigated in these other studies, although Pearson et al. (35) found that half the patients who remembered seeing a disclosure did not feel they knew enough to judge the potential impact of it.

This leads to basic questions about the use and purpose of declarations of interest in the UK. Should they be for transparency alone? Should declarations be intended as more than an 'information dump' but made in ways which enable judgements - and effective management?

Unintended consequences of transparency are possible, for example, 'moral licence', where disclosure is assumed to negate potential bias. There is evidence that doctors believe that other doctors become biased when exposed to small gifts, while they themselves do not (36). Further, the survey and interviews confirmed that a lack of transparency in professionals leads to less trust for patients. If more transparency was created, and trust in conflicted doctors increased, it is uncertain whether this would be justified, given the evidence that financial conflicts of interest are associated with bias, and more expensive poorer quality healthcare (37, 38). Indeed, previous research has found that declarations of interest, for example, those recommended by the International Committee of Medical Journal Editors, are of poor quality and make it difficult for the reader to assess bias (39).

For peer review only

Transparency may therefore not be a benign act. Given that most UK Hospital Trusts do not state the action planned to mitigate a publicly declared conflict, it may not be clear where the work of finding, interpreting and managing conflicts of interests rests (21). Many respondents were concerned as to what to do with the conflicts located. Some felt they could trust the doctor regardless of a conflict, as disclosure mitigated bias; others felt it difficult to know whether they could trust the doctor's judgement despite a declared conflict, and would require advocacy to assist. While patient organisations were suggested as potential advocates, there is also evidence that some are themselves conflicted (40).

The General Medical Council (UK) recommends doctors "avoid conflicts of interest wherever possible" and "declare any conflict to anyone affected, formally and as early as possible, in line with the policies of your employer or the organisation contracting your services". They also recognise "Conflicts of interest are not always avoidable… follow(ing) established procedures for declaring and managing a conflict" (13). The risk is that disclosure is used to include rather than exclude individuals from relevant decision making, in the belief that a disclosure constitutes management. While disclosure is necessary for management, it does not substitute for it. This is particularly important when considering the evidence on unconscious bias from professionals, and the risk of trusting conflicted, but declaring, professionals where patients have limited power to know about or mitigate the potential impact of a conflict.

Further research should elucidate what the purpose(s) of declarations of interest should be for different groups of people and find ways to meet these needs. For example, if it is for simple transparency, declarations need to be easy to find and understand. If they are to manage conflicts, a clear decision on boundaries may be helpful. However, changes should be tested not just with patients, but professionals, as issues of workload and opportunity cost were reflected in interviews. Further, research should help to understand the best ways of making declarations which allows the reader to make an evidence-based interpretation of their potential impact. Our study relied on interested parties to participate, and research on panels recruited from the wider population would be helpful. Professional views should also be sought to understand what the facilitators and barriers are to making declarations in order to organise the best way to declare and manage them. Finally, the value of making declarations has yet to be established, beyond transparency. A more reliable way to manage them may be via better processes of disallowing certain conflicts from defined roles, rather than trying to manage them using haphazard declarations of interest. Further qualitative work may help to understand how conflicts are managed in practice.

Conclusion

Declarations of interest by professionals are agreed to be important, but are unfit for purpose in their current form. The survey found that patients describe trusting professionals with no conflicts the most, and professionals with undisclosed conflicts the least. The practical task of finding declarations of interest for well-known doctors in the public domain was difficult. Even when disclosure statements were found, most were incomplete when compared to the 'gold standard', which were rarely located. Interviews with lay people found nuanced views about disclosure and management of conflicts. They were described as important, but difficult to find and use, and although some potential conflicts could be justified, they needed to be managed. Currently practitioners making declarations cannot be assured that this information can be readily found, and cannot assume that this information can be used in decision making by laypeople. Other research finds that transparency may result in unintended consequences, including placing trust in professionals who may take 'moral licence' from an open declaration, while increasing workload for patients and professionals. Together, these may cause unintended harms. Patients may not feel able to use information about conflicts of interest to their advantage. This means that declaring potential conflicts should be refined and greater professional emphasis of avoiding, identifying and managing serious conflicts of interest with clear, public definitions on who requires exclusion from what types of decision making, be considered. It is questioned whether the purpose of declarations should rest on providing mere transparency, but be used to exclude, rather than include, conflicted professionals in relevant decision making.

For peer review only

Acknowledgements

We thank the health professionals who took part in this study for their time and valuable assistance and support; our patient panel, Lynne Craven, Brian Finney, Fiona Raje, and Kath Samson for their help with study design, invitations and recruitment, and Professor Carl Heneghan who discussed and advised on the study at conception. We also thank the peer reviewers for their constructive and helpful comments.

Copyright

The Corresponding Author has the right to grant on behalf of all authors and does grant on behalf of all authors, an exclusive licence (or non exclusive for government employees) on a worldwide basis to the BMJ Publishing Group Ltd to permit this article (if accepted) to be published in BMJ editions and any other BMJPGL products and sublicences such use and exploit all subsidiary rights, as set out in our licence.

Transparency declaration

MM affirms that the manuscript is an honest, accurate, and transparent account of the study being reported; that no important aspects of the study have been omitted. We had originally planned to run the online questionnaire for 4 weeks but extended to 8 weeks as we were still receiving responses.

Protocol

This is enclosed as per the registration document. It is marked Appendix 10.

Checklist

We enclose CROSS checklist (for survey designs) and SRQR (for qualitative research) as recommended by EQUATOR.

Contributorship

Study conceived by MM with design by MM and FS. Questionnaire design by MM, RM, GO and AMB with assistance and direction from the patient panel. Analysis of questionnaire and survey data by RM, MM and FS. Qualitative interviews by KM and MM. Analysis of interviews by KM, AMB, GO, MM. Draft of paper by MM with editing and contributions from all.

Declaration of interest: MM has been paid for writing/broadcasting in relation to issues connected with campaigning around conflicts of interest. All authors have completed the Unified Competing Interest form (available on request from the corresponding author) and have no other declarations to make regarding support from any organisation for the submitted work; financial relationships with any organisations that might have an interest in the submitted work in the previous three years.

Funding: University of St Andrews Research Fund. Grant award number: NA.

Data sharing

No further data is available. This is because our HPP gave consent on the basis of confidentiality of their identity, which would not be possible if further data was made public.

References

1. Taichman DB, Backus J, Baethge C, et al. A Disclosure Form for Work Submitted to Medical Journals: A Proposal From the International Committee of Medical Journal Editors. JAMA. 2020;323(11):1050–1051. doi:10.1001/jama.2019.22274

2. NHS England publish new guidelines on tackling conflicts of interest. 9/2/17 https://www.england.nhs.uk/2017/02/coi-guidelines/

3. Spithoff S, Leece P, Sullivan F, Persaud N, Belesiotis P, Steiner L. Drivers of the opioid crisis: An appraisal of financial conflicts of interest in clinical practice guideline panels at the peak of opioid prescribing. PLoS One. 2020;15(1):e0227045. Published 2020 Jan 24. doi:10.1371/journal.pone.0227045

4. Wang AT, McCoy CP, Murad MH, Montori VM. Association between industry affiliation and position on cardiovascular risk with rosiglitazone: cross sectional systematic review. BMJ. 2010;340: c1344–c1344. 10.1136/bmj.c1344

5. Fickweiler F, Fickweiler W, Urbach E. Interactions between physicians and the pharmaceutical industry generally and sales representatives specifically and their association with physicians' attitudes and prescribing habits: a systematic review. BMJ Open. 2017;7(9):e016408. Published 2017 Sep 27. doi:10.1136/bmjopen-2017-016408

6. UK Research and Innovation. How we're governed: Conflicts of interests. 12/5/22 https://www.ukri.org/about-us/how-we-are-governed/conflicts-of-interests/

7. First do no harm: The Report of the Independent Medicines and Medical Devices Review. 8/7/20 https://www.immdsreview.org.uk/Report.html

8. The influence of the Pharmaceutical Industry. 2014/5 session Health Select Committee UK Parliament https://publications.parliament.uk/pa/cm200405/cmselect/cmhealth/42/42.pdf

9. The sun shines on Europe. Transparency of financial relationships in the healthcare sector. 2017 Health Action International https://haiweb.org/wp-content/uploads/2017/03/Sunshine-Act.pdf

10. Fabbri, A., Santos, A., Mezinska, S., Mulinari, S., Mintzes, B. (2018). 'Sunshine Policies and Murky Shadows in Europe: Disclosure of Pharmaceutical Industry Payments to Health Professionals in Nine European Countries', International Journal of Health Policy and Management, 7(6), pp. 504-509. doi: 10.15171/ijhpm.2018.20

11. Kanter GP, Carpenter D, Lehmann L, et al Effect of the public disclosure of industry payments information on patients: results from a population-based natural experiment BMJ Open 2019;9:e024020. doi: 10.1136/bmjopen-2018-024020

12. Policy on declaring and managing interests for NICE advisory committees. National Institute for Health and Care Excellence. September 2022 https://www.nice.org.uk/Media/Default/About/Who-we-are/Policies-and-procedures/declaration-of-interests-policy.pdf

13. Financial and commercial arrangements and conflicts of interest. General Medical Council. Accessed 10/2/23 https://www.gmc-uk.org/ethical-guidance/ethical-guidance-for-doctors/financial-and-commercial-arrangements-and-conflicts-of-interest/financial-and-commercial-arrangements-and-conflicts-of-interest

14. Lo B, Field MJ. Conflict of interest in medical research, education and practice. Institute of Medicine of the National Academies, Committee on Conflict of Interest in Medical Research, Education and Practice. IOM (Institute of Medicine) 2009. https://nap.nationalacademies.org/read/12598/chapter/1#ii

15. Wilson M. (2014). The Sunshine Act: commercial conflicts of interest and the limits of transparency. Open medicine : a peer-reviewed, independent, open-access journal, 8(1), e10–e13.

16. The PLoS Medicine Editors (2012) Does Conflict of Interest Disclosure Worsen Bias? PLoS Med 9(4): e1001210. https://doi.org/10.1371/journal.pmed.1001210

For peer review only

17. Susannah L. Rose, Sunita Sah, Raed Dweik, Cory Schmidt, MaryBeth Mercer, Ariane Mitchum, Michael Kattan, Matthew Karafa, Christopher Robertson, Patient responses to physician disclosures of industry con-flicts of interest: A randomized field experiment, Organizational Behavior and Human Decision Processes, 2019, ISSN 0749-5978 https://doi.org/10.1016/j.obhdp.2019.03.005.

18. Pearson SD, Kleinman K, Rusinak D, Levinson W. A trial of disclosing physicians' financial incentives to patients. Arch Intern Med. 2006 Mar 27;166(6):623-8. doi: 10.1001/archinte.166.6.623. PMID: 16567600

19. Hampson et al. Patients' views on financial conflicts of interest. N Engl J Med 2006; 355:2330-2337 DOI: 10.1056/NEJMsa064160

20. Kmietowicz Zosia. Disclosure UK website gives "illusion of transparency," says Goldacre BMJ 2016; 354 :i3760

21. McCartney M, Bergeron Hartman R, Feldman H, et alHow are declarations of interest working? A cross-sectional study in declarations of interest in healthcare practice in Scotland and England in 2020/2021BMJ Open 2022;12:e065365. doi: 10.1136/bmjopen-2022-065365

22. Macdonald Helen, McCartney Margaret, Heneghan Carl, Godlee Fiona. Doctors' conflicts of interest BMJ 2020; 370 :m3247

23. Fadlallah R et al. Knowledge, Beliefs and Attitudes of Patients and the General Public towards the inter-actions of Physicians and the Pharmaceutical and Device Industry: a systematic review. PLOS 24/8/1

24. Glogowska, M. (2011), Paradigms, pragmatism and possibilities: mixed-methods research in speech and language therapy. International Journal of Language & Communication Disorders, 46: 251-260. https://doi.org/10.3109/13682822.2010.507614

25. Bishop FL. Using mixed methods research designs in health psychology: an illustrated discussion from a pragmatist perspective. Br J Health Psychol. 2015 Feb;20(1):5-20. doi: 10.1111/bjhp.12122. Epub 2014 Nov 18. PMID: 25405549.

26. Barriball, LK. and While, A. (1994), Collecting data using a semi-structured interview: a discussion paper. Journal of Advanced Nursing, 19: 328-335. https://doi.org/10.1111/j.1365-2648.1994.tb01088.x

27. Glogowska, M. (2011), Paradigms, pragmatism and possibilities: mixed-methods research in speech and language therapy. International Journal of Language & Communication Disorders, 46: 251-260. https://doi.org/10.3109/13682822.2010.507614

28. Bishop FL. Using mixed methods research designs in health psychology: an illustrated discussion from a pragmatist perspective. Br J Health Psychol. 2015 Feb;20(1):5-20. doi: 10.1111/bjhp.12122. Epub 2014 Nov 18. PMID: 25405549

29. Barriball, LK. and While, A. (1994), Collecting data using a semi-structured interview: a discussion paper. Journal of Advanced Nursing, 19: 328-335. https://doi.org/10.1111/j.1365-2648.1994.tb01088.x

30. Braun V, Victoria Clarke V. Using thematic analysis in psychology, Qualitative Research in Psychology, 3:2, 77-101, (2006) DOI: 10.1191/1478088706qp063oa

31. Kim SY, Millard RW, Nisbet P, Cox C, Caine ED. Potential research participants' views regarding researcher and institutional financial conflicts of interest. J Med Ethics. 2004 Feb;30(1):73-9. doi: 10.1136/jme.2002.001461. PMID: 14872080; PMCID: PMC1757115.

32. Pearson SD, Kleinman K, Rusinak D, Levinson W. A trial of disclosing physicians' financial incentives to patients. Arch Intern Med. 2006 Mar 27;166(6):623-8. doi: 10.1001/archinte.166.6.623. PMID: 16567600

For peer review only

33. Sah S, Loewenstein G, Cain D. Insinuation Anxiety: Concern That Advice Rejection Will Signal Distrust After Conflict of Interest Disclosures. Pers Soc Psychol Bull. 2019 Jul;45(7):1099-1112. doi: 10.1177/0146167218805991. Epub 2018 Nov 17. PMID: 30451077

34. Rose SL, Sah S, Dweik R, Schmidt C, Mercer M, Mitchum A, Kattan M, Karafa M, Robertson C. Patient responses to physician disclosures of industry conflicts of interest: A randomized field experiment. Organizational Behavior and Human Decision Processes,
Volume 166, 2021, Pages 27-38, ISSN 0749-5978, https://doi.org/10.1016/j.obhdp.2019.03.005.

35. Pearson SD, Kleinman K, Rusinak D, Levinson W. A trial of disclosing physicians' financial incentives to patients. Arch Intern Med. 2006 Mar 27;166(6):623-8. doi: 10.1001/archinte.166.6.623. PMID: 16567600

36. Steinman MA, Shlipak MG, McPhee SJ. Of principles and pens: attitudes and practices of medicine housestaff toward pharmaceutical industry promotions. Am J Med. 2001 May;110(7):551-7. doi: 10.1016/s0002-9343(01)00660-x. PMID: 11347622.

37. Nejstgaard CH, Bero L, Hróbjartsson A, Jørgensen AW, Jørgensen KJ, Le M, Lundh A. Association between conflicts of interest and favourable recommendations in clinical guidelines, advisory committee reports, opinion pieces, and narrative reviews: systematic review. BMJ. 2020 Dec 9;371:m4234. doi: 10.1136/bmj.m4234. PMID: 33298430; PMCID: PMC8030127.

38. Spurling GK, Mansfield PR, Montgomery BD, Lexchin J, Doust J, Othman N, et al. Information from pharmaceutical companies and the quality, quantity, and cost of physicians' prescribing: a systematic review. PLoS medicine. 2010;7(10):e1000352. pmid:20976098; PubMed Central PMCID: PMC2957394.

39. Grundy Q, Dunn A G, Bero L. Improving researchers' conflict of interest declarations BMJ 2020; 368 :m422 doi:10.1136/bmj.m422

40. Mandeville K L, Barker R, Packham A, Sowerby C, Yarrow K, Patrick H et al. Financial interests of patient organisations contributing to technology assessment at England's National Institute for Health and Care Excellence: policy review BMJ 2019; 364 :k5300 doi:10.1136/bmj.k5300

For peer review only

Appendix Table 1

Description of the interviewees by sex, age and self defined role

	Males	Females
Age group		
30-39	1	0
40-49	0	1
50-59	2	4
60-69	2	8
70-79	1	0
80+	2	0
Total	8	13
Status		
Citizen	1	3
Patient	2	2
Long term condition	0	2
Patient activist	2	2
Carer	0	1
Not specified	3	3
Total	8	13

Appendix 1:

Method of searching for declarations/conflicts of interest

General Google search for job title, roles, affiliations - first 10 pages “Firstname Lastname” + “NHS” + “private” + “clinic” + “press release”

“Firstname lastname” + “Conflicts of interest” + “declared interests”

Disclosure UK (APBI)

NHS Trust site / GP surgery site / any private clinics - search for Register of Interests, and search within, if not publicly available need to write to NHS Trust using FOI if necessary

NICE/SIGN search for 1) Firstname Lastname 2) if present, search for DOI

PubMed Firstname Lastname - search for declaration of interest

Linkedin

Companies House - UK registration of all limited companies

Electoral Commission - personal or company donations

Patents (Google Patents - patents.google.com)

LexisNexis (any mentions of companies / sponsors)

TV / Radio (transcripts accessible via Box of Broadcasts)

PubMed: Journal articles in last 5 years, conflicts of interest declarations

ClinicalTrials

OpenPayments.gov

Youtube lectures for any declarations of interest made

Appendix 2

Methods of recruiting lay people, patients, citizens and professionals to participation

1) Citizens - invited via social media and citizen groups including Sense about Science network
2) Patient activists - recruited via National Voices, Sling the Mesh, Patients Association UK
3) Healthcare students - via Medical Schools Advisory Council, nursing, midwifery, and paramedical school associations via social media, and via ELAG (Educational Leads Advisory Group) to contact medical students, medical school newsletters.
4) Healthcare professionals working independently eg consultants, pharmacists, independent nurse consultants/prescribers — via social media, medical press, medical school newsletters
5) Early career researchers - training forums, Colleges via social media.

Appendix 3

Vignettes used in online questionnaire

A

You have developed a problem with your hip. You go to see a doctor, who you have known for several years and they have always given good care. They are qualified to do their job.

The doctor recommends an operation in the NHS to replace your hip.

This is a type of hip replacement called Mars. You are told that this is the standard type of hip replacement used for people like you and usually has very good results.

The doctor tells you that they do not have any shares or patents in Mars, nor do they get paid extra to fit this type of hip replacement.

B

You have developed a problem with your knee. You go to see a doctor, who you have known for several years and they have always given good care. They are qualified to do their job.

The doctor recommends an operation in the NHS to replace your knee.

This is a type of knee replacement called Jupiter. You are told that this is the standard type of knee replacement that is used for people like you and usually has very good results.

The doctor tells you that they do not have any shares or patents in Jupiter. However the doctor says that they have been to a conference about this type of knee replacement. The company paid for them to travel to a conference in Europe and for a course about it, which would normally have cost a few thousand pounds.

C

You have developed a different problem with your other hip. You go to see a doctor, who you have known or several years and they have always given good care. They are qualified to do their job.

The doctor recommends an operation in the NHS to replace your hip with a type of hip replacement called Venus.

You are told that this is the standard type of hip replacement for people like you and usually has very good results.

The doctor tells you that they are part of the team that developed Venus hip replacements. The doctor works as a consultant for the company that makes them and travels to conferences to explain the hip replacement to other doctors. The doctor is a part-owner of the patent for Venus hip replacement and has shares in the company making them.

D

You have developed a different problem with your other knee. You go to see a doctor, who you have known or several years and they have always given good care. They are qualified to do their job.

The doctor recommends an operation in the NHS to replace your knee.

You are recommended a type of knee replacement called Saturn. You are told that this is the standard type of knee replacement used for people like you and usually has very good results.

You do a search online. You find that this doctor owns a patent and shares in Saturn. Last year the doctor received £50,000 for working as a consultant for the company.

Appendix 4

Criteria included in 'gold standard'

As many participants listed potential conflicts as items which were out of scope of ICMJE criteria/ NHSE criteria some were dismissed; for example:

Director of company dissolved >5 years ago
Single talk given to public >3 years ago
Public service job > 5 years ago
Honorary Fellow/Member of medical organisation
Examiner for a Royal College exam (reasonably supposed to be part of a job already declared)

Appendix 5

Estimation of how many forms filled in per year to declare interests by healthcare professionals

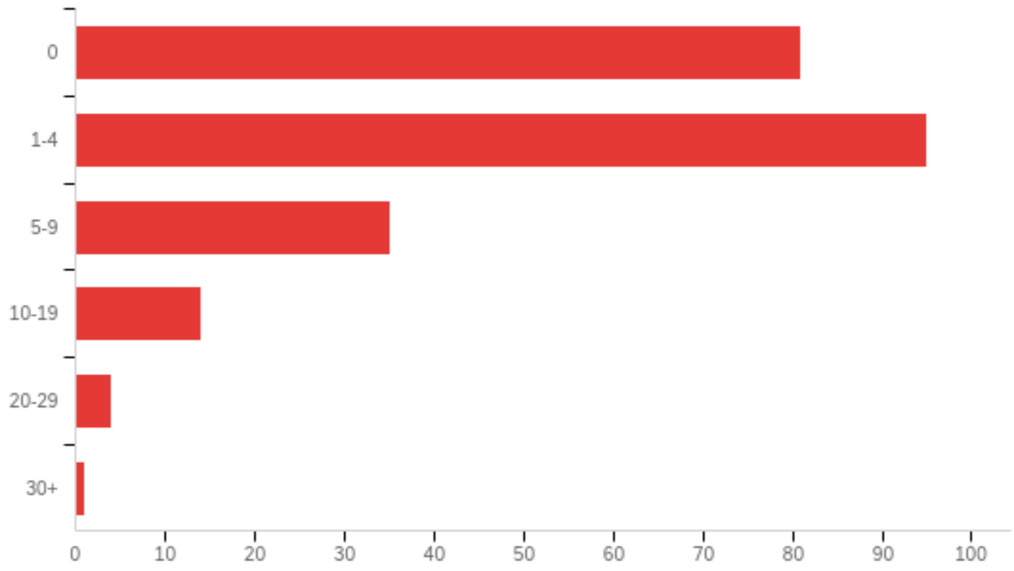

Appendix 6

Relationship of Trust to increasing conflicted scenarios

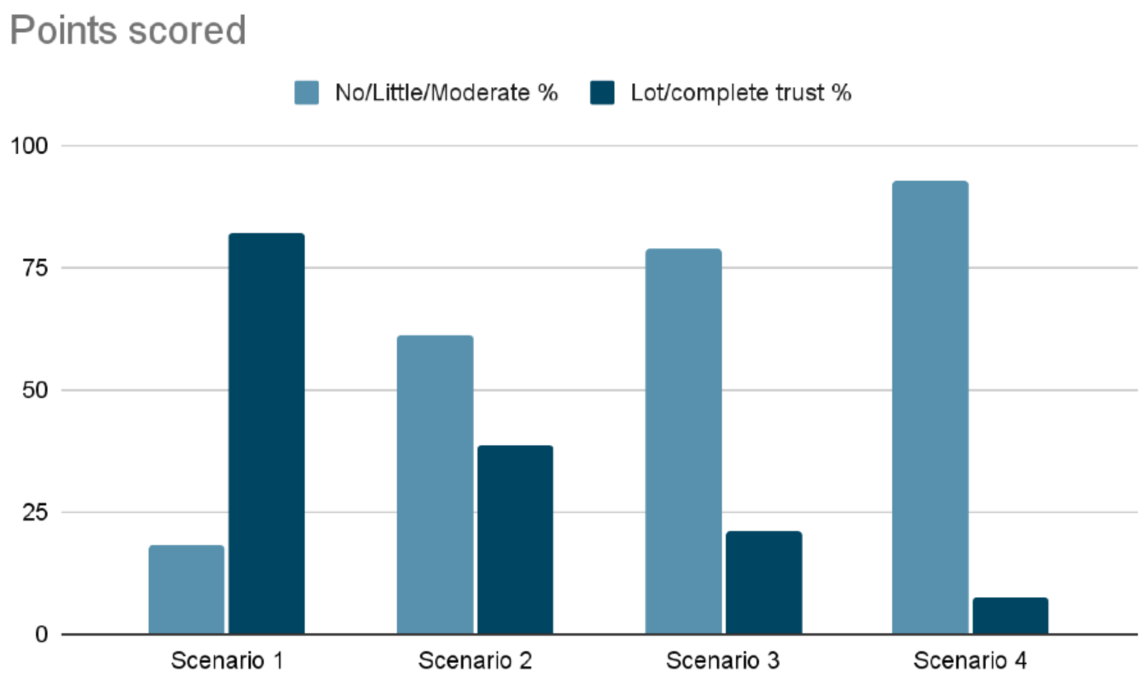

A	B	C	D	E
	No/Little/Mode	Lot/Complete trust		
Scenario 1	18.1%(64)	81.9% (289)		
Scenario 2	61.2% (211)	38.8%(134)		
Scenario 3	79% (270)	21.1% (72)		
Scenario 4	92.7% (315)	7.4% (25)		

Appendix 7

Relationship of belief doctor acting in patients best interest to increasing conflicted scenarios

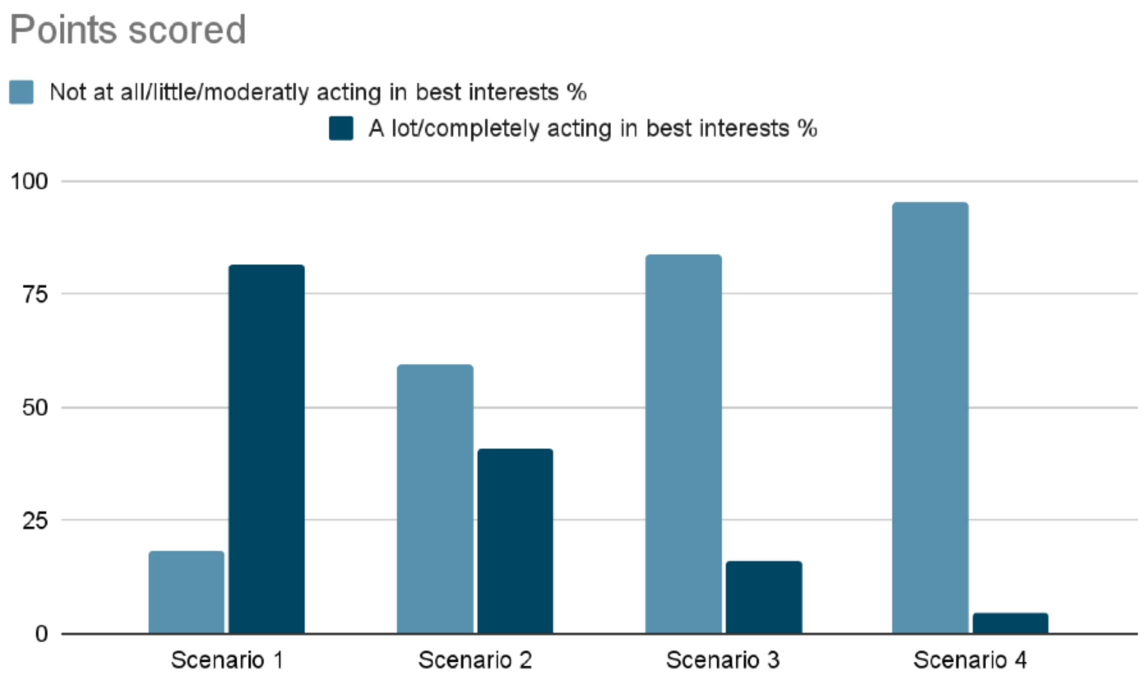

Scenario 1	18.5(65)	81.6 (287)
Scenario 2	59.3(204)	40.7 (140)
Scenario 3	83.7(288)	16.3(56)
Scenario 4	4.7 (16)	95.3 (324)

Appendix 8

Results - participants locating fit for purpose and gold standard declarations

Key:
'Links located" - total number of links reported by research participants
"Number fit for purpose" - weblinks containing a formal declaration of interests
"Participants reporting any results" - any information obtained was included
"Participants reporting a fit for purpose link" - number of participants finding a 'fit for purpose' link
"Significant entries%" - all significant entries including financial entries
Responses finding all categories - at least one entry from each category

Professiona	Links located	Number fit for purpose	Participants reporting any results	Participants finding a fit for purpose link	Average% significant interests found	Total responses containing each category of declaration
A	11	3/11	14/24	2	64%	8/14
B	14	3/14	12/24	6	57%	8/12
C	5	1/5	10/22	1	30%	3/10
D	6	5/6	10/21	5	90%	9/10
E	10	1/10	9/23	3	67%	6/9
F	9	6/9	14/24	8	79%	11/14
G	19	2/19	17/22	3	18%	3/17
H	17	3/17	15/22	2	20%	3/15
I	3	2/3	11/23	4	55%	6/11
J	8	2/8	16/23	10	75%	12/16
K	12	1/12	10/22	2	70%	7/10
L	12	4/12	16/25	11	100%	16/16
M	10	2/10	13/22	2	15%	2/13

Appendix 9

Additional qualitative data from interviews arranged by theme

Theme 1: COIs in healthcare can be difficult to define

"It means that either directly or indirectly, a health care professional stands to benefit from a decision that is made about the appropriate treatment for a patient". (P16_F60-69_LTC)
"It means to me, that a professional may have a financial interest in a firm which is manufacturin drugs or medical devices and may or may not declare that interest to the people that they are ca for or prescribing for". (P21_F60-69_Lay)
"It could be having a bit of a conference with a dinner or a few drinks or giving a few products fr They think it's their right" (P14_F50-19_Lay)
"you can have a conflict between the need for public health …. and the needs to provide service the person in front of you because it's an opportunity cost. … it's balancing out …. the exigent circumstances of the person in front of you or the group you're talking about it one time." (P16_F60-69_LTC)
**"*It could be not direct to them. It might be a contribution towards research to which they have ar interest. So, they think it's acceptable because it's going towards research, but it's clearly swayi the opinion and the reason behind things".* (*P14_F50-19_Lay*)**
"I think people struggle with understanding what a conflict of interest is because you could hav interest in something and not have any financial gain from it and not declare that interest …. I th on the whole, a lot of people don't declare interests because they don't really understand what t interests are and how they conflict". (P10_M60-69_Activist)
"Well, at the extreme end, it means doctors being involved with the direct purchase of equipme drugs that they have an interest in that company, so they therefore are likely to get greater divid back. Or if they're actually part owners of companies, … they're getting funds directly from company either through profits or when the company is sold. So that's a very strong confli interest". (P17_M60-69_Activist)
"one of the areas I thought of was where your beliefs, your beliefs will influence what you do. An think that that we don't look at that an awful lot. … So I'd be very, very careful that my unconscio bias doesn't conflict with professionalism, and I think that that's a really difficult area because y talking about thoughts, you know, rather than financial interests and sometimes you know that c be more insidious than, you know, money". (P10_M60-69_Activist)
"So, I actually think healthcare professionals don't understand what conflict of interest means. I think it's that fundamental. They don't understand biases in science. They don't understand thei need for not only reflection, but reflexivity. That they need to reflect on their own values and beli in what they're bringing to the evidence that they're presenting to patients". (P7_F50-59_Activist

“It's trust. This is what I said about bribery and all that type of thing. It's very sad because if you' the health profession, I always feel that these people should be within the heart, caring. It's all ab being a caring person.” (P14_F5-59_Lay)
“Personally, unfortunately, I've lost all trust of all items due to, I feel, a conflict of interest, happened to me. I always research everything. In fact, I refuse it now, anything. To a point you total trust with everything, and it can ruin people's trust in things, in medication, so it's (P14_F5-59_Lay)
“*I know very little about it…. It makes me think of when drug companies go round, and they do a lunc the doctors and they do a presentation about their product and the doctors prescribe their product. But it's much wider and there's lot of other circumstances…I don’t really know much about them”. (P1, lay)*
“*It's almost like a bribe, possibly, from the pharmaceutical… It could be a soft one, where maybe the me doesn’t recognise that it's happening, or it could be something that’s really quite obvious but they thin acceptable because there's no law there.” (P14, lay)*
“*...it’s to do with the power imbalance. You have gone to this professional for help because you don’t much about what is happening to you. So, it’s about trust… you trust them to do the right thing for because you don’t know how to do it yourself.” (P5, activist)*

Theme 2: COIs in healthcare can be hard to find

“...if a new product is brought to the market, then, understandably, if it’s going to be used it nee be promoted, so that’s understandable. But I don’t think it should be too difficult to have a syste transparency around that.” (P1_F40-49_Lay)
“I think that the openness and transparency should apply equally to both financial and non-finar and I don’t think… I suppose, financial, you assume is worse, if you like, or more likely to be n my best interest, but I think, actually, both equally need to be clear as to what’s happen (P5_F60-69_Activist)
“ … so in terms of conflict of interest, I don't think it's necessary for the patient to do a huge amo more. I think the NHS is aware of the issue and they've produced reports and whatever on the subject in past years, I believe. For me, the thing that's needed, and you know, I'm not sure how much this is in place, is the transparency and whistleblowing and ability to contest after the eve …” (P13_M70-70_Lay)

Theme 3: COIs present both challenges and benefits for patients

" … they got their paid research grants, and they were thinking well we could do this, or we co that, but now, I look back, I think that wasn't really in the patient's interests. Because the patie bamboozled with choices that she knew nothing about. … And you were still trusting implicit they had your best interests at heart when I think they were looking at interesting t opportunities for medical students. … I used to have a career and a nice home. I've lost everyth this undisclosed bias in favour of polypropylene implants." (P12_F60-69_Lay)
"I think when there are conflicts of interest, there's potential for corruption. Potential for corrupt always a problem... It depends on the rules that you have in place that stop corruption. … For m depends on what becomes from the norm and acceptable. … And in some of these conflicts of interest issues patients are moved into a commercial transaction where the distinction between diagnosis and treatment and the offer is being blurred. And you're then into being sold a produc which you might not completely need." (P19_F60-69_Lay)
"i's that complexity because it's about, you know, not assuming that the conflict of interest is a thing for everybody as well. Sometimes. Well, actually that might be good thing, cause I believe and I want somebody who also believes that." (P15_F50-59_Carer)
"*I don't have any huge problem. And I think my general view of health is that the right provider is som more important than keeping it in house, and you know the NHS tends to keep things in house rathe use it outside, but that's nothing, that profit in itself is not a not a dirty word…" (P13, lay)*
"*Because it can affect outcomes for people's care. There's been incidences of, for example, poor d being employed, hip replacement joints, I think there was a bad set of them at one point. One assum they were being promoted by the company at the time. The vaginal mesh incidence, again, one as across the gynaecological board, they were being hyped as the best thing. And that has ruined people' in fact, it's killed some people." (P9, lay)*

Theme 4: COIs need to be carefully managed

"So if I was looking for a private treatment for something that I might well go and look, you know there wasn't an everyday, not like a dentist or something like that. But I might then go and look a person, that individual and sort of look at at what they do and why they do it. And you can often that, you know, things that come up on Facebook and places. There are clear links between." (P15_F50-59_Carer)
"I think it's much more complex issue than that as we're dealing with human beings and it's real hard to unpick. And I think you have to go, I guess what you have to do is you have to go with th worst case scenario that somebody who's got… You know, some kind of major conflict of intere abuse that and therefore you have to put such systems and structures in place that avoid that w case scenario." (P15_F50-59_Carer)

"I don't think I would have known that you would find that…and so I suspect that if I don't know have got a background in the pharmaceutical industry, and medical friends and so on, I suspe the layperson would have very little idea that that was there to be found. So, I'm not sur expecting people to go and search online would be the best way to manage it. I think, probably, have to be even more upfront and declare it at the consultation." (P6_F60-69_Lay)

"Actually, I'm not even given the name of consultants a lot of the time if I go to hospital, but seeing a consultant, all their interests should be there. I shouldn't have to go looking for it. It s be very easy to find" (P21_F60-69_Lay)

"I think if somebody declared something, they can always declare part of the truth, because the person who's declaring can choose. They can choose how they phrase it. They can leave things So, to me, it seems pointless." (P2_F50-59_Lay)

"But my problem with the whole setting up of a registering of interest, is the fact that it's volu Most patients won't ever question this. They will never question their doctors. So, however information there is, however available and easy you make it to find, people aren't going to look because I trust it. I trusted the medical establishment before I started working for (na organisation), and now I don't. I really don't." (P2_F50-59_Lay)

"that doesn't mean somebody's not a good person and they're not doing the right thing…you kn really believes in all the stuff…But he must be biased by that. Is that good bias? Is that bad b don't know, you know? But actually, if you saw him as a clinician, you should probably kn (P15_F50-59_Carer)

"I think that we probably need to ensure that, particularly people who are responsible for those decisions, need to be trained well in understanding what conflicts of interests are. They need reg training, regular updates, and I think that they need to be challenged on a regular basis. … I think every year you probably need to go through a conflict of interest, you know, in your work. And I say that it would be better to do it with someone, rather than by yourself" (P10_M60-69_activist)

"… most companies, you know, outside of the health care, they have to declare their interests ar you can find out what their interests are by looking at them on the Internet. Health is actually one those areas, it's very, very grey and shady and not saying that it's bad, it's grey. We haven't really done an awful lot around health and care." (P10_M60-69_activist)

"the mandatory things are problem in some ways, because people will find a way around it a[illegible] they'll find a way around it if it's not compulsory as well. Although the issue about it bein[illegible] compulsory is if you can get the message out there and somebody hasn't." (P15_F50-59_Carer)
"… public members can then raise concerns to the board of the RMC or their BMA, or the ICN and sa[illegible] you know this, this drug which we're using is seen in like a 70 to 80% uptake or an increase in prescri[illegible] Is this something normal?" (P11, lay)
I'm aware that, for example, if I were to read an article in, say, the British Medical Journal, which I d[illegible] very often, but that professionals are required, or writers are required, to declare any interests that they[illegible] So that the readers can make a judgement about whether or not that interest has influenced their resea[illegible] writing. (P21,lay)

Study Information

Hypotheses

1) It is currently unreasonably difficult for individuals to search for information about health professionals declaration/conflicts of interest given the current UK system;

2) Understanding of the potential impacts of conflicts of interest is poorly understood by professionals, students and patients/citizens.

Design Plan

Study type

Experiment - A researcher randomly assigns treatments to study subjects, this includes field or lab experiments. This is also known as an intervention experiment and includes randomized controlled trials.

Blinding

- No blinding is involved in this study.

Is there any additional blinding in this study?

n/a

Study design

We will create a list of approximately 12-15 health professionals who have given recent advice or information to citizens in the public domain and who therefore may have come to the attention of the public. These will be a mixture of healthcare officials (for example, CMOs or deputy CMOs) heads of royal colleges, professional societies, pressure groups, NICE and SIGN guideline authors, and doctor journalists/columnists. We will ask for permission from each to take part. If agreed, we will do in depth searches for declarations and conflicts of interest for each professional. We will also include professionals who are not doctors, such as prominent senior NHS managers or Chief Nurses. We will ask each professional to check their declaration against ours and from this we will create a ‘gold standard’ against which we will compare to the results of participants searches. This will include:

General Google search for job title, roles, affiliations - first 10 pages
“Firstname Lastname” + “NHS” + “private” + “clinic” + “press release”

“Firstname lastname” + “Conflicts of interest” + “declared interests”

Disclosure UK (APBI)

NHS Trust site / GP surgery site / any private clinics - search for Register of Interests, and search within, if not publicly available need to write to NHS Trust using FOI if necessary

NICE/SIGN search for 1) Firstname Lastname 2) if present, search for DOI

PubMed Firstname Lastname - search for declaration of interest

Linkedin

Companies House

Electoral Commission - personal or company donations (here)

Patents (Google Patents - patents.google.com)

LexisNexis (any mentions of companies / sponsors)

TV / Radio (via Box of Broadcasts)

Pubmed: Journal articles in last 5 years, conflicts of interest declarations

Foreign databases

French (search by beneficiary here)

ClinicalTrials

OpenPayments

We will record the time taken to complete this search and 10% will be double checked by different researchers to ensure consistency. As part of this search, we will create a rolling spreadsheet of generic locations where conflicts and declarations were found. This will help to demonstrate the workload required in a search strategy. The purpose of this search is to be as complete as possible and may require repeated iterations. This is regarded as part of the research project to investigate the hypothesis that searching for declarations is unreasonably burdensome.

We will ask the healthcare professionals who have taken part in this project to check the declaration we have created for them. We will alter it as necessary to ensure accuracy. We will document the difference between each.

We will create a website. This will

- explain the problem of finding and locating declarations/conflicts of interest of healthcare professionals.

- seek participation and permission to obtain data about their age, sex, and professional/ patient / citizen background, and how they heard about this project.

- randomly allocate each participant to one professional on the list. We will give them a small amount of information about the person in question eg 'Firstname Lastname, a doctor from the UK who is head of guidelines at NICE'.

To do this we will ask participants their

- age group

- sex

- whether they are responding as a citizen/healthcare student/patient/ healthcare professional prescriber / healthcare professional / higher specialist trainee

- we will ask healthcare professionals : speciality, research/educational/NHS/ private/academic roles, estimate how many COI forms they estimate they fill out per year

- location (UK or not)

When we ask for age bands, under 18s will not be included.

-we will ask participants about what they understand by declared interests/ conflicts and how they interpret them. We will use vignettes. For ease of analysis, these will be composed of questions answered by tick box design.

- we will ask how long they think it should take to find out a doctors declaration of interest

- to search for declarations in 10 minutes, using a timer on the website

- at the end of the time we will ask them to report what they find

- we will then compare the declarations 'gold standard" which we have previously listed to participants findings. Disagreement will be resolved

through discussion. The researchers will be blinded to the age, sex, professional background and email address of the participant (if applicable).

- we will then analyse the information. We will assess a) the frequency of matching of gold standard declarations/conflicts against 1) our findings 2) the findings of the participants. Because these will vary in amount, we will express the overlap in percentage terms.

- we will ask permission to contact via email for participation in interview based qualitative research at a later date. Email addresses which are not associated with a wish to be notified of the results/opportunities will be destroyed at the end of the research period.

-We will run this methodology as a pilot with small amounts of diverse participants, to refine questions, website layout, ease of access, and with feedback. We will then roll it out as planned to a wider audience.

We will contact a representative sample of the participants who have indicated that they would be willing to be interviewed. We will aim for diversity in age, sex, and professional role, and in people with divergent answers in the questionnaires.

We will proceed to interview these participants remotely with experienced qualitative researchers. We will aim to understand what people view a conflict as, how these should be managed, and therefore what factors should be accounted for when policies are made on this. We will use semi-structured interviews, with the ‘think aloud’ method in order to draw themes which will then be analysed.
No files selected
Randomization
No response

Sampling Plan
Existing Data
Registration prior to creation of data
Explanation of existing data
n/a
Data collection procedures
There are three groups of participants.
Conflicts of interest subjects: 12-15 health professionals who have given recent advice or information to citizens in the public domain and who therefore may have come to the attention of the public. These will be a mixture of healthcare officials (for example, CMOs or deputy CMOs) heads of

royal colleges, professional societies, pressure groups, NICE and SIGN guideline authors, and doc-tor journalists/columnists.
Citizens (COI participants) - via invitations through social media, and citizens organisations; patients - via campaigning organisations such as Sling the Mesh, with invitations to organisations including The Patient Association, National Voices, social media. Healthcare students - via requests to university and college mailing lists for dissemination, social media. Higher specialist trainees - via social media, and requests to disseminate via Royal Colleges and NHS Trusts. The Medical Schools Council have been asked for permission to contact medical students subject to ethics approval and contact will not otherwise proceed without it.
Healthcare professionals - via requests to GP practices via Scottish Primary Care Research Network, social media and professional forums. The healthcare professionals we ask to take part as subjects will be based pragmatically on well known doctors and approached individually.
Qualitative research group: a representative sample of the citizen group, likely to be around 30 participants.

No files selected

Sample size

For the citizen group (COI participants) o have a confidence interval of 95% with a 5% margin, with a proportion of 0.5, we need a sample size of 385 citizens (out of a UK population of 66 million). By asking for the professional / patient / citizen identity of participants, we will draw conclusions about groups if the sample size allows.

Sample size rationale

The number of COI subjects is relatively small as each requires the creation of a 'gold standard' COI statement for comparison to be made. Larger samples would require excessive resources for little benefit.

Stopping rule

We will be time limited and aim to have enough participants to achieve the sample size within 4 weeks.

Variables

Manipulated variables

n/a

No files selected

Measured variables

1) Outcomes of vignettes (percieved trust with declared interests)
2) Comparison of participant searches with our 'gold standard' searches
3) Agreement of research searches with COI-subject declarations

Narrative analysis of qualitative interviews

No files selected

Indices

n/a

No files selected

Analysis Plan

Statistical models

Simple tabulation for analysis of responses to vignettes.
Tabulation with percentage agreement for COI searches.

We will perform a thematic analysis of the qualitative interviews.

No files selected

Transformations

n/a

Inference criteria

n/a

Data exclusion

n/a

Missing data

We will include partial data with an explanatory note.

Exploratory analysis

n/a

Other

Other

The Cumberlege review (2020) investigated the harms caused by surgical mesh to patients. It concluded that serious problems are caused by a lack of transparency of conflicts of interest (1). They found that surgical mesh was used and promoted by clinicians with financial interests in the product, even though it was inadequately tested. The review recommended that doctors should make statutory disclosures of interests. Such interests would include, for example, consultancy payments from pharmaceutical companies, patents for technological devices, or shareholding in device companies. The General Medical Council already recommends that doctors are open and transparent about their interests. Previous enquiries, for example, the Health Select Committee in the 2005 report 'The Influence of the Pharmaceutical Industry', recommended that all healthcare professionals make statutory declarations of interest to their regulator (2). This has not occurred.

This is a relatively small, emerging field of research. Key references were sought via Cochrane, PubMed, and 'snowball' searching of reference lists. There is evidence of widespread harm to patients and healthcare systems because of conflicts of interest. For example, guidelines dealing with opioid

prescribing for non-cancer pain (recognised as driving 'the opioid crisis') have had a "pervasive presence" of influence of the pharmaceutical industry amongst authors or organisations recieving funding from them (3). Doctors with financial conflicts of interest are more likely to have favourable views on the side effects from medications (4). Doctors receiving gifts and education from pharmaceutical sales representatives have poorer quality prescribing, and believe their peers, but not themselves, are adversely affected by conflicts(5).

Many countries mandate disclosures of interest by health professionals (6). The impact of this is unclear. There are concerns that statutory declarations, rather than reducing the negative impact of conflicts of interest, may transfer the burden of searching for, interpreting, and acting on them to patients (7). The US Sunshine Act does not appear to have improved patient knowledge of physician payments, with less than 5% of people knowing whether their own doctor has received industry payments (8). Further, there are concerns that disclosing interests may increase bias through 'moral license', when doctors believe that disclosure equals management of conflicts, and where patients and citizens believe that transparency negates bias (9). In the US, patients who are explicitly told about a doctor's financial conflicts with industry do not appear to change their attendance with the doctor, with no loss of trust (10). US patients at cancer centres have low levels of concern about doctors financial conflicts from pharmaceutical companies (11). The UK has a voluntary system, Disclosure UK, where payments to professionals from pharmaceutical companies are published annually, but most money is undeclared (12). NHS Trusts in England mandate disclosures for staff but these are incomplete and of poor quality (13). An up to date evaluation of current practice is currently being made by MM and others, early findings indicate widespread poor quality declarations.

As the NHS considers its' formal response to the Cumberlege report (14), potential actions in the recording and managing of declarations of interest require consideration. Little is known about how much knowledge patients, citizens, professionals and policymakers have regarding conflicts of interests in healthcare professionals, or locating and interpreting this information. A 2016 systematic review on knowledge, beliefs and attitudes of patients and the public towards interactions between professionals and the pharmaceutical and device industry found low levels of concern about financial conflicts but did not include any studies from the UK or Europe, with the exception of Turkey (15).

There is a large gap in our understanding of the most effective way to declare and interpret conflicts of interest. This is required in order to increase

public and professional understanding of their impacts and ultimately reduce the impact of bias in healthcare and improve quality.

1. Independent Medicines and Medical Devices Review. First do no harm. 2020. https://www.immdsre-

2. The influence of the Pharmaceutical Industry. 2014/5 session Health Select Committee UK Parliament

3. Spithoff S, Leece P, Sullivan F, Persaud N, Belesiotis P, Steiner L. Drivers of the opioid crisis: An appraisal of financial conflicts of interest in clinical practice guideline panels at the peak of opioid prescribing. PLoS One. 2020;15(1):e0227045. Published 2020 Jan 24. doi:10.1371/journal.pone.0227045

4. Wang AT, McCoy CP, Murad MH, Montori VM. Association between industry affiliation and position on cardio-vascular risk with rosiglitazone: cross sectional systematic review. BMJ. 2010;340: c1344–c1344. 10.1136/bmj.c1344

5. Fickweiler F, Fickweiler W, Urbach E. Interactions between physicians and the pharmaceutical industry gen-erally and sales representatives specifically and their association with physicians' attitudes and prescribing habits: a systematic review. BMJ Open. 2017;7(9):e016408. Published 2017 Sep 27. doi:10.1136/bmjopen-2017-016408

6. The sun shines on Europe. Transparency of financial relationships in the healthcare sector. 2017 Health Ac-tion International https://haiweb.org/wp-content/uploads/2017/03/Sunshine-Act.pdf

7. Wilson M. (2014). The Sunshine Act: commercial conflicts of interest and the limits of transparency. Open medicine : a peer-reviewed, independent, open-access journal, 8(1), e10–e13.

8. Kanter GP, Carpenter D, Lehmann L, et al Effect of the public disclosure of industry payments information on patients: results from a population-based natural experiment BMJ Open 2019;9:e024020. doi: 10.1136/bmjopen-2018-024020

9. Does Conflict of Interest Disclosure Worsen Bias? PLOS Medicine 24/4/12 https://doi.org/10.1371/jour-

10. Susannah L. Rose, Sunita Sah, Raed Dweik, Cory Schmidt, MaryBeth Mercer, Ariane Mitchum, Michael Kattan, Matthew Karafa, Christopher Robertson, Patient responses to physician disclosures of industry con-flicts

of interest: A randomized field experiment, Organizational Behavior and Human Decision Processes, 2019, ISSN 0749-5978 https://doi.org/10.1016/j.obhdp.2019.03.005.

11. Hampson et al. Patients' views on financial conflicts of interest. N Engl J Med 2006; 355:2330-2337 DOI: 10.1056/NEJMsa064160

12. Kmietowicz Zosia. Disclosure UK website gives "illusion of transparency," says Goldacre BMJ 2016; 354 :i3760

13. Feldman HR, DeVito NJ, Mendel J, et alA cross-sectional study of all clinicians' conflict of interest dis-closures to NHS hospital employers in England 2015-2016BMJ Open 2018;8:e019952. doi: 10.1136/bmjopen-2017-019952

14. Macdonald Helen, McCartney Margaret, Heneghan Carl, Godlee Fiona. Doctors' conflicts of interest BMJ 2020; 370 :m3247

15. Fadlallah R et al. Knowledge, Beliefs and Attitudes of Patients and the General Public towards the inter-actions of Physicians and the Pharmaceutical and Device Industry: a systematic review. PLOS 24/8/1

Standards for Reporting Qualitative Research (SRQR)*

http://www.equator-network.org/reporting-guidelines/srqr/

Page/line no(s).

Title and abstract

**Title** - Concise description of the nature and topic of the study Identifying the study as qualitative or indicating the approach (e.g., ethnography, grounded theory) or data collection methods (e.g., interview, focus group) is recommended	page 2
**Abstract** - Summary of key elements of the study using the abstract format of the intended publication; typically includes background, purpose, methods, results, and conclusions	page 1

Introduction

**Problem formulation** - Description and significance of the problem/phenomenon studied; review of relevant theory and empirical work; problem statement	Page 1
**Purpose or research question** - Purpose of the study and specific objectives or questions	Page 1

Methods

**Qualitative approach and research paradigm** - Qualitative approach (e.g., ethnography, grounded theory, case study, phenomenology, narrative research) and guiding theory if appropriate; identifying the research paradigm (e.g., postpositivist, constructivist/ interpretivist) is also recommended; rationale**	page 3
**Researcher characteristics and reflexivity** - Researchers' characteristics that may influence the research, including personal attributes, qualifications/experience, relationship with participants, assumptions, and/or presuppositions; potential or actual interaction between researchers' characteristics and the research questions, approach, methods, results, and/or transferability	page 1 (under COIs)
**Context** - Setting/site and salient contextual factors; rationale**	page 1
**Sampling strategy** - How and why research participants, documents, or events were selected; criteria for deciding when no further sampling was necessary (e.g., sampling saturation); rationale**	page 4
**Ethical issues pertaining to human subjects** - Documentation of approval by an appropriate ethics review board and participant consent, or explanation for lack thereof; other confidentiality and data security issues	page 2

For peer review only - http://bmjopen.bmj.com/site/about/guidelines.xhtml

**Data collection methods** - Types of data collected; details of data collection procedures including (as appropriate) start and stop dates of data collection and analysis, iterative process, triangulation of sources/methods, and modification of procedures in response to evolving study findings; rationale**	page 5
**Data collection instruments and technologies** - Description of instruments (e.g., interview guides, questionnaires) and devices (e.g., audio recorders) used for data collection; if/how the instrument(s) changed over the course of the study	page 5
**Units of study** - Number and relevant characteristics of participants, documents, or events included in the study; level of participation (could be reported in results)	page 6, Table 1
**Data processing** - Methods for processing data prior to and during analysis, including transcription, data entry, data management and security, verification of data integrity, data coding, and anonymization/de-identification of excerpts	page 6
**Data analysis** - Process by which inferences, themes, etc., were identified and developed, including the researchers involved in data analysis; usually references a specific paradigm or approach; rationale**	page 8
**Techniques to enhance trustworthiness** - Techniques to enhance trustworthiness and credibility of data analysis (e.g., member checking, audit trail, triangulation); rationale**	page 8

Results/findings

**Synthesis and interpretation** - Main findings (e.g., interpretations, inferences, and themes); might include development of a theory or model, or integration with prior research or theory	page 8/9
**Links to empirical data** - Evidence (e.g., quotes, field notes, text excerpts, photographs) to substantiate analytic findings	page 8/9, appendix 9

Discussion

**Integration with prior work, implications, transferability, and contribution(s) to the field -** Short summary of main findings; explanation of how findings and conclusions connect to, support, elaborate on, or challenge conclusions of earlier scholarship; discussion of scope of application/generalizability; identification of unique contribution(s) to scholarship in a discipline or field	page 10,11,12
**Limitations** - Trustworthiness and limitations of findings	page 13

Other

**Conflicts of interest** - Potential sources of influence or perceived influence on study conduct and conclusions; how these were managed	page 2

For peer review only

**Funding** - Sources of funding and other support; role of funders in data collection, interpretation, and reporting	page 2

*The authors created the SRQR by searching the literature to identify guidelines, reporting standards, and critical appraisal criteria for qualitative research; reviewing the reference lists of retrieved sources; and contacting experts to gain feedback. The SRQR aims to improve the transparency of all aspects of qualitative research by providing clear standards for reporting qualitative research.

**The rationale should briefly discuss the justification for choosing that theory, approach, method, or technique rather than other options available, the assumptions and limitations implicit in those choices, and how those choices influence study conclusions and transferability. As appropriate, the rationale for several items might be discussed together.

Reference:

O'Brien BC, Harris IB, Beckman TJ, Reed DA, Cook DA. **Standards for reporting qualitative research: a synthesis of recommendations.** *Academic Medicine*, Vol. 89, No. 9 / Sept 2014 DOI: 10.1097/ACM.0000000000000388

For peer review only

Checklist for Reporting Of Survey Studies (CROSS)

Section/topic	Item	Item description	Reported on page #
**Title and abstract**			
Title and abstract	1a	State the word “survey” along with a commonly used term in title or abstract to introduce the study’s design.	included in ‘mixed method’
	1b	Provide an informative summary in the abstract, covering background, objectives, methods, findings/results, interpretation/discussion, and conclusions.	page 1
**Introduction**			
Background	2	Provide a background about the rationale of study, what has been previously done, and why this survey is needed.	page 3
Purpose/aim	3	Identify specific purposes, aims, goals, or objectives of the study.	
**Methods**			
Study design	4	Specify the study design in the methods section with a commonly used term (e.g., cross-sectional or longitudinal).	page 1
Data collection methods	5a	Describe the questionnaire (e.g., number of sections, number of questions, number and names of instruments used).	page 4
	5b	Describe all questionnaire instruments that were used in the survey to measure particular concepts. Report target population, reported validity and reliability information, scoring/classification procedure, and reference links (if any).	page 4/appendix 2,3,4,5, 6
	5c	Provide information on pretesting of the questionnaire, if performed (in the article or in an online supplement). Report the method of pretesting, number of times questionnaire was pre-tested, number and demographics of participants used for pretesting, and the level of similarity of demographics between pre-testing	page 5

For peer review only

		participants and sample population.	
	5d	Questionnaire if possible, should be fully provided (in the article, or as appendices or as an online supplement).	appendix 3-6
Sample characteristics	6a	Describe the study population (i.e., background, locations, eligibility criteria for participant inclusion in survey, exclusion criteria).	page 6
	6b	Describe the sampling techniques used (e.g., single stage or multistage sampling, simple random sampling, stratified sampling, cluster sampling, convenience sampling). Specify the locations of sample participants whenever clustered sampling was applied.	page 6
	6c	Provide information on sample size, along with details of sample size calculation.	page 6
	6d	Describe how representative the sample is of the study population (or target population if possible), particularly for population-based surveys.	page 6
Survey administration	7a	Provide information on modes of questionnaire administration, including the type and number of contacts, the location where the survey was conducted (e.g., outpatient room or by use of online tools, such as SurveyMonkey).	page 4
	7b	Provide information of survey's time frame, such as periods of recruitment, exposure, and follow-up days.	protocol, appendix 11, and protocol deviation page 16.
	7c	Provide information on the entry process:<br>–>For non-web-based surveys, provide approaches to minimize human error in data entry.<br>–>For web-based surveys, provide approaches to prevent "multiple participation" of participants.	discussed on page 14
Study preparation	8	Describe any preparation process before conducting the survey (e.g., interviewers' training process, advertising the survey).	n/a
Ethical considerations	9a	Provide information on ethical approval for the survey if obtained, including informed consent, institutional review board [IRB] approval, Helsinki declaration, and good clinical practice [GCP] declaration (as appropriate).	page 2

For peer review only

	9b	Provide information about survey anonymity and confidentiality and describe what mechanisms were used to protect unauthorized access.	page 2 (inherent in ethical approvals)
Statistical analysis	10a	Describe statistical methods and analytical approach. Report the statistical software that was used for data analysis.	n/a simple analysis only
	10b	Report any modification of variables used in the analysis, along with reference (if available).	n/a
	10c	Report details about how missing data was handled. Include rate of missing items, missing data mechanism (i.e., missing completely at random [MCAR], missing at random [MAR] or missing not at random [MNAR]) and methods used to deal with missing data (e.g., multiple imputation).	Survey breakdown of responses in results, pages 6/7
	10d	State how non-response error was addressed.	
	10e	For longitudinal surveys, state how loss to follow-up was addressed.	
	10f	Indicate whether any methods such as weighting of items or propensity scores have been used to adjust for non-representativeness of the sample.	
	10g	Describe any sensitivity analysis conducted.	
**Results**			
Respondent characteristics	11a	Report numbers of individuals at each stage of the study. Consider using a flow diagram, if possible.	page 6/7
	11b	Provide reasons for non-participation at each stage, if possible.	page 7, 13
	11	Report response rate, present the definition of response rate or the formula used to	n/a

For peer review only

	c	calculate response rate.	
	11 d	Provide information to define how unique visitors are determined. Report number of unique visitors along with relevant proportions (e.g., view proportion, participation proportion, completion proportion).	n/a (see page 6/7)
Descriptive results	12	Provide characteristics of study participants, as well as information on potential confounders and assessed outcomes.	page 6/7
Main findings	13 a	Give unadjusted estimates and, if applicable, confounder-adjusted estimates along with 95% confidence intervals and p-values.	n./a
	13 b	For multivariable analysis, provide information on the model building process, model fit statistics, and model assumptions (as appropriate).	n/a
	13 c	Provide details about any sensitivity analysis performed. If there are considerable amount of missing data, report sensitivity analyses comparing the results of complete cases with that of the imputed dataset (if possible).	n/a
**Discussion**			
Limitations	14	Discuss the limitations of the study, considering sources of potential biases and imprecisions, such as non-representativeness of sample, study design, important uncontrolled confounders.	page 16
Interpretations	15	Give a cautious overall interpretation of results, based on potential biases and imprecisions and suggest areas for future research.	page 16/17
Generalizability	16	Discuss the external validity of the results.	
**Other sections**			
Role of funding source	17	State whether any funding organization has had any roles in the survey's design, implementation, and analysis.	n/a
Conflict of interest	18	Declare any potential conflict of interest.	page 2
Acknowledgements	19	Provide names of organizations/persons that are acknowledged along with their contribution to the research.	page 2, 16

For peer review only

For peer review only
